# Expansion of the Transporter-Opsin-G protein-coupled receptor superfamily with five new protein families

**DOI:** 10.1371/journal.pone.0231085

**Published:** 2020-04-22

**Authors:** Arturo Medrano-Soto, Faezeh Ghazi, Kevin J. Hendargo, Gabriel Moreno-Hagelsieb, Scott Myers, Milton H. Saier

**Affiliations:** 1 Department of Molecular Biology, Division of Biological Sciences, University of California, San Diego, La Jolla, California, United States of America; 2 Department of Biology, Wilfrid Laurier University, Waterloo, Ontario, Canada; UMR-S1134, INSERM, Université Paris Diderot, INTS, FRANCE

## Abstract

Here we provide bioinformatic evidence that the Organo-Arsenical Exporter (ArsP), Endoplasmic Reticulum Retention Receptor (KDELR), Mitochondrial Pyruvate Carrier (MPC), L-Alanine Exporter (AlaE), and the Lipid-linked Sugar Translocase (LST) protein families are members of the Transporter-Opsin-G Protein-coupled Receptor (TOG) Superfamily. These families share domains homologous to well-established TOG superfamily members, and their topologies of transmembranal segments (TMSs) are compatible with the basic 4-TMS repeat unit characteristic of this Superfamily. These repeat units tend to occur twice in proteins as a result of intragenic duplication events, often with subsequent gain/loss of TMSs in many superfamily members. Transporters within the ArsP family allow microbial pathogens to expel toxic arsenic compounds from the cell. Members of the KDELR family are involved in the selective retrieval of proteins that reside in the endoplasmic reticulum. Proteins of the MPC family are involved in the transport of pyruvate into mitochondria, providing the organelle with a major oxidative fuel. Members of family AlaE excrete L-alanine from the cell. Members of the LST family are involved in the translocation of lipid-linked glucose across the membrane. These five families substantially expand the range of substrates of transport carriers in the superfamily, although KDEL receptors have no known transport function. Clustering of protein sequences reveals the relationships among families, and the resulting tree correlates well with the degrees of sequence similarity documented between families. The analyses and programs developed to detect distant relatedness, provide insights into the structural, functional, and evolutionary relationships that exist between families of the TOG superfamily, and should be of value to many other investigators.

## Introduction

Establishing the molecular functions of transport proteins and elucidating their evolutionary relationships can promote breakthroughs in biotechnology, pave the way for the discovery of new drugs, and allow the development of a more comprehensive understanding of the mechanisms responsible for attaining cellular and organismal homeostasis [1, 2–6]. One of the current efforts in our laboratory is to identify distant relationships between transport proteins and classify their families into Superfamilies. There are more than 1,300 currently recognized TC families from which we have identified over 66 Superfamilies; see the superfamily hyperlink in the Transporter Classification Database (TCDB; http://tcdb.org/) [7].

Transporters play roles in numerous processes essential for life. The Transporter-Opsin-G protein-coupled receptor (TOG) Superfamily is exceptionally diverse, including channels, secondary carriers, and primary active transporters [8]. Current TOG members are found in all domains of life (Table 1) and have related topologies, usually exhibiting on average 7 or 8 α-helical transmembrane segments (TMSs) that originated from a 4-TMS precursor following a duplication event, often followed by loss of one or more TMSs [8–10].

**Table 1:** Properties of representative established members of the TOG superfamily included in this study.

Family name^‡^	TCDB accession	Average protein size (± SD)	Typical No. of TMSs	Common TMS topology	Topology description	Domain of life
LCT	2.A.43	312 ±76	7	3+4	8 TMS arose by duplication of 4 TMS (4+4). 7 TMS resulted by loss of the N-terminal TMS.	Bacteria, Eukaryota
TSUP	2.A.102	280 ±57	7–9	4+4	7 TMS arose by loss of the N-terminal TMS.	Archaea, Bacteria, Eukaryota
				3+4		
Sweet	2.A.123	209 ±106	3,7	3+4	7 TMS arose by loss of the N-terminal TMS. 3 arose from 4 TMS with loss of the N-terminal TMS.	Archaea, Bacteria, Eukaryota
				3		
MR	3.E.1	265 ±76	7	3+4	7 TMS arose by loss of the N-terminal TMS.	Archaea, Bacteria, Eukaryota
HelioR	3.E.3	263 ±31	7	3+4	7 TMS arose by loss of the N-terminal TMS.	Bacteria, Eukaryota
NiCoT	2.A.52	356 ±70	8	4+4	Duplication of 4 TMS.	Archaea, Bacteria, Eukaryota
OST	2.A.82	462 ±153	8	4+4	Duplication of 4 TMS.	Eukaryota
GPCR	9.A.14	429±157	7	3+4	7 TMS arose by loss of the N-terminal TMS.	Eukaryota Viruses

^**‡**^ Full family names and descriptions can be found in the list of abbreviations and the text.

We have improved our methods by incorporating additional requirements to detect distant homology between pairs of transporter families. We rely on the transitivity property of homology (if protein A is homologous to protein B, B is homologous to protein C, and protein C is homologous to protein D, then A is homologous to D) to infer homology between families, where the following requirements should be satisfied by pairs of candidate homologs: 1) significant sequence similarity; 2) topological agreement consistent with the TMS repeat units of the families involved; 3) overlap of the characteristic domains of both families in the alignment, 4) conservation of sequence motifs, and 5) structural similarity consistent with the repeat units of both families if 3D structures are available (See section: Detection of homology between pairs of families). Given the significant growth of publicly available sequence data since our first publication [8], we also tested the membership of all previously established families to the TOG superfamily. Families PNaS (TC: 2.A.58) and PnuC (TC: 4.B.1) failed to satisfy our stricter criteria and were removed from TOG. The alignments between PNaS homologs and other TOG members failed to satisfy criterion 2 above where the TMSs that aligned were not in agreement with their corresponding repeat units. Family PnuC also failed to satisfy the compatibility of repeat units with other TOG families at both the sequence and 3D structural levels (See discussion in section: Anomalies for previous members of the TOG superfamily). Furthermore, we identified five additional families that met these new requirements and were thus incorporated into the TOG superfamily (Table 2).

**Table 2:** New families added to the TOG superfamily.

Family name^‡^	TCDB accession	Average protein size (± SD)	Typical No. of TMSs	Common TMS topology	Topology description	Domain of Life
ArsP	2.A.119	344 ±53	8–13	4+4	8 TMSs resulted by duplication of 4 TMSs. 10 or 12 TMSs arose by insertion of 2 or 4 TMSs between the two 4-TMS halves. ^†^	Bacteria, Archaea
				4+N+4		
KDELR	9.B.191	258 ±59	7–8	4+4	7 TMSs arose by loss of the N-terminal TMS.	Eukaryota, Bacteria
				3+4		
MPC	2.A.105	133 ±32	3	4	3 TMS arose by loss of the N-terminal TMS.	Eukaryota
				3		
AlaE	2.A.104	149 ±22	4	4	Basic repeat unit	Bacteria
LST	2.A.129	156 ±27	4	4	Basic repeat unit	Bacteria, Archaea

^**‡**^ Full family names and descriptions can be found in the list of abbreviations and the text.

^**†**^ There are additional topologies observed in family ArsP, for example 4, 3+4 and 3+N+4, where 3 indicates that the corresponding 4-TMS repeat unit lost the N-terminal TMS.

Since the first member of the ArsP family (TC: 2.A.119) was functionally characterized [11], most new members have been annotated as ‘putative permease’, or ‘permease’. Many members have 8 putative TMSs with two internal 4-TMS repeats separated by a hydrophilic loop of variable sizes, but other members may contain extra TMSs in the middle of the protein between the two repeat units, and/or at either the N- or C-termini. Members of the family appear to be restricted to prokaryotes, both bacteria and archaea. Some members are encoded by genes in operons involved in arsenate/arsenite resistance [12].

*Campylobacter jejuni*, a pathogen causing gastroenteritis in humans, is prevalent in poultry and is resistant to the organic arsenic compound, roxarsone (4-hydroxy-3-nitrobenzenearsonic acid), which has been used as a food additive in the poultry industry to promote growth. Shen et al. [11] showed that ArsP contributes to organic arsenic resistance in *Campylobacter*. Analysis of multiple *C*. *jejuni* isolates from various animal species revealed that the presence of an intact *arsP* gene is associated with elevated resistance to roxarsone. In addition, inactivation of *arsP* in *C*. *jejuni* resulted in a 4-fold reduction in the minimum inhibitory concentration (MIC) of roxarsone and nitarsone compared to the wild-type strain. Furthermore, cloning of *arsP* into a *C*. *jejuni* strain lacking a functional *arsP* gene led to 8- and 64-fold increases in the MICs of roxarsone and nitarsone, respectively. Neither mutation nor overexpression of *arsP* affected the MICs of inorganic arsenic including arsenite and arsenate. Moreover, acquisition of the *arsP* gene in *C*. *jejuni* accumulated less roxarsone than the wild type strain lacking the *arsP* gene. These results indicated that ArsP functions as an efflux transporter for extrusion of organic arsenic and contributes to resistance to these compounds in *C*. *jejuni* [11].

Members of family KDELR (TC: 9.B.191) are involved in the selective retrieval of proteins that reside in the endoplasmic reticulum (ER). The ER-Golgi system has been studied using biochemical, genetic, and electron and light microscopic techniques, leading to an understanding of many aspects of trafficking from the ER to the Golgi apparatus [13]. This includes some of the signals and mechanisms for selective retention and retrieval of ER resident proteins and export of cargo proteins. Proteins that leave the ER emerge in 'export complexes' or ER 'exit sites' and accumulate in pleiomorphic transport carriers referred to as Vesicular-tubular clusters (VTCs) or ER-Golgi intermediate compartments (ERGIC). These structures then transit from the ER to the Golgi apparatus along microtubules using the dynein/dynactin motor and fuse with the cis cisterna of the Golgi apparatus. Many proteins (including vSNAREs, ERGIC53/p58 and the KDEL receptor) must cycle back to the ER from pre-Golgi intermediates or the Golgi. Murshid and Presley [13] considered a model suggesting that this cycling occurs via 50-nm COPI-coated vesicles, and in vivo evidence that suggested that retrograde trafficking may occur via tubular structures. Intracellular membrane transport involves the coordinated engagement of a series of organelles and molecular machineries that ensure that proteins are delivered to their correct cellular locations. Due to its central position in the secretory pathway and to the large amounts of signaling molecules associated with it, the Golgi complex plays a role in this regulation. The generation of autonomous signaling by the Golgi complex in response to the arrival of cargo from the ER allows the activation of a series of signaling pathways by the cargo moving from the ER to the Golgi. This regulatory mechanism is called the Golgi control system [14]. A key player in this control system is the KDEL receptor, which retrieves chaperones back to the endoplasmic reticulum and behaves as a signaling receptor. The KDEL receptor regulates pathways involved in the maintenance of the homeostatic transport apparatus, in particular, of the Golgi complex.

Members of family MPC (TC: 2.A.105) are involved in the transport of pyruvate, the end product of glycolysis, into mitochondria. This is an essential process that provides the organelle with a major fuel source. Herzig et al. [15] reported that MPC is a heterocomplex formed by two members of a family of previously uncharacterized membrane proteins that are conserved from yeast to mammals. Members of the MPC family are in the inner mitochondrial membrane, and yeast mutants lacking MPC proteins show severe defects in mitochondrial pyruvate uptake. Coexpression of mouse MPC1 and MPC2 in *Lactococcus lactis* promoted transport of pyruvate across the membrane [15]. MPC1 and MPC2, are essential for mitochondrial pyruvate transport in yeast, *Drosophila*, and humans [16]. MPC1 and MPC2 associate to form an ~150-kilodalton complex in the inner mitochondrial membrane. Yeast and *Drosophila* mutants lacking MPC1 display impaired pyruvate metabolism, with an accumulation of upstream metabolites and depletion of tricarboxylic acid cycle intermediates. Loss of yeast MPC1 results in defective mitochondrial pyruvate uptake, and silencing of MPC1 or MPC2 in mammalian cells impairs pyruvate oxidation. A point mutation in MPC1 provides resistance to a known inhibitor of the mitochondrial pyruvate carrier. Human genetic studies of three families with children suffering from lactic acidosis and hyperpyruvatemia revealed a causal locus that mapped to MPC1, changing single amino acids that are conserved throughout eukaryotes. Thus, MPC1 and MPC2 form an essential part of the mitochondrial pyruvate carrier [16]. MPCs have been reviewed from historical and functional standpoints [17].

Members of family AlaE (TC: 2.A.104) are involved in the excretion of L-alanine from the cell. A mutant that is hypersensitive to L-alanyl-L-alanine from a non-L-alanine-metabolizing *E*. *coli* strain lacks an inducible L-alanine export system and accumulates intracellular L-alanine with a reduction in the L-alanine export rate. When the mutant was used to clone genes that complement the dipeptide-hypersensitive phenotype, two uncharacterized genes, *ygaW* and *ytfF*, and two characterized genes, *yddG* and *yeaS*, were identified [18]. Overexpression of each gene in the mutant resulted in a decrease in the intracellular L-alanine level and enhancement of the L-alanine export rate in the presence of the dipeptide, suggesting that their products function as exporters of L-alanine. Since *ygaW* had the most striking impact on both the intra- and extracellular L-alanine levels among the four genes identified, Hori et al. [18] disrupted the *ygaW* gene in the non-L-alanine-metabolizing strain. The resulting isogenic mutant showed the same intra- and extracellular L-alanine levels as observed in the dipeptide-hypersensitive mutant obtained by chemical mutagenesis. When each gene was overexpressed in the wild-type strain, which does not intrinsically excrete alanine, only the *ygaW* gene conferred on the cells the ability to excrete alanine. In addition, expression of the *ygaW* gene was induced in the presence of the dipeptide. Thus, YgaW is likely to be the physiologically most relevant exporter for L-alanine in *E*. *coli*. More recently, two charged residues (R45 and D84) were found to be essential for AlaE efflux activity [19].

Members of family LST (TC: 2.A.129) are involved in the translocation of lipid-linked glucose across the membrane and have a 4 TMS topology. *Shigella flexneri* bacteriophage SfX, SfV and SfII each has a 3-gene operon encoding a glucosyltransferase (GtrX), which is involved in full O antigen modification (serotype Y to serotype X conversion). Besides the *gtrX* gene, the other two genes in the *gtr* locus of SfX are also involved in the O antigen modification process. The first gene in the cluster (*gtrA*) encodes a small hydrophobic protein involved in the translocation of lipid-linked glucose across the cytoplasmic membrane. The second gene in the cluster (*gtrB*) encodes an enzyme catalysing the transfer of the glucose residue from UDP-glucose to a lipid carrier. The third gene (*gtrX*) encodes a bacteriophage-specific glucosyltransferase which is largely responsible for the final step, i.e., attaching the glucosyl molecules onto the correct sugar residue of the O antigen repeat unit. A three-step model for the glucosylation of bacterial O antigen has been proposed [20]. *Salmonella* phage P22 also has genes involved in serotype conversion, and they are homologous to the *Shigella* phage operons cited above [21]. *E*. *coli* also has these genes, probably because they were incorporated into the bacterial chromosome [22]. The *Shigella* SfV and SfX phage GtrX proteins have 4 TMSs [23]. The 12–2 antigen is a *S*. *enterica* subspecies I-specific LPS modification that enhances long-term intestinal colonization [24].

## Results

### Detection of homology between pairs of families

**Fig 1:**
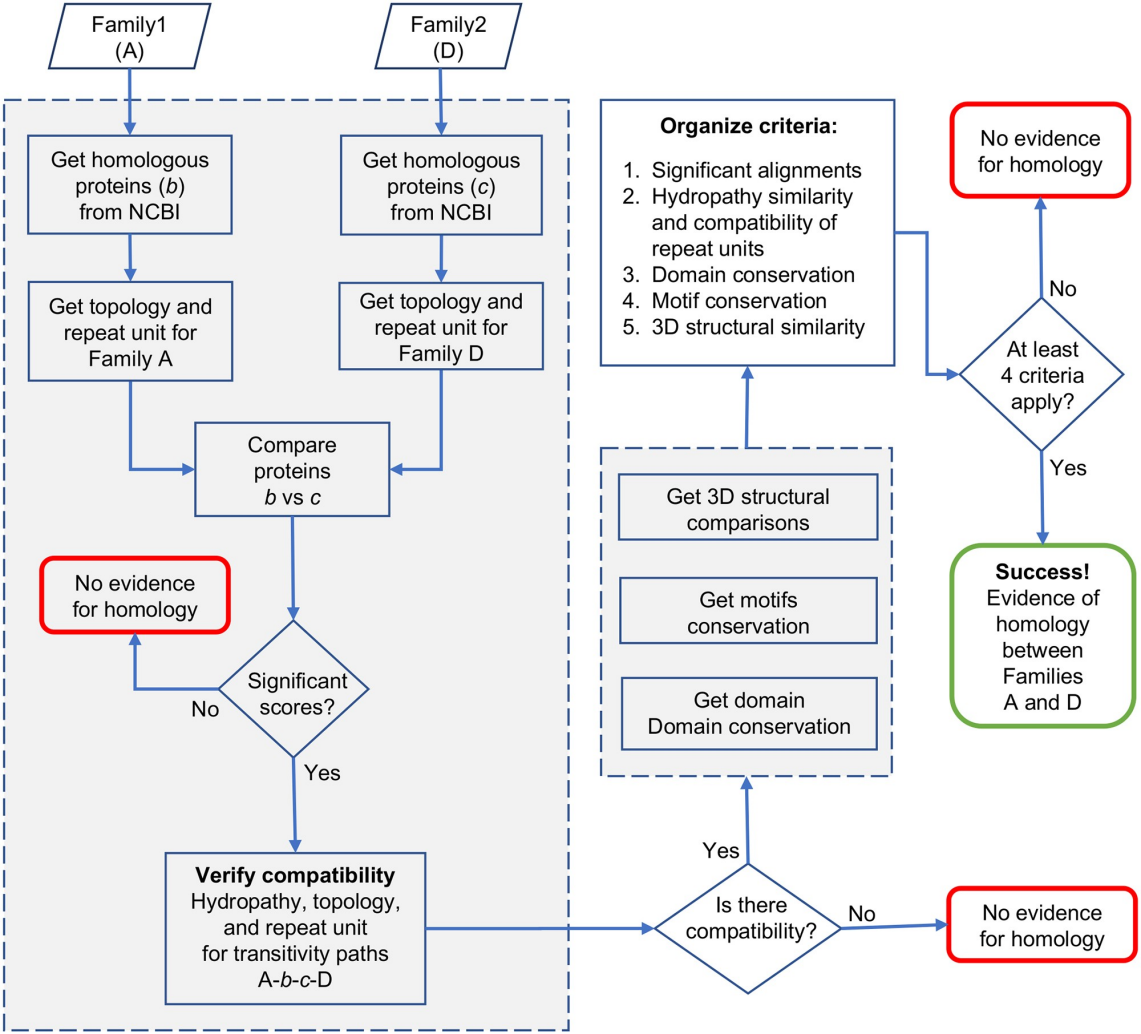
Strategy to determine whether two families of transporters in TCDB are homologous. The figure shows a simplified version of our strategy, illustrating the main steps involved and 3 points in the process where homology between two families can be rejected, with only one alternative for success (see text and Methods for details).

Fig 1 illustrates our strategy to detect homology between pairs of families based upon the transitivity principle, whereby two proteins A and D, with poor or no obvious sequence similarity, are deemed homologous if two additional proteins B (homologue of A) and C (homologue of D) can be identified such that a clear path of significant sequence similarity can be traced connecting proteins A and D (A→B→C→D). Homology is then deduced by association between the two families to which proteins A and D belong [1, 8].

Transmembranal segments may contain compositional biases and low complexity regions that inflate alignment scores (of otherwise unrelated sequences) beyond thresholds of statistical significance [25, 26]. Thus, in addition to sequence similarity across the transitivity path A→B→C→D (criterion 1), four additional criteria were applied to minimize the rate of occurrence of false positives: (2) selection of candidate homologs that show compatibility of TMS topologies and repeat units characteristic of their respective families. This step is done by manual inspection of hydropathy curves of the sequence alignment between proteins B and C to verify that there is an overlay of hydrophobic peaks, hereafter referred to as TMSs, and aligned TMSs must be congruent with the evolutionary path followed by the reference family (e.g., pore-forming TMSs, duplicated TMSs and TMSs lost/gained should correspond well in both families); (3) the characteristic Pfam domains of both families must overlap significantly in the B-C alignment, (4) shared sequence motifs between families strengthen the argument of homology; and (5) if 3D structures are available, structural superpositions consistent with the TMS repeat units of the families involved may provide additional evidence of homology. See Methods for a detailed description of the approach.

In the following discussion, the position of a protein or family within the homology transitivity path is specified by appending the corresponding letter, within parenthesis, to the end of the accession (e.g., OGD29236(B), XP_018986354(C), etc.). A and D will always refer to the original query proteins within the two TCDB families being compared, but the name of the families can also be used (e.g., LCT(D) or 2.A.43.1.1(D)). Alignments will be abbreviated by concatenating letters with dashes (e.g., the B-C alignment). E-values are calculated with the Smith-Waterman algorithm as implemented in SSEARCH [27] unless otherwise specified (See Methods).

Membership to TOG was inferred by comparing all homologous proteins within a candidate family against a positive control comprised of the set of homologs for each of the established families in the TOG superfamily (Table 1), and to a negative control set of 10 families for which no evidence of relationship to TOG has been found (see Methods and S1 Table). A family was considered a new member of TOG if at least 4 of the 5 aforementioned criteria (Fig 1) were satisfied. If no 3D structures are available for a pair of families, criterion 5 is not considered. No family within the negative control met these requirements when compared to the positive control (S2 Table). Unfortunately, only family KDELR of the new families has 3D structures available in PDB; thus, almost all evidence for homology is based on primary sequence analyses.

### New families added to TOG

**Fig 2:**
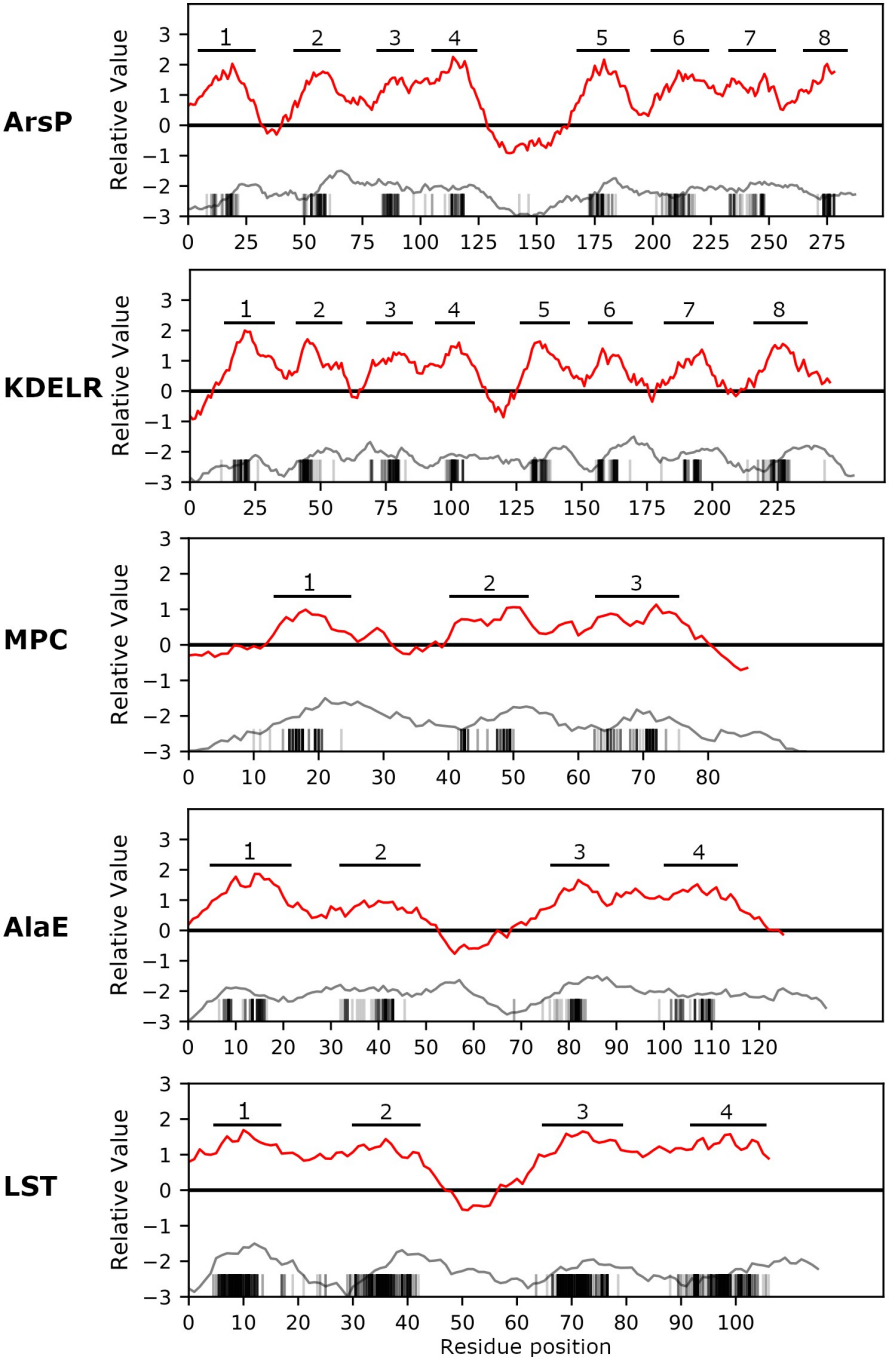
Average topological features of the five families added to the TOG superfamily. Plots were created for the new families in Table 2 with the AveHAS program [28] as described in Methods. Red curves represent average hydropathy. Hydrophobic peaks (conserved putative TMSs) are numbered. Gray curves indicate average similarity across the entire family. Vertical bars on the x-axis indicate conserved residues predicted to be in TMSs by HMMTOP [29]. Notice how the regions containing hydrophobic peaks have the highest levels of conservation.

Fig 2 shows the full-family average hydropathy plots for each of the new families being incorporated into TOG in this report and highlights conserved hydrophobic peaks in the families as putative TMSs. All families exhibit significant conservation of at least 3 TMSs consistent with the basic 4-TMS repeat unit in TOG as well as Pfam domain agreement. Candidate homologs should be compatible with the hydropathy profile of their respective families, and candidate families must have profiles compatible with the profiles of established TOG families.

#### The organo-arsenical exporter (ArsP) family (TC: 2.A.119)

**Fig 3:**
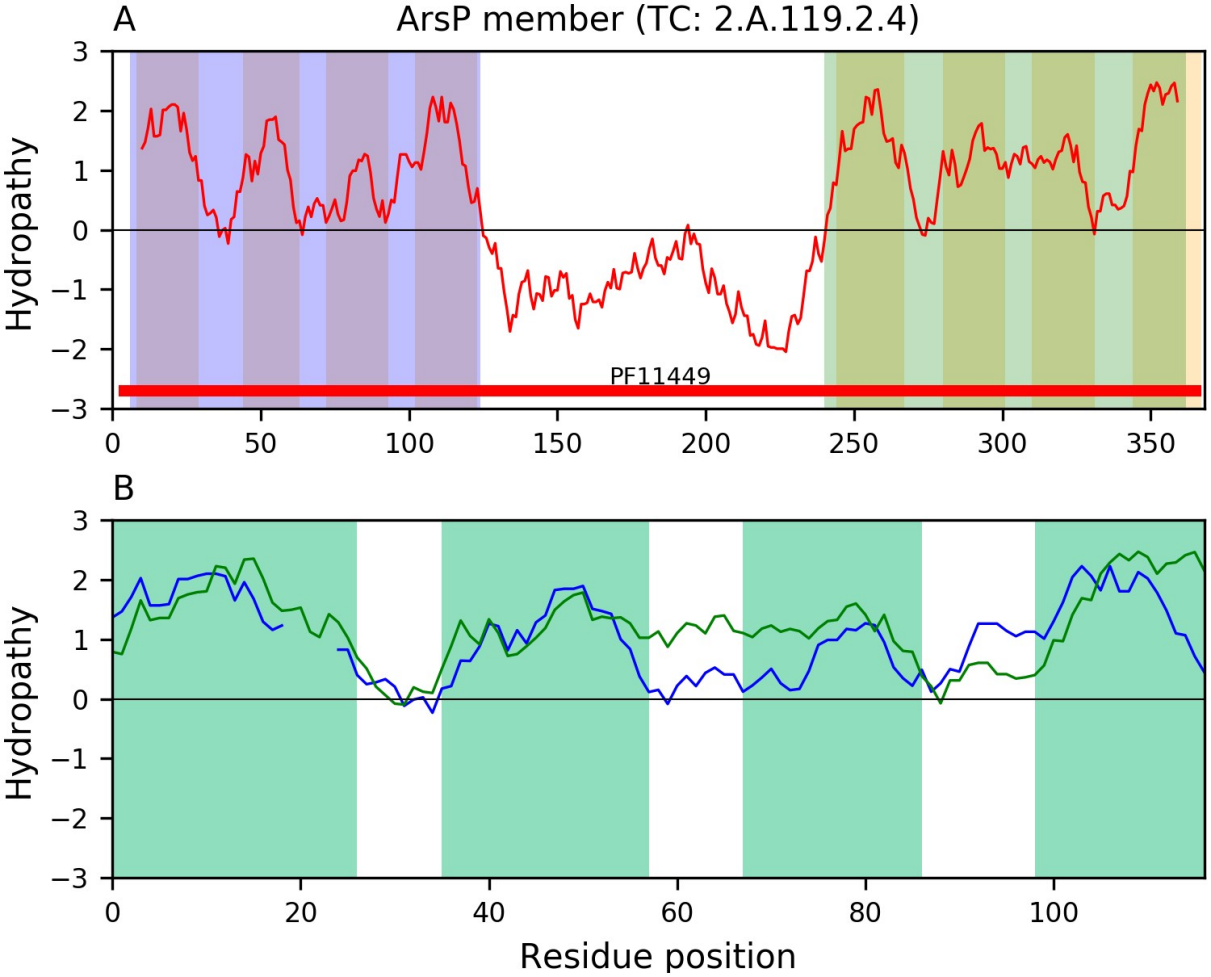
A basic 4-TMS repeat unit in family ArsP. A. Hydropathy plot of ArsP member CDE72063 (TC: 2.A.119.2.4) with 8 hydrophobic peaks highlighted in orange bars. The two 4-TMS bundles being compared are shaded blue and green, respectively. B. Alignment (E-value: 1.1×10^−8^) of the two 4-TMS bundles as identified by the program tmsRepeat (see Methods). Interruptions in the hydropathy curves indicate gaps in the sequence alignment. The blue and green curves correspond to the first and second 4-TMS bundles, respectively. For clarity, only the regions where hydrophobic peaks overlap in both curves are highlighted. Notice the correspondence of hydrophobic peaks.

The characteristic repeat unit of ArsP is 4+4 where the two 4-TMS units can be separated by regions containing 2 or 4 TMSs, but other compatible topologies are also observed. Fig 3 shows a representative alignment where the two 4-TMS halves of ArsP member CDE72063 (TC: 2.A.119.2.4) are significantly similar (E-value: 1.1×10^−8^). In this family, even more significant alignments can be found when comparing the repeats of different ArsP homologs. For example, S1 Fig shows a representative alignment (E-value: 7.8×10^−15^) between two different halves of ArsP homologs PIU02666 and WP_094226599 that provides evidence for the 4-TMS repeat unit. Members of this family may also have 7 TMSs. S2 Fig shows an alignment (E-value: 6.4×10^−44^) between 8-TMS ArsP member WP_069955515 (TC: 2.A.119.1.5) and its 7-TMS homolog KIL52798 that illustrates how the loss of the N-terminal TMS from a precursor protein with 8 TMSs explains well the origin of the 3+1+3 topology observed in ArsP and other TOG families, which can be more properly written as a 3+4 topology.

**Fig 4:**
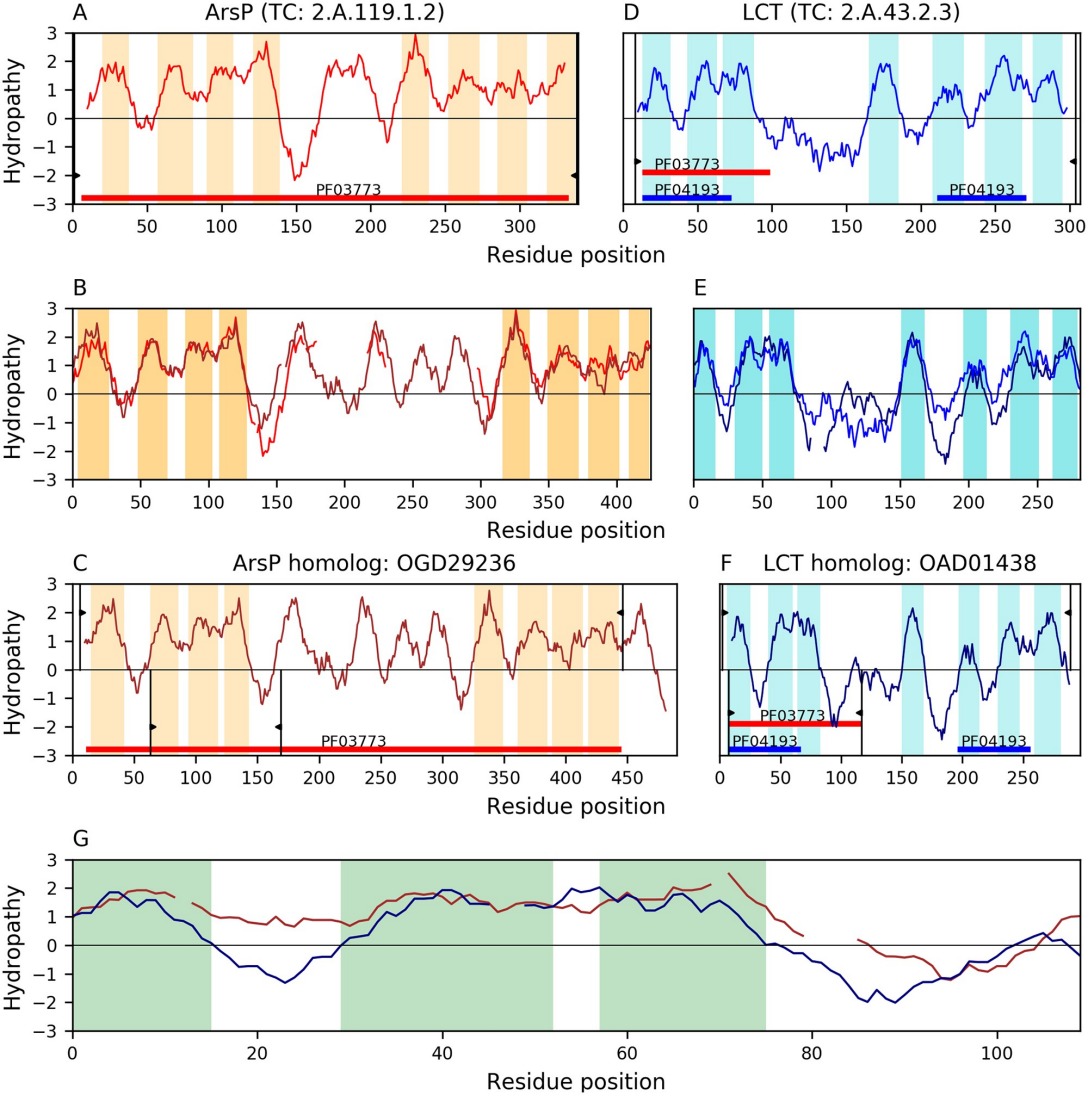
Evidence of homology between families ArsP and LCT. Hydropathy plots are presented across the homology transitivity path between families ArsP and LCT. Panels A-C depict relationships within family ArsP. Panels D-F depict relationships within the LCT family and panel G presents the evidence supporting homology between both families. Orange and Cyan bars denote hydrophobic peaks (i.e., inferred TMSs). Pfam domains are shown as colored horizontal bars. Different domain accessions within the same clan have the same color. Thin vertical black lines with wedges delimit the region of a protein involved in an alignment. The wedges in panels A and D delimit the regions covered by the alignments in panels B and E relative to the full-length proteins in panels A and D, respectively. Proteins in Panels C and F have two sets of delimiting wedges. Wedges plotted for positive hydropathy values delimit regions covered by the alignments in panel B or E relative to the full-length proteins in panels C and F, respectively. Wedges plotted for negative hydropathy values delimit regions covered by the alignment in panel G relative to the full proteins in panels C and F, respectively. Interruptions in the hydropathy curves of panels B, E, and G, indicate gaps in the corresponding sequence alignments. A. Hydropathy plot of ArsP member A8VTI4 (TC: 2.A.119.1.2). The central hydrophobicity peak in A8VTI4 corresponds to 2 TMSs as evidenced by alignments with other ArsP homologues that have two clear central hydrophobic peaks (e.g., WP_091710383, PJC47300, and PIN83468). B. Hydropathy plot of the alignment (E-value: 3.8×10^−43^) between ArsP member A8VTI4 and its homolog OGD29236. Note that OGD29236 has 4 extra TMSs in the middle, and they align with the region containing the extra central TMSs of A8VTI4. C. Hydropathy plot of ArsP homolog OGD29236. The aligned region and Pfam domain (PF03773) in OGD29236 indicate that the C-terminal TMS is extra. D. Hydropathy plot of LCT member Q12010 (TC: 2.A.43.2.3). E. Hydropathy plot of the alignment (E-value: 2.7×10^−56^) between LCT member A8VTI4 and its homolog OAD01438. F. Hydropathy plot of LCT homolog OAD01438. G. Hydropathy plot of the alignment (E-value: 1.9×10^−9^) between ArsP homolog OGD29236 and LCT homolog OAD01438. Only the regions where hydrophobic peaks overlap are highlighted in the alignments. Pfam domain PF03773 in OGD29236 can be projected to protein OAD01438 (E-value: 3×10^−6^; See Methods).

We identified significant alignments between members of the ArsP family and the established TOG families LCT, TSUP, and NiCoT. Fig 4 shows the best alignment between families ArsP(A) and LCT(D). TMSs 2–4 of the first 4-TMS repeat unit in ArsP homolog OGD29236(B) align (E-value: 1.9×10^−9^) with TMSs 1–3 of LCT homolog OAD01438(C), in agreement with the observation that 7-TMS proteins in family LCT lost the N-terminal TMS [8] (S3 Fig). All aligned TMSs are complex according to the TMSOC program and the classification proposed by Eisenhaber’s group [30], decreasing the likelihood of a false positive. Protein OGD29236(B) has 13 TMSs with topology 4+N+4+1 (N = 4), which is apparent from the region that aligns with its homologue in TCDB A8VTI4 (TC: 2.A.119.1.2; Fig 4A) and because the corresponding Pfam domain (PF03773) only covers the first 12 TMSs (Fig 4C). The aligned regions between proteins in Fig 4C and 4F show that the B-C alignment in Fig 4G is covered by the Pfam domain (PF03773) in OGD29236(B) and fully includes the 2-TMS Pfam domain (PF04193) associated with each repeat unit in LCT. In addition, we were able to project the Pfam domain PF03773 in OGD29236(B) to protein OAD01438(C) (E-value: 3×10^−6^; See Methods).

S4 Fig shows that the best B-C alignment (E-value: 8.5×10^−11^) between families ArsP(A) and TSUP(D) aligns TMSs 5–7 of ArsP member WP_082464241(B) with TMSs 5–7 of TSUP member AHF91483(C), which corresponds to the first 3 TMSs of the second repeat units in both proteins. Both proteins B and C have 8 TMS with a 4+4 topology. Only one of the aligned TMSs in the TSUP homolog is classified as simple by TMSOC [30]. Given that we found other lower scoring B-C alignments that involve complex TMSs that fully covered one repeat unit and satisfied all other criteria, we trusted the inference. The hydropathy plot of the B-C alignment shows good overlap of hydrophobic peaks (S4G Fig). The Pfam domain PF03773 in WP_082464241(B), characteristic of ArsP, can be projected to AHF91483(C) (E-value: 3×10^−6^), and it covers all 4 TMSs of the second repeat unit.

S5 Fig shows the relationship between families ArsP(A) and NiCoT(D) as evidenced by proteins WP_066228546(B) and PHH64764(C). Protein B has an extra N-terminal TMS with topology 1+4+4. The B-C alignment (E-value: 2.1×10^−8^; S5G Fig) covers the 4 TMSs that comprise the first repeat unit in both proteins. None of the aligned TMSs are simple according to the TMSOC classification [30]. NiCoT, member Q7S3L8(D) (TC: 2.A.52.1.8) and its homolog PHH64764(C) apparently have 3 TMSs in their first repeat unit, based on the identification of hydrophobic peaks. By comparing their sequences to other NiCoT homologs with 8 clear hydrophobic peaks (e.g., WP_083909747, WP_028002848, etc.), it is evident that the low-hydrophobicity region between TMSs 1 and 2 is a TMS. The presence of the second TMS is not farfetched as non-hydrophobic α-helical TMSs have been identified in 3D structures [31]. If we take this into consideration, the hydropathy of the B-C alignment shows reasonable overlap of hydrophobic peaks. In addition, we were able to project the Pfam domain (PF03773) of family ArsP onto PHH64764(C) (E-value: 1.4×10^−5^; see Methods).

#### The endoplasmic reticulum retention receptor (KDELR) family (TC: 9.B.191)

**Fig 5:**
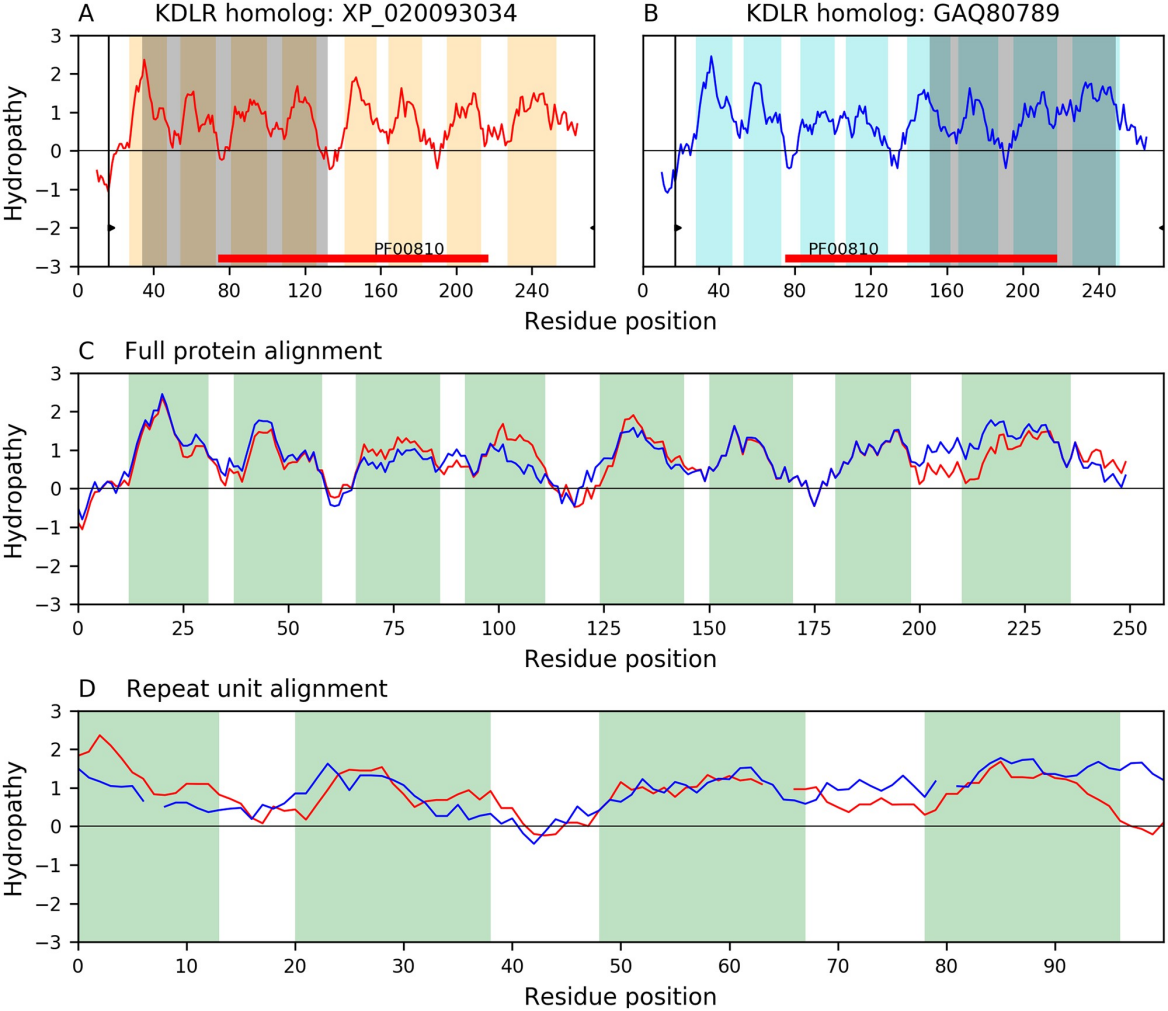
Basic repeat unit of 4 TMSs in family KDELR (TC: 9.B.191). A representative alignment between 8-TMS KDELR homologs XP_020093034 and GAQ80789, illustrates the 4-TMS repeat unit in KDELR as identified by AncientRep [32]. Thin black vertical lines with wedges delimit the regions involved in the alignment of the two full-length proteins. Orange and cyan bars highlight hydrophobicity peaks (i.e., inferred TMSs), respectively, for both proteins. A. Hydropathy plot of protein XP_020093034. TMSs 1–4 (shaded in dark gray) participate in the alignment shown in panel D. B. Hydropathy plot of protein GAQ80789. Hydrophobic peaks 5–8 (shaded in dark gray) participate in the alignment shown in panel D. C. Hydropathy plot of the alignment (E-value: 9.4×10^−102^) between the full proteins. D. Hydropathy plot of the 4-TMS alignment (E-value: 5.0×10^−4^) that provides evidence for the repeat. Interruptions in the hydropathy curves of panels C and D indicate gaps in the corresponding sequence alignments.

We identified the topology for KDELR(A) as 4+4 with borderline significance (E-value: 5.0×10^−4^; Fig 5). Alignments of comparable significance were found between members with 7 TMSs, where TMSs 1–3 aligned with TMSs 5–7 in agreement with the 3+1+3 topology observed in TOG. When KDELR members with 7 TMSs are aligned to homologues with 8 TMSs, the alignment covers TMSs 2–8 of the longer proteins, thus also supporting our claim in TOG that the 3+1+3 topology can be written as 3+4, indicating that an ancestral member with 8 TMSs lost the N-terminal TMS (S6 Fig).

**Fig 6:**
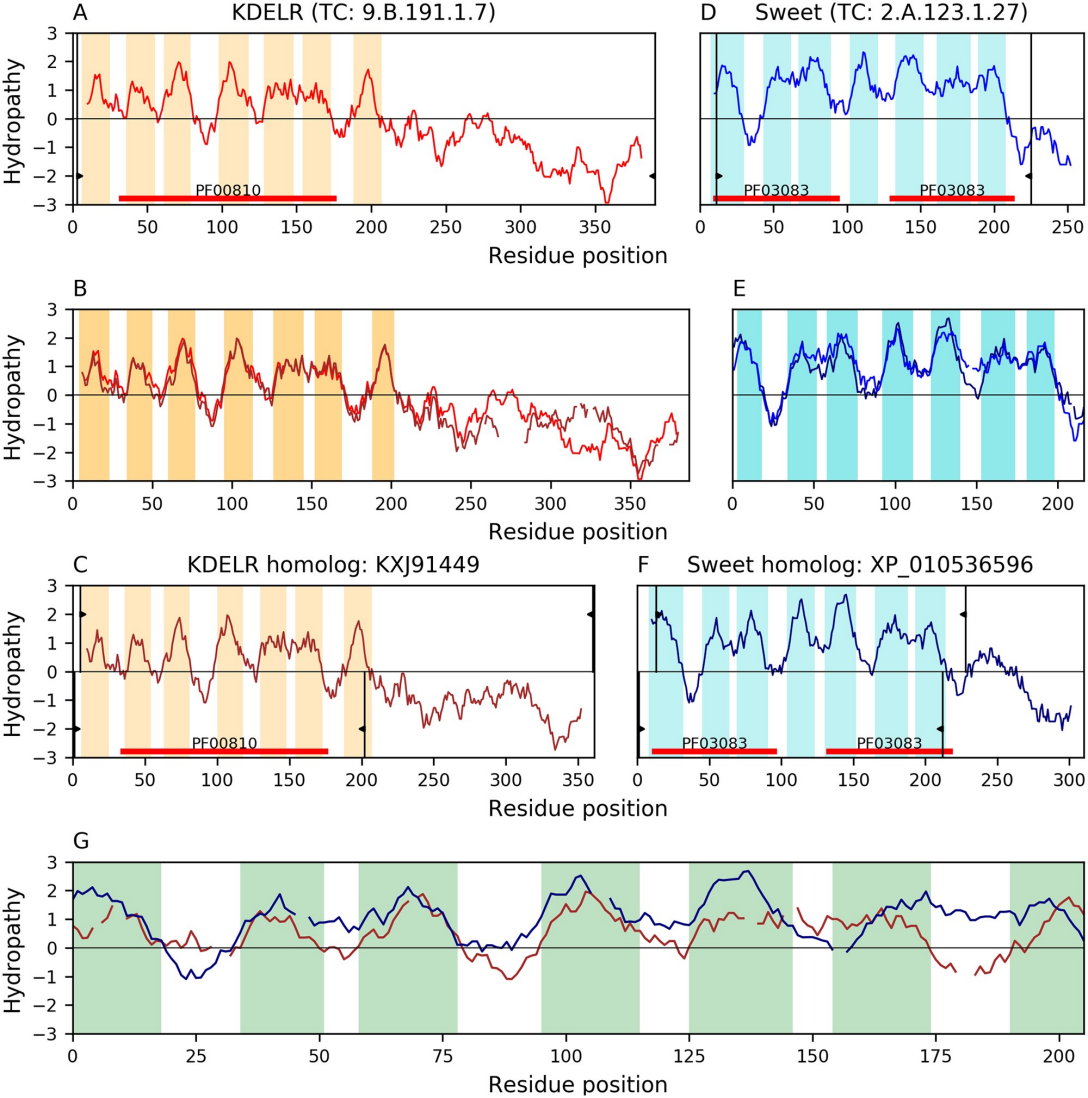
Evidence of homology between families KDELR and sweet. Hydropathy plots are presented across the homology transitivity path between families KDELR and Sweet. Refer to the legend of Fig 4 for a detailed description of the format. A. Hydropathy plot of KDELR member M7SWU9 (TC: 9.B.191.1.7). B. Hydropathy plot of the alignment (E-value: 1.7×10^−128^) between KDELR member M7SWU9 and its homolog KXJ91449. C. Hydropathy plot of KDELR homolog KXJ91449. D. Hydropathy plot of Sweet member ANC68268 (TC: 2.A.123.1.27). E. Hydropathy plot of the alignment (E-value: 5.6×10^−56^) between Sweet member ANC68268 and its homolog XP_010536596. F. Hydropathy plot of Sweet homolog XP_010536596. G. Hydropathy plot of the 7-TMS alignment (E-value: 5.3×10^−10^) between KDELR homologue KXJ91449 and Sweet homologue XP_010536596. The full Pfam domains of proteins KXJ91449 (PF00810) and XP_010536596 (PF03083) are included in the alignment and belong to the same clan (CL0141), further supporting the relationship between both families.

Family KDELR was found to be related to families Sweet and LCT within TOG. Fig 6 shows the best match (E-value: 5.3×10^−10^) between families KDELR(A) and Sweet(D), where all 7 TMSs in both proteins KXJ91449(B) and XP_010536596(C) are covered in the alignment. All TMSs in the alignment are complex according to the TMSOC classification [30]. Hydrophobic peaks overlap well while the Pfam domains of both proteins (i.e., PF00810 and PF03083) belong to the same clan (CL0141). There is one 3D structure available in family KDELR (PDB: 6I6B; TC# 9.B.191.1.8). The group that solved the structure found significant alignments (2.57–3.87 Å) of 3-helix bundles corresponding to the pore forming TMSs [33]. All together this evidence strongly supports the conclusion of homology for these two families.

S7 Fig shows the best match between family KDELR(A) and LCT(D), where TMSs 3–7 of both proteins PIA50795(B) and KXN87232(C) align (E-value: 3.1×10^−9^) and show good overlap of hydrophobic peaks (S7G Fig). All TMSs in the alignment are complex according to the TMSOC classification [30]. In this case, the characteristic Pfam domain (PF00810) of family KDELR(A) is directly identified in LCT(D) homolog KXN87232(C) (hmmscan E-value: 5.3×10^−5^) without the need of projection, thus providing further support to the relationship between both families. The alignment shown in S7G Fig does not include a full 4-TMS repeat unit in either protein, but other alignments with lower, albeit significant, quality do.

#### The Mitochondrial Pyruvate Carrier (MPC) family (TC: 2.A.105)

**Fig 7:**
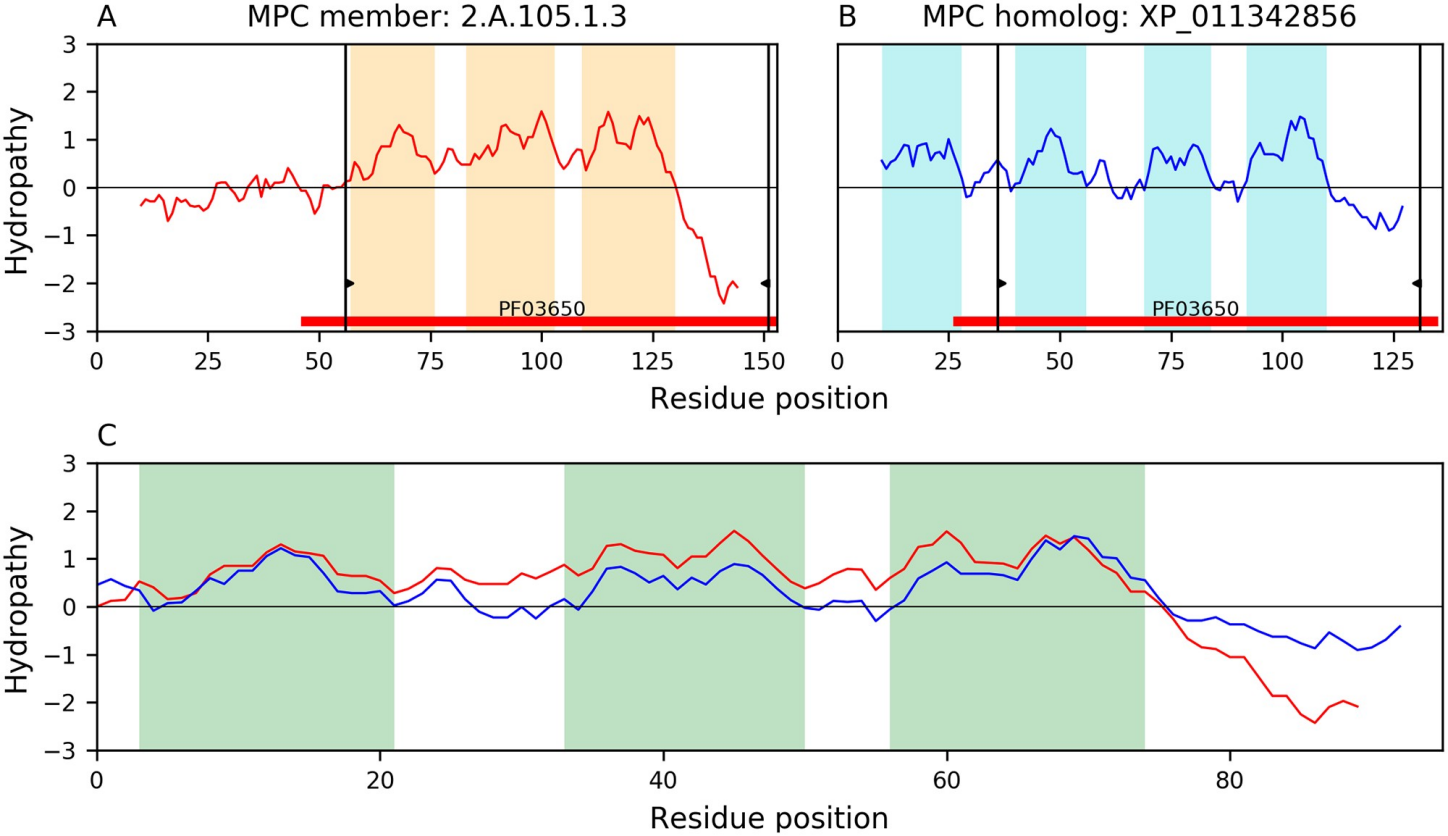
Possible origin of 3-TMS proteins in family MPC. Representative alignment of 3-TMS MPC member Q9VHB1 (TC: 2.A.105.1.3) versus the 4-TMS homologue XP_011342856. Hydropathy plots and Pfam domains for Q9VHB1 and XP_011342856 are shown in panels A and B, respectively. The hydropathy plot of the alignment (E-value: 2.4×10^−34^) between these two proteins is shown in panel C. Interruptions in the hydropathy curves of panel C indicate gaps in the sequence alignment. The regions of both proteins involved in the alignment shown in panel C, are delimited by the thin black vertical bars with wedges in panels A and B. The first TMS of XP_011342856 is not part of the alignment supporting the notion that an ancestor of protein Q9VHB1 lost its N-terminal TMS.

Members of family MPC(A) (TC: 2.A.105) typically have 3 TMSs that probably originated from a 4-TMS precursor with loss of the N-terminal TMS. Fig 7 shows a representative example of a 3-TMS MPC member Q9VHB1 (TC: 2.A.105.1.3) aligned with its 4-TMS homolog XP_011342856, where the hydropathy of the alignment (E-value: 2.4×10^−34^) suggests that members with 3 TMSs lost the N-terminal TMS as observed in several TOG families.

**Fig 8:**
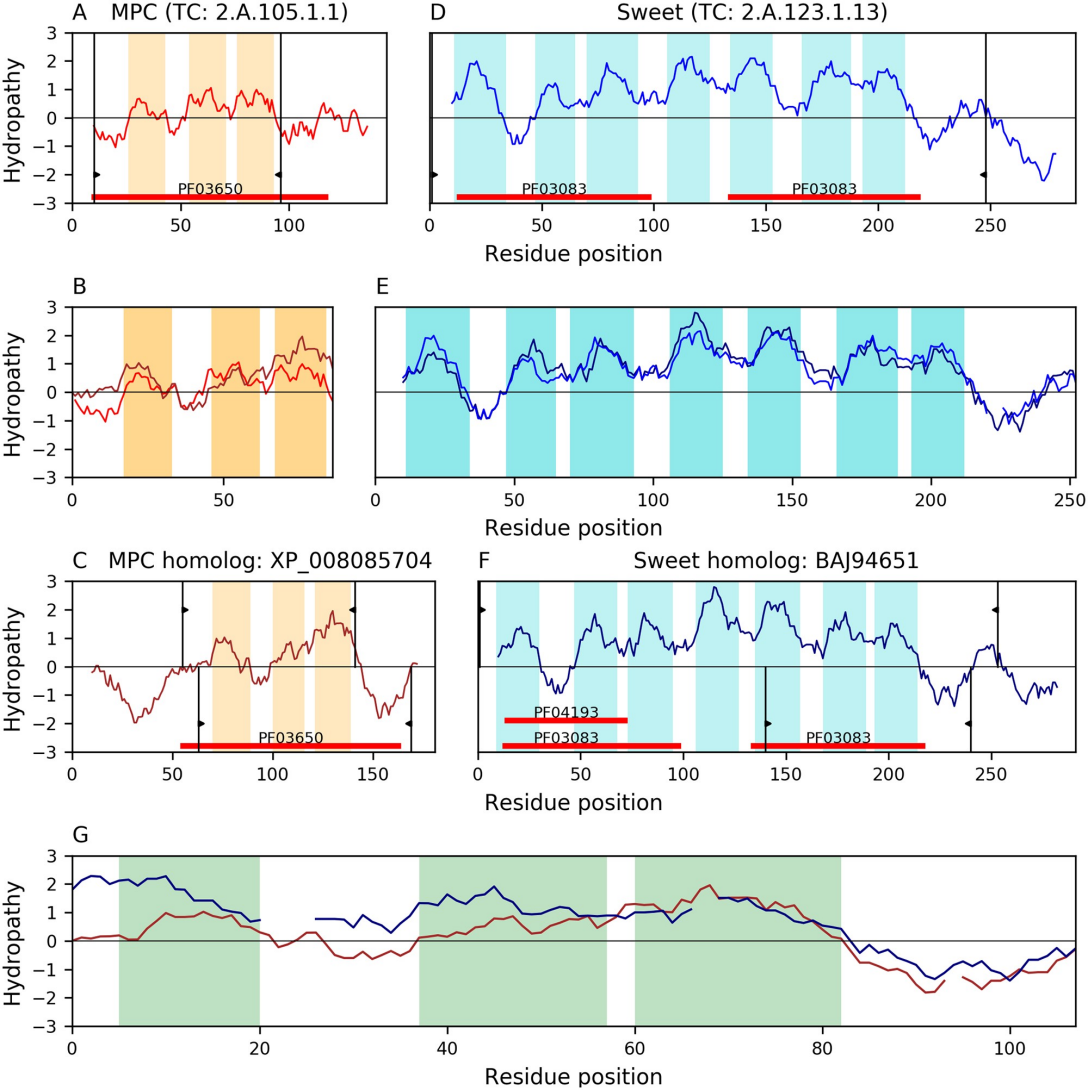
Evidence of homology between families MPC and sweet. Hydropathy plots are presented across the homology transitivity path between families MPC and Sweet. Refer to the legend of Fig 4 for a detailed description of the format. A. Hydropathy of MPC member P53311 (TC: 2.A.105.1.1). B. Hydropathy of the alignment (E-value: 2.1×10^−29^) between MPC member P53311 and its homolog XP_008085704. C. Hydropathy of MPC homolog XP_008085704. D. Hydropathy of Sweet member Q9SMM5 (TC: 2.A.123.1.13). E. Hydropathy of the alignment (E-value: 1.1×10^−71^) between Sweet member Q9SMM5 and its homolog BAJ94651. F. Hydropathy of Sweet homolog BAJ94651. G. Hydropathy of the 3-TMS alignment (E-value: 4.7×10^−10^) between MPC homolog XP_008085704 and the Sweet homolog BAJ94651. The Pfam domains of MPC (PF03650; panel C) and Sweet (PF03083; panel F) are covered in the alignment (panel G) and belong to the same clan (CL0141).

Our analyses identified a relationship between families MPC(A) and Sweet(D). Fig 8 shows that the best match (E-value: 4.7×10^−10^) aligns all 3 TMSs of MPC member XP_008085704(B) with TMSs 5–7 of the Sweet member BAJ94651(C). All TMSs involved in the alignment are complex according to the TMSOC classification [30]. This alignment is compatible with the 3+1+3 topology observed in the Sweet family because TMSs 1–3 and 5–7 comprise the two structural units that create the pore across the membrane. In agreement with the symmetry of the Sweet topology, TMSs 1–3 of XP_008085704(B) also match TMSs 1–3 of BAJ94651(C), but at much lower significance levels (E-value: 2.3×10^−3^). The characteristic Pfam domains of families MPC (PF03650) and Sweet (PF03083, PF04193) belong to the same clan (CL0141) further supporting their relationship.

There are three recognized 3-TMS MPC isoforms known as MPC1, MPC2 and MPC3 types, where MPC3 (found in yeast) shows ~75% sequence identity to MPC2 [16], while MPC1 and MPC2 are ~30% identical. These MPC types form heterocomplexes that bind other proteins, facilitating transport of pyruvate across the mitochondrial inner membrane [15, 16]. We identified several MPC proteins with 7 putative TMSs arranged in a 3+1+3 topology that we believe originated by duplication of a 4-TMS MPC precursor, followed by loss of the N-terminal TMS and sequence divergence of the two halves. However, we cannot eliminate the possibility of an event where MPC types 1 and 2 fused with insertion of the central TMS. For example, TMSs 1–3 of the 7-TMS protein C5K6B0 (TC: 2.A.105.1.8) align best (identity: 46.7%) with all 3 TMSs of the MPC1 homolog Q4N4U8 (TC: 2.A.105.1.6). On the other hand, TMSs 5–7 of C5K6B0 are most similar (Identity: 55.3%) to the 3-TMS MPC2 homolog P38857 (TC: 2.A.105.1.1). The appearance of MPC1-like and MPC2-like proteins in the same polypeptide agrees with previous observations of both isoforms being components of a functional heterocomplex [15].

#### The l-alanine exporter (AlaE) family (TC: 2.A.104)

**Fig 9:**
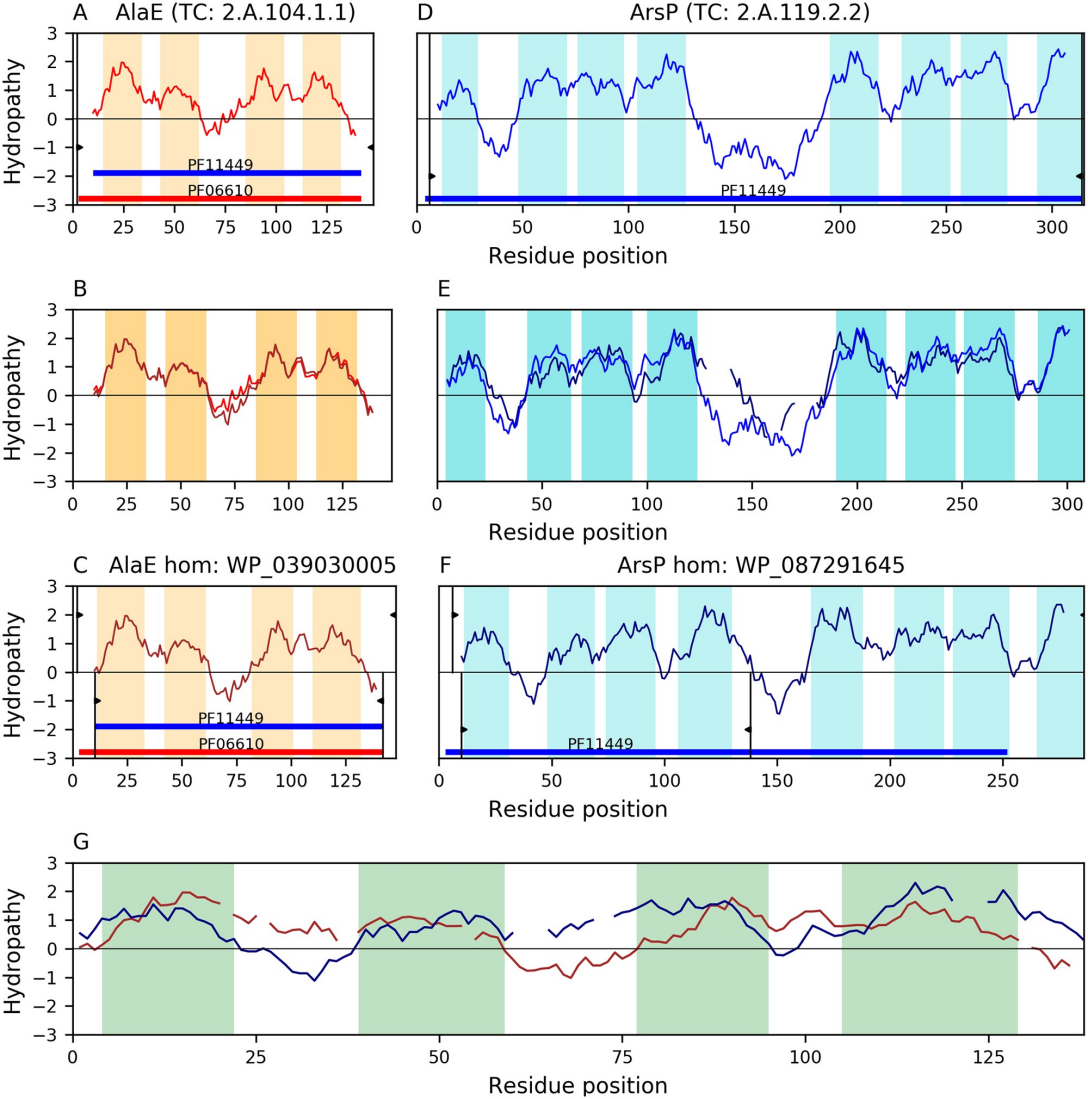
Evidence of homology between families AlaE and ArsP. Hydropathy plots are presented across the homology transitivity path between families AlaE and ArsP. To save space, the word homolog was abbreviated as “hom” in the captions of panels C and F. Refer to the legend of Fig 4 for a detailed description of the format. A. Hydropathy plot of AlaE member A8ANM6 (TC: 2.A.104.1.1). B. Hydropathy plot of the alignment (E-value: 4.4×10^−69^) between AlaE member A8ANM6 and its homolog WP_039030005. C. Hydropathy plot of AlaE homolog WP_039030005. D. Hydropathy plot of ArsP member R9KWU8 (TC: 2.A.119.2.2). E. Hydropathy plot of the alignment (E-value: 4.9×10^−61^) between ArsP member R9KWU8 and its homolog WP_087291645. F. Hydropathy plot of ArsP homolog WP_087291645. G. Hydropathy plot of the 4-TMS alignment (E-value: 1.8×10^−8^) between AlaE homolog WP_039030005 and the ArsP homolog WP_087291645. The projection (E-value: 2.4×10^−7^) of the Pfam domain in the ArsP homolog WP_087291645 (PF11449; panel F) onto the AlaE homolog WP_039030005 (panel C) covers all 4 TMSs of WP_039030005, thus providing additional evidence for homology between these families.

Members of family AlaE have 4 characteristic TMSs. Our analyses identify a relationship between families AlaE(A) and ArsP(D). Fig 9 shows the best B-C alignment between these families, where it can be appreciated (Fig 9G) that the hydropathy curve of all 4 TMSs in the AlaE(A) homolog WP_039030005(B) overlaps best (E-value: 1.8×10^**−8**^) with the 4 TMSs of the first repeat unit in ArsP(D) homolog WP_087291645(C). All TMSs involved in the alignment are complex according to the TMSOC classification [30]. We were able to project (E-value: 2.4×10^**−7**^) the Pfam domain (PF11449) of family ArsP(D) onto the full length of AlaE(A) homolog WP_039030005(B), which provides additional support to the relationship between these families.

#### The lipid-linked sugar translocase (LST) family (TC: 2.A.129)

**Fig 10:**
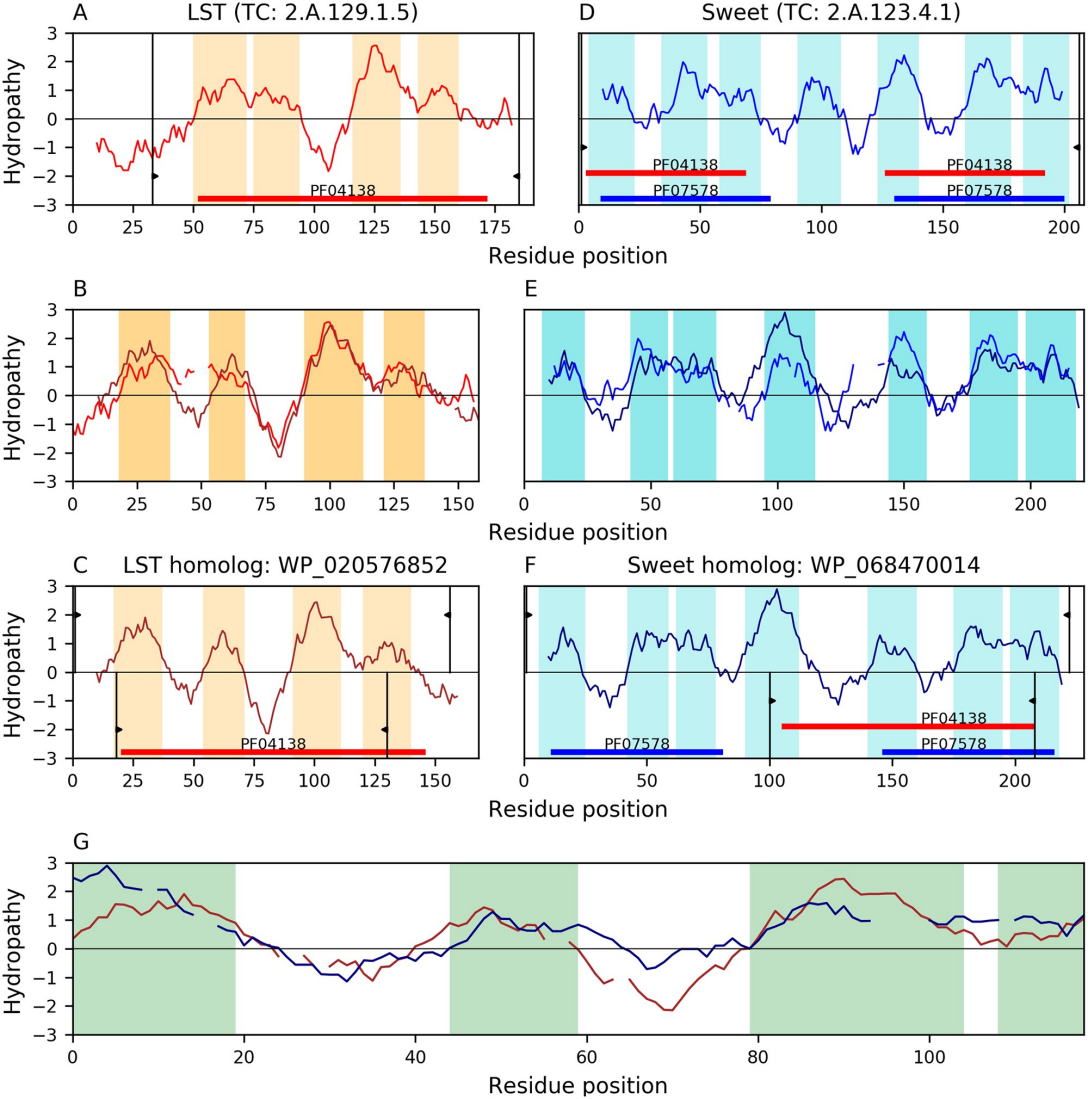
Evidence of homology between families LST and sweet. Hydropathy plots are presented across the homology transitivity path. Refer to the legend of Fig 4 for a detailed description of the format. A. Hydropathy plot of LST member SEM04082 (TC: 2.A.129.1.5). B. Hydropathy plot of the alignment (E-value: 8.8×10^−35^) between LST member SEM04082 and its homolog WP_020576852. C. Hydropathy plot of LST homolog WP_020576852. D. Hydropathy plot of Sweet member A0M0P7 (TC: 2.A.123.4.1). E. Hydropathy plot of the alignment (E-value: 6.3×10^−21^) between Sweet member A0M0P7 and its homolog WP_068470014. F. Hydropathy plot of Sweet homolog WP_068470014. G. Hydropathy plot of the alignment (E-value: 1.5×10^−8^) between LST homolog WP_020576852 and the Sweet homolog WP_068470014. Panel G and the delimited regions in Panels C and F show that 3.5 TMSs are aligned. The projection (E-value: 9.1×10^−6^) of the Pfam domain in LST (PF04138; panel C) onto the Sweet homologue WP_068470014 covers the second half of the protein (panel F), thus providing additional evidence of homology between these families.

This family has 4 characteristic TMSs. We identified a relationship between families LST(A) and Sweet (D) through the proteins WP_020576852(B) and WP_068470014(C). Fig 10G shows that the B-C alignment (E-value = 1.5×10^−8^) covers almost all 4 TMSs of protein B (Fig 10C) and the second repeat unit (TMSs 4–7) of protein C (Fig 10F), although one pair of TMSs in the alignment is simple according to the TMSOC classification [30]. Hydrophobic peaks show good overlap. This alignment is congruent with the 3+4 topology of many Sweet(D) members as they lost the N-terminal TMS from an ancestral 4+4 topology [8]. The Pfam domain (PF04138) of family LST can be projected onto Sweet member WP_068470014(D) (E-value: 9.1×10^−6^), fully covering the aligned region (Fig 10F).

Furthermore, we identified additional significant but lower scoring alignments between these two families that completely cover the second repeat unit of Sweet homologs. Given this evidence we are confident that families LST and Sweet are related.

### Anomalies for previous members of the TOG superfamily

The relationships among all families within the TOG superfamily [8] were tested to determine whether their relationships still hold in light of improved methods and significantly more sequence information available in public repositories. Families PnuC (TC: 4.B.1) and PNaS (TC: 2.A.58) failed to satisfy our improved criteria and were removed from the superfamily pending the discovery of substantiating evidence.

#### Family PnuC (TC: 4.B.1)

It has been reported that families PnuC and Sweet have 3+1+3 topologies [34, 35]. Evidence suggests that the 3+1+3 topology originated from a 4+4 original arrangement followed by loss of the N-terminal TMS [8], both in PnuC (S8 and S9 Figs) and Sweet (S10 and S11 Figs). Although some members of the PnuC family have regions of sequence similarity with members of the Sweet family, the TMSs involved in the corresponding B-C alignments cannot be reconciled with the repeat units of these families. For example, S12 Fig shows the B-C alignment (E-value: 1.8×10^**−8**^) between PnuC(A) homolog OFX33391(B) and Sweet(D) homolog OWY93661(C). Only one TMSs in the alignment is classified as simple by TMSOC [30]. Both proteins have 7 TMSs with topology 3+1+3. The B-C alignment involves TMSs 2–5 of B and TMSs 3–6 of C, which is not congruent with the topologies of these families. For this alignment to make sense, both proteins need to include at least one complete 3-TMS bundle in the alignment or align the same TMSs in both proteins. Superposition of 3D structures between members of the Sweet and PnuC families revealed that the organization and connectivity of α-helices is different, but they still generate pores of high structural similarity (RMSD ~ 2 Å) [34, 35]. This prompted the hypothesis that families Sweet and PnuC are homologous and a domain swapping event could have altered the arrangement of TMSs without disrupting the structure of the pore. However, it has been emphasized that the possibility of structural convergence could not be ruled out [35]. Understandably, controversy persists given the body of evidence supporting both alternatives [36, 37]. Thus, further work and better evidence is required to settle the issue of whether PnuC is homologous to Sweet and thus a member of TOG.

#### Family PNaS (TC: 2.A.58)

Proteins in family PNaS show a 4+4+2 characteristic topology (S13 Fig). When compared with TOG, alignments covering at least 4 TMSs were found with members of families MR, LCT and TSUP. However, none of these alignments were congruent in terms of the repeat units identified for these families. The best scoring B-C alignment (E-value:1.7×10^−8^) was identified with family MR(D), see S14 Fig, which has a 3+4 topology that originated from a 4+4 arrangement followed by loss of the N-terminal TMS [8]. This match has several problems: 1) relative to protein WP_026901115(B), the alignment starts with the sixth hydrophobic peak, or TMS 2 of the second repeat unit. On the other hand, relative to protein AJP85873(C), the alignment starts on the third hydrophobic peak, which is the fourth TMS of the first 4-TMS repeat unit given the loss of the first TMS. This makes the alignment incompatible with the repeat units; 2) the 2 extra TMSs of the PNaS homolog align with TMSs 3–4 of the second repeat unit in AJP85873(C); 3) the hydropathy curve of the B-C alignment (S14G Fig) shows that the first 2 hydrophobic peaks have little overlap; and 4) when the hydrophilic region at the C-terminus of the B-C alignment is removed, the scores of the alignment drop significantly (E-value: 3.4×10^−4^). Altogether, this evidence is not sufficient to justify the membership of family PNaS in the TOG superfamily. This exemplifies a well-known problem, namely that sequence similarity alone may not be sufficient to detect homology when comparing membrane proteins [25, 26].

### Topologically dissimilar families with unexpected sequence similarity to the TOG superfamily

#### The CaTA family (TC: 1.A.14)

Originally, we identified the CaTA family, formerly the TEGT family, as a potential TOG member due to significant sequence alignments with other TOG families (i.e., LCT and TSUP) suggesting that members of CaTA lost the C-terminal TMS. The crystal structure of the *B*. *subtilis* CaTA homologue YetJ (TC: 1.A.14.2.3) [38] revealed that in this 7-TMS protein, TMSs 1–3 and 4–6 form two units wrapped around the seventh TMS, thus presenting a 3+3+1 topology that contrasts with the characteristic 3+1+3 (or 3+4) topology in the TOG superfamily. Our sequence-based search for the repeat unit in family CaTA (see Methods) revealed alignments matching TMSs 1–3 with 4–6 in agreement with the symmetry observed in the structure (S15 Fig). 3D structural comparisons of CaTA members against all existing members of TOG yielded no reliable alignments of known repeat units (see Methods). Unless further evidence is identified, this family cannot be reliably regarded as a member of TOG, given the sequence and structural information supporting the 3+3+1 topology. Divergent evolutionary pathways sometimes yield similar 3D structures, even when homology is difficult to identify using primary sequence data [39]. Alternatively, similar sequences may produce different structures under different environments and single protein sequences can assume different structures depending on the environment in which the protein is found [40–46]. On the other hand, substantially divergent structures and sequences can never disprove homology. Nevertheless, when 3D structure and primary sequence analyses support each other, the evidence of homology is stronger. Further characterization of protein structures in different conformations and under different conditions will be necessary to better understand the relationship between families with sequence similarity but different structural topologies.

#### The NicO family (TC: 2.A.113)

Members of the NicO family, a member of the LysE superfamily, typically have 6 predicted TMSs organized in a 3+3 topology that contrasts with the known 4+4 topology in the NiCoT family [8]. Notwithstanding, significant 6-TMS alignments (E-value: 4.0×10^**−13**^) were detected between members of NicO and the TOG-member NiCoT familiy. The significance of the alignment cannot be explained by the presence of TMSs with high compositional bias because all 6 TMSs in NicO members are complex or in the twilight zone [30]. Based on our criteria (Fig 1), and despite the sequence similarity, the lack of agreement between the topologies of these two families is enough to prevent the incorporation of NicO into the TOG superfamily. We consider it possible that the 3-TMS repeat unit of NicO family members and the 4-TMS repeat unit of NiCoT family members were both derived from the same 3 or 4 TMS unit by gain or loss of a TMS. If this proves to be true, the TOG and LysE superfamilies, although potentially dissimilar at the structure level because of the different length of their repeat units, would nevertheless be homologous because they both derived from a common repeat unit. The potential formation of ultra-superfamilies will be the subject of future research.

It is interesting that the NicO and NiCoT families, each within different superfamilies and having internal repeats of 3 and 4 TMSs, respectively, exhibited significantly similar sequences, leading us to suggest that their dissimilar repeat units derived from a common origin. These two families, however, are specific for the same substrate, the divalent metal cations Ni^2+^ and Co^2+^. Two possibilities thus exist to account for their appreciable levels of sequence similarity: divergence from a common origin, or convergence to form similar heavy metal ion binding sites within unrelated transmembrane domains. While we favor the former possibility, further studies will be required to prove or disprove this conjecture.

### Integrated analysis of the TOG superfamily

#### Conservation of sequence profiles across the TOG superfamily

In our approach, we scanned for conserved sequence regions across the expanded TOG superfamily using the MEME suite [47]. Based on a training set containing 50 sequences for each TOG family we searched for sequence profiles (MEME models) 15–50 aas wide. Then, identified models were scanned against two test sets of sequences using MAST [47]. The first set consists of all non-redundant homologs extracted for the TOG superfamily, including new family additions (16,771 proteins), but excluding all proteins that directly participated in the training set. The second test set consisted of all 7,321 non-redundant homologs identified for the negative control families (See Methods). We identified one sequence profile 50 aas long that recovered homologs of all new families added in this report to TOG, as well as members of previously established TOG families, with the exception of family TSUP (TC# 2.A.102). This particular profile retrieved none of the 7,321 homologs of the negative control families. However, we also identified 4 additional MEME models that, when combined, were able to recover members of all families in TOG, including the new additions, while bringing up only 3 proteins from the negative control. These profiles were biased toward families closer to the Sweet group (e.g., LCT and KDELR), but after running MEME for different model widths and requiring that at least 100 sequences in the training set should present a conserved region, it was possible to identify profiles that map to all TOG families. S1 File presents the results of MEME and MAST in this analysis.

Besides the discriminating power, the 50 aas MEME model reveals a clear pattern. If we only consider MAST matches showing E-values < 10^−3^ and p-values < 10^−6^, a strong preference for TMSs 1–2 and 5–6 is apparent in most TOG families with 3 or 7 TMSs. These two pairs of TMSs correspond to TMSs 2–3 of the ancestral 4-TMSs repeat unit that gave rise to the TOG superfamily. That is, TMSs 1–2 correspond to TMSs 2–3 of the first 4-TMS repeat unit after loss of the first TMS, and TMSs 5–6 correspond to TMSs 2–3 of the second 4-TMS repeat unit. This is true for the vast majority of MAST hits in families Sweet (e.g., XP_011079737), LCT (e.g., CRG85333), KDELR (e.g., XP_012800521), MPC (e.g., XP_018324012), MR (e.g., SNR35428) and GPCR (e.g., XP_004476044). MAST matches are also observed with the equivalent TMSs 2–3 in 4-TMS members of families AlaE (e.g., PJC87700) and LST (e.g., WP_032638823), as well as in the 8-TMS family NiCoT (e.g., KUO02949).

To further investigate the implications of these particular segments of the proteins, we selected proteins with available 3D structures and extracted from the respective articles the annotated functional residues (i.e., substrate binding, ligand binding, etc.). Then, we mapped onto the structures the segments matched by MAST (p-values < 10^−6^) and highlighted all functional residues that fall within these regions. In the structure of xanthorhodopsin of the eubacterium *Salinibacter ruber* (PDB: 3DDL), a 7-TMS MR member and light-driven proton pump with a dual chromophore [48], the MAST hit maps to TMSs 1–2, which correspond to TMS 2–3 of the ancestral 4-TMS repeat unit. TMSs 1–2 are part of the retinal binding pocket, and a number of residues in these TMSs make contact with the retinal cofactor (S16 Fig). The structure of the 7-TMS Sweet transporter SWEET13 (PDB: 5XPD) from *Arabidopsis thaliana* [49] has a MAST hit that also covers TMSs 1–2. These TMSs have direct contact with the substrate analog, 2'-deoxycytidine 5'-monophosphate, bound in the central cavity, and 40% of the residues that bind the substrate are located in these 2 TMSs (S17 Fig). Similarly, in the structure of the *Gallus gallus* 7-TMS KDELR receptor (PDB: 6I6H) locked in the apo ER state, KDEL retrieval signal-bound Golgi state, and in complex with an antagonistic synthetic nanobody [33], the MEME profile again maps to TMSs 1–2. These TMSs contain residues directly involved in binding the KDEL ligand (S18 Fig). The top MAST match in the GPCR homolog XP_004476044 maps to TMSs 5–6. Unfortunately, this protein does not have a structure, and its closest ortholog with structure, the human C-C chemokine receptor type 5 (CCR5; PDB: 6MEO; identity: 40%) is co-crystalized with the HIV-1 envelop glycoprotein gp160 [50] and may not reflect the native binding residues. In spite of this, TMSs 5–6 in the CCR5 ortholog do have contact with the viral protein.

The consistency of top MAST matches hitting the same regions across most TOG families and the involvement of these regions in function make this sequence profile relevant. While specific functional residues are not emphasized well enough by the MEME model (thus our reluctance to call it a motif), the fact that key residues reside in these regions boosts our confidence. We should note that two families consistently have MAST hits mapping to other TMSs. The 8-TMS family ArsP maps it to TMSs 7–8 (e.g., WP_005338647) and the 7-TMS family HelioR finds it in TMSs 6–7 (e.g., KGM15768). These TMSs correspond to TMSs 3–4 of the basic 4-TMS TOG repeat unit. This suggests that the sequence profile encoded in the identified 50aa MEME model is composed of at least two similar regions as a result of the MEME model being built using the One Occurrence Per Sequence mode (OOPS), but the sequences of TMSs 1–2 and 5–6 are clearly dominant.

#### Protein tree of the TOG superfamily

**Fig 11:**
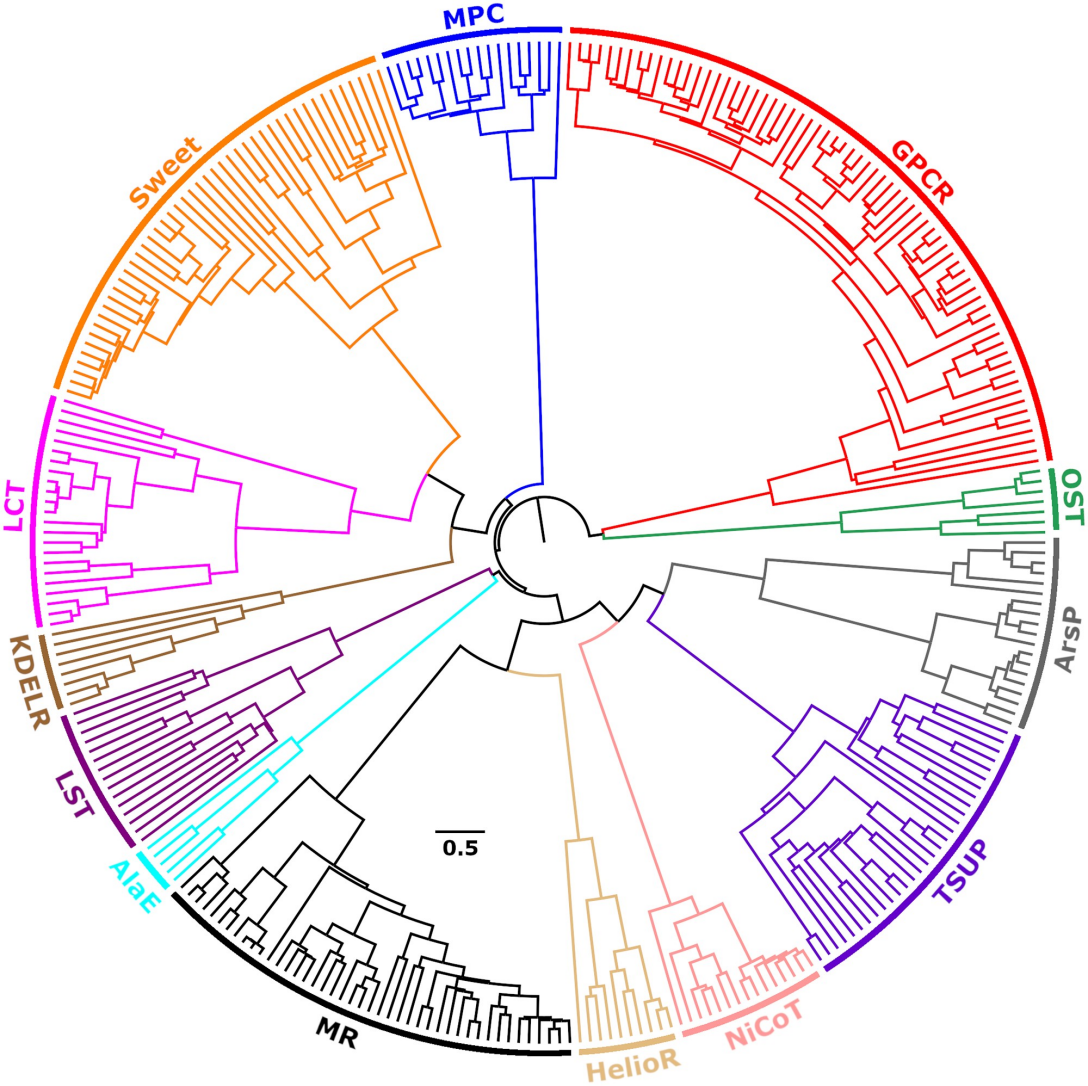
Radial tree of protein sequence similarities within the TOG superfamily. Different families are represented with different colors. The tree was generated with the program mkProteinClusters [1] based on Smith-Waterman bit scores of pairwise alignments (agglomerative coefficient: 0.88; see Methods). The original tree in Nexus format used to generate the figure is available in S3 File.

To investigate the overall relationships among the new and preexisting families within the TOG superfamily, we selected a representative set of 306 proteins (S2 File) and clustered them based on the Smith-Waterman bit scores of pairwise alignments using our program mkProteinClusters [1] (see Methods). The strength of the clustering structure is reasonable (agglomerative coefficient: 0.88). The family groupings achieved by this method have shown excellent agreement with phylogenetic trees in a previous study [1]. Fig 11 shows the radial tree layout of a dendrogram depicting the relationships within the TOG superfamily. The tree discriminates proteins in different families and suggests closer relationships a) among families Sweet, LCT and KDELR; b) between families MR and HelioR; and c) among families TSUP, NiCoT and ArsP. As shown in our results below, the confidence level of our inferences is consistent with these relationships.

Table 3 summarizes the application of all of our criteria to identify homology between the families within the original TOG superfamily (Table 1) and the newly identified candidate families in Table 2. Table 3 shows the minimal number of relationships that can be used to relate all families within the superfamily. All of the families in the negative control set failed criterion 2 (compatibility of topology and repeat units) in our methodology when compared to the new families (S2 Table).

**Table 3:** Comparison of all relevant families within TOG used in this study.

	Homology transitivity path				Alignment E-values			TMS Aln^†^
	Family A	Homolog B	Homolog C	Family D	A vs B	B vs C	C vs D	
**New members**	2.A.119.1.6 (ArsP)	WP_082464241	AHF91483	2.A.102.4.1 (TSUP)	5.0×10^−47^	8.5×10^−11^	3.2×10^−28^	3
	9.B.191.1.7 (KDELR)	KXJ91449	XP_010536596	2.A.123.1.27 (Sweet)	1.7×10^−128^	5.3×10^−10^	5.6×10^−56^	7
	2.A.105.1.1 (MPC)	XP_008085704	BAJ94651	2.A.123.1.13 (Sweet)	2.1×10^−29^	4.7×10^−10^	1.1×10^−71^	3
	2.A.104.1.1 (AlaE)	WP_039030005	WP_087291645	2.A.119.2.2 (ArsP)	4.4×10^−69^	1.8×10^−8^	4.9×10^−61^	4
	2.A.129.1.5 (LST)	WP_020576852	WP_068470014	2.A.123.4.1 (Sweet)	8.8×10^−35^	1.5×10^−8^	6.3×10^−21^	4
**Core members**	2.A.43.3.2 (LCT)	XP_005085010	WP_088318471	2.A.123.3.3 (Sweet)	1.1×10^−85^	2.8×10^−10^	1.7×10^−11^	7
	2.A.43.2.1 (LCT)	XP_010897069	AJQ93431	2.A.102.4.13 (TSUP)	5.9×10^−86^	3.4×10^−10^	8.6×10^−30^	4
	2.A.43.2.3 (LCT)	XP_021870097	PAA59477	2.A.82.1.1 (OST)	5.4×10^−49^	6.3×10^−7^	5.6×10^−26^	4
	2.A.52.1.1 (NiCoT)	WP_060363333	OXS28752	2.A.102.4.7 (TSUP)	6.1×10^−64^	1.8×10^−8^	1.4×10^−17^	6
	3.E.1.1.2 (MR)	WP_047004083	XP_022019458	2.A.123.1.23 (Sweet)	1.9×10^−24^	2.9×10^−10^	3.2×10^−39^	5
	3.E.3.1.7 (HelioR)	KKP65948	SAM69932	3.E.1.4.3 (MR)	1.1×10^−68^	9.4×10^−10^	2.0×10^−41^	5
	3.E.1.4.2 (MR)	AHH02121	XP_009982643	9.A.14.13.7 (GPCR)	2.6×10^−13^	9.0×10^−7^	9.5×10^−35^	5

All comparisons were congruent with the alignment of hydrophobic peaks, the agreement of repeat units and Pfam domain content. E-values are shown for the alignments A-B, B-C, and C-D across the homology transitivity paths. B-C E-values relating families A and D are shaded.

^**†**^ Number of hydrophobic peaks (putative TMSs) involved in the B-C alignment (Aln).

**Fig 12:**
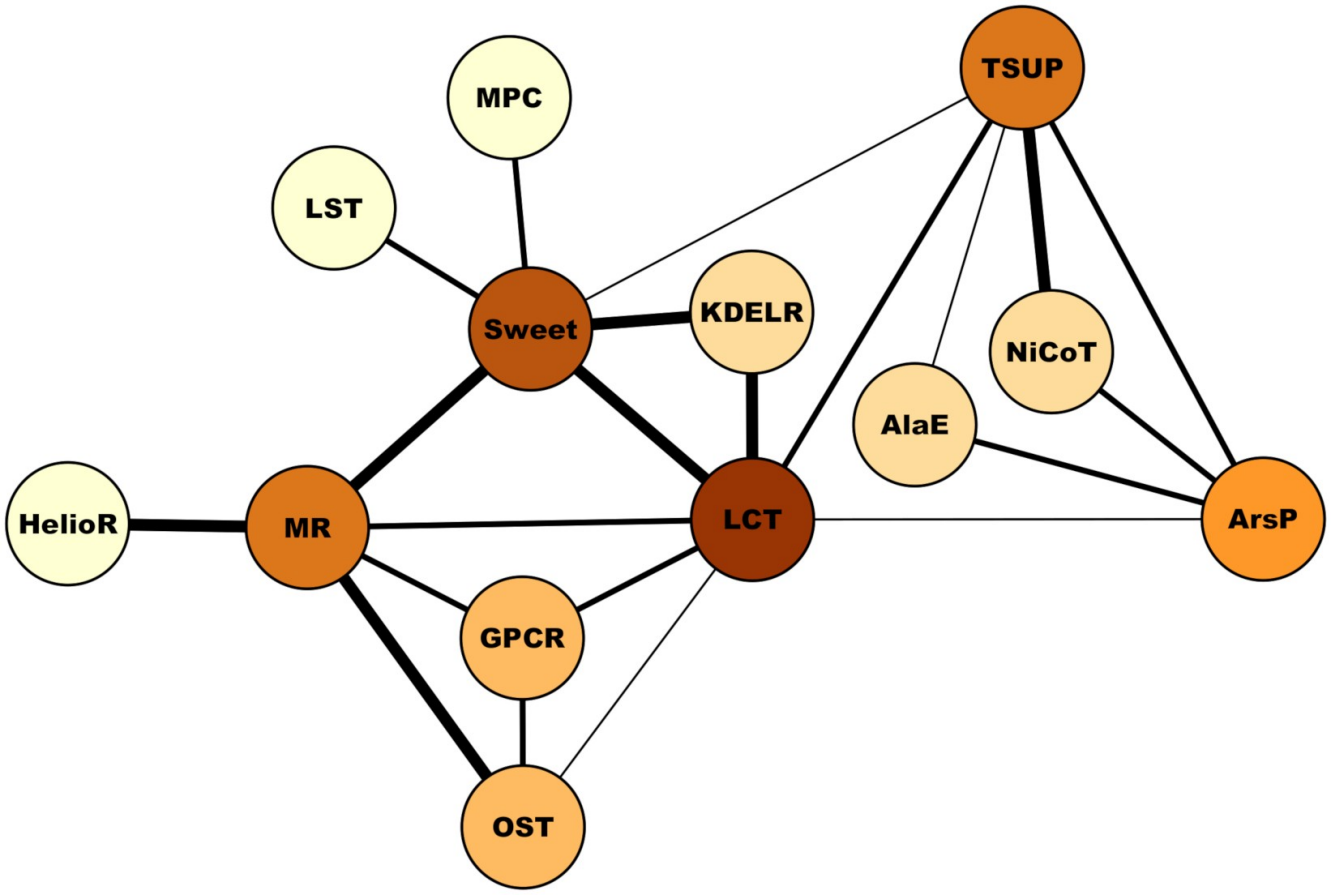
Network of relationships within the TOG superfamily. Nodes represent all families in TOG used in this study. The darker the color of a node the more connections that family has to other families. The relative confidence level (i.e., high, medium or low) of the homology assignment between two families is expressed with three levels of thickness of the edges connecting pairs of nodes; the thickest lines corresponding to the connections of highest confidence. See Methods and S3 Table for how the confidences levels where defined.

Given that all of our homology inferences show Pfam domain overlap and TMS topologies compatible with the repeat units of the families involved, the relative confidence in our homology inferences can be expressed with a score that increases proportionally to the number of TMSs in the alignments and is inversely related to the magnitude of the E-value. We used this score to define 3 arbitrary levels of confidence: high, medium and low (S3 Table; see Methods). Fig 12 shows the network of relationships identified within TOG, where the level of shading in the nodes reflects the degree of connectivity (darker nodes have more connections) and the thicknesses of the edges connecting nodes indicates our relative confidence (i.e., high, medium or low) in the inference of homology between each pair of families (see Methods).

## Discussion

Our methodology (Fig 1) has allowed us to incorporate the families ArsP (TC: 2.A.119), KDELR (TC: 9.B.191), MPC (TC: 2.A.105), AlaE (TC: 2.A.104), and LST (TC: 2.A.129) into the TOG Superfamily [8] (Table 3). The repeat units of these families are compatible with the basic 4-TMS repeat unit that characterizes the TOG superfamily. These proteins sometimes have a single repeat unit of 4 TMSs, although the first TMS is often lost, yielding 3-TMS proteins (e.g., Fig 7 and S11D–S11F Fig), or they exhibit two such units that resulted from an intragenic duplication event (e.g., Fig 3 and S10 Fig), but such an 8-TMS protein may similarly have lost an N-terminal TMS, yielding a 7-TMS protein (e.g., S2, S3, S6 and S9 Figs). In some cases, there is additional gain of TMSs at the N-terminus (e.g., S1A Fig), center (e.g., S1B Fig), or C-terminus (e.g., S4A Fig). Families Sweet, KDELR, MPC, AlaE and LST contain members that appear to have only a single 4-TMS element, lacking a duplicate of itself. However, families MPC and Sweet also contain members with 3 TMSs after loss of the N-terminal TMS (e.g., Fig 7 and S11D–S11F Fig). All of these families also include the larger proteins with 8 or 7 TMSs.

The five new families (Table 2), now identified as TOG superfamily members, substantially expand the transport capabilities of this superfamily. The ArsP, MPC, AlaE, and LST families, respectively, transport organo-arsenical compounds in prokaryotes, pyruvate in mitochondria in a wide range of eukaryotic phyla, alanine in prokaryotes, and lipid-linked sugars (glycosides) in bacteria. All of the members of these 4 families are believed to be secondary carriers, but the members of one of these families (MPC) only catalyze uptake (cation symport), while the others (AlaE, ArsP and LST) catalyze export (cation antiport). None of the previously established members of the TOG superfamily acted upon these substrates.

Most surprising was the finding that the KDELR family belongs to the TOG superfamily, given that a molecular transport function for the KDEL receptors has not been demonstrated, but the significant structural similarity with the Sweet family confirmed its membership to TOG [33]. These receptors normally have 7 or 8 TMSs, but some homologs are half sized, with 4 TMSs (e.g., OWB66932 and XP_013893049) and only one repeat unit, as is characteristic of some other TOG superfamily members. It was named because it recognizes proteins with a Lys-Asp-Glu-Leu (KDEL) sequence [51]. Its originally recognized function was to retrotransport chaperones from the Golgi complex to the Endoplasmic Reticulum (ER), although we now know that this is only one of its several functions. For example, it is involved in protein trafficking involving other intracellular compartments and the plasma membrane, and it triggers the activation of Src family kinases within the Golgi [52]. It is also involved in moving cargo from the ER to the Golgi and in maintaining homeostasis of the entire intracellular protein trafficking apparatus [14]. It functions, therefore, in protein quality control in the cellular protein secretory pathway, probably in all eukaryotes, and it helps to mediate adaptation to multiple types of ER stress [51]. Moreover, it has been shown in mice that the KDEL receptor plays a role in neuronal development and age-related neurodegeneration [51].

As mentioned in the introduction, the TOG superfamily was previously known to include several families of secondary carriers, light-driven primary active transporters as well as photoreceptors and photon-activated ion channels (TCs: 3.E.1 and 3.E.3), and G-protein coupled receptors (GPCRs; TC: 9.A.14). Several GPCRs, in addition to their receptor functions, can sometimes catalyze ion transport, probably by a channel mechanism, as well as transmembrane lipid flipping [53–56]. The KDELR family represents the third family in the TOG superfamily known to include members that mediate a variety of receptor functions. Whether some or all of these KDELR family receptors also catalyze ion movement or lipid flipping has yet to be determined [57–59].

Expansion of the TOG superfamily, as suggested here, and the methodologies we have developed to detect distant homologous relationships will be useful to many investigators. In addition to the types of analyses described, these methodologies can be used to identify potential templates from remote homologs with available structural information to aid in predicting and solving 3D protein structures. For example, the recent definition and characterization of the Anoctamin superfamily (TC: 1.A.17) established the relationships and topological similarities among the ANO (TC: 1.A.17.1), CSC (TC: 1.A.17.5), and 5 other families [1]. Additional evidence supporting the structure of the superfamily has been recently reported [60, 61]. These findings were useful for solving the structure of the CSC member OSCA1.2 from *Oryza sativa* [62] and *Arabidopsis thaliana* [63], which is distantly related to the fungal homolog TMEM16 (TC: 1.A.17.1.18), an ANO family member with reported 3D structure [64]. Furthermore, after the original submission of this manuscript for publication, a 3D structure of a KDEL member (PDB: 6I6B; TC: 9.B.191.1.8) became available. The authors reported significant structural similarity (RMSD: 2.57–3.87 Å) with a Sweet member (TC: 2.A.123.1.18; PDB: 5CTG). This alignment is congruent with the repeat unit of both families [33], confirming the power of our methodology. Thus, we are confident that these strategies will prove valuable inside and outside of the transport biology scientific community.

## Methods

All programs developed for this project can be downloaded directly from our GitHub repository (https://github.com/SaierLaboratory).

### Homology detection between families

We searched for homology between pairs of families using our program areFamiliesHomologous [1]. This program integrates into a pipeline several of our previously reported methods, including Protocol2 and GSAT [1,32]. We performed the initial screening of candidate homologous proteins against the NCBI non-redundant databased using a BLAST E-value < 10^−4^ [65] as the cutoff value and an alignment coverage of at least 40% of the shorter sequence. Highly similar hits were removed using CD-HIT [66] with an identity threshold of 90%. Protocol2 detects similarities between pairs of lists of putative homologues based on the Smith-Waterman algorithm as implemented in SSEARCH [27] using 1000 random shuffles to correct for compositional bias. Based on HMMTOP [29] predictions, at least 3 TMS (the minimal size of the repeat unit in TOG) were required to be included in the alignments, and only hits with E-value < 10^−5^ throughout the transitivity path were further considered. Alignment quality was verified with our program HVORDAN, which generates hydropathy plots for each protein in the transitivity path using the program QUOD to delimit the regions involved in each alignment as well as the location of Pfam domains [67]. QUOD is based on the program WHAT [32] but extends its capabilities and gives the user much more control (e.g., plots sequence information content, places markers indicating the locations of motifs and domains, delimits regions with bars and wedges, customizes colors, and more). Candidate homologs are identified after inspection of the plots generated by HVORDAN, where hydropathy curves of aligned regions show reasonable superimposition of hydrophobic peaks, and there is a Pfam domain overlap of at least 3 TMSs (or the full Pfam domain if smaller than 3 TMSs) within the alignment. The justification is three-fold: 1) often, Pfam domains cover 7 or more TMS, and as shown in Table 1, these originated from a 4-TMS precursor that underwent genetic duplication followed by loss or gain of TMSs; 2) the topology of proteins with 7 TMSs consists of two repeats of 3 TMSs separated by 1 TMS, and 3) there is structural data supporting the 3+1+3 topology in TOG families [68, 69].

### Selection of a negative control set for homology detection

For the negative control (NC) set we selected 10 families for which no evidence of homology with any family in TOG has been reported in the literature. The application of multiple methods failed to identify relationships between TOG and the NC. Currently, families in the NC do not belong to any existing superfamily in TCDB, and their members contain at least 3 TMSs (S1 Table), but we did not investigate potential relationships within the NC. We consider these families a good reference set to study the behavior of family comparisons when no homology can be detected by current methods. All of these families failed the second criterion in of our methodology when compared to families in the TOG superfamily, that is, they did not show compatibility of topology and TMS repeat units with families in the TOG superfamily.

### Identification of repeat units in transporters

Initial searches for strong repeat signals within single proteins were carried out with our program tmsRepeat. This program cuts a query transporter sequence into TMS bundles of predefined size (based on HMMTOP predictions), and non-overlapping bundles are aligned using SSEARCH [27]. For each protein, the program reports the E-value, percentages of identity, similarity, coverage of aligned bundles, and it generates hydropathy plots using QUOD to highlight the regions involved in the putative repeats.

When clear repeat signals could not be detected with tmsRepeat, we retrieved all available candidate homologs from NCBI (BLAST E-value < 10^−10^ and coverage ≥ 60% of the smaller protein) with the same TMS topology as the query protein. Multiple alignments were generated with MAFFT [70] using the L-INS-i algorithm and then edited with trimAL [71] to keep positions with less than 30% gaps. The resulting multiple alignments were then used to search for sequence repeats with the programs AncientRep [32] and HHrepID [72, 73]. For AncientRep, the specific positions where the multiple alignment would be sectioned to guide the search of repeat sequences, were identified by plotting the average hydropathy of the multiple alignment using AveHAS [28].

### Projection of Pfam domains

Query sequences were compared against Pfam [67] with the program hmmscan from the HMMer suite of programs [74] using a gathering threshold. If a protein did not have a direct match with the most common domain observed within its family (present in at least 50% of family members), we attempted to project the domains of the family members with direct Pfam hits onto the sequences of members without hits [1]. That is, we collected the sequence regions with direct Pfam hits in the family and aligned them to the proteins with no hits using SSEARCH [27]. If significant alignments were detected (E-value < 10^−5^ and coverage ≥ 40% of the domain sequences to account for sequences with two repeat units), then the domain was considered to be present in the query protein. This process is implemented in our program getDomainTopology.

### Sequence profile identification across the TOG superfamily

From the set of homologs extracted for each family as described in section “Homology detection between families”, we selected those showing E-value < 10^−15^ and coverage > 70% of the smaller protein. To form the training set for motif identification with MEME [47], we selected from TCDB, 50 proteins from each family in the TOG superfamily (including the new additions). If a family had fewer than 50 proteins in TCDB, the remaining proteins were taken from the corresponding group of extracted homologs, and sequences showing more than 80% identity were removed with CD-HIT [66]. This produced a final training set of 650 proteins for the 13 TOG families. Then, two test sets were generated by combining 1) all homologs extracted, excluding those added to the training set, for the 13 TOG families (16,771 proteins), and 2) all homologs extracted for the families in the negative control (7,321 proteins).

MEME was run in two modes: 1) every sequence in the training set contributes one site (OOPS), and 2) each sequence could contribute zero or one site (ZOOPS). We searched for the top 10 motifs with a width of 15–50 residues that showed an E-value < 10^−50^, convergence distance ≤ 10^−7^, and using the numerically correct (nc) object function to select the best motif in each of the, at most, 1000 iterations of the Expectation Maximization algorithm. We used MAST to scan for motif matches with an E-value < 10^−3^ and p-value < 10^−3^, but we focused our discussion on matches with p-value < 10^−6^. S1 File contains the sequences and MEME/MAST results used in this study.

### Clustering of family members within the TOG superfamily

We extracted 361sequences available in TCDB for all families included in this report and selected a representative set of 306 proteins that maximizes the among-family divergences while preserving the relative sizes of the families. Sequences were clustered with our program mkProteinClusters [1], which uses the statistical computing environment R (https://www.R-project.org/) to perform a hierarchical clustering; based on a distance matrix calculated from bit scores generated by local Smith-Waterman alignments as implemented in SSEARCH [27]. In a previous study, this method has shown excellent agreement with phylogenetic trees for grouping TCDB families [1]. Clusters were generated using the Average agglomerative method. The printed version of the tree (Fig 11) was generated with the GNU software GIMP 2.10 (https://www.gimp.org/). S2 File contains the sequences used to generate the tree. The original tree file in Nexus format is available in S3 File.

### Network of relationships within TOG and their relative confidence levels

Due to the lack of 3D structures in the new families being incorporated into TOG, our inferences relied primarily on four of the five criteria specified in Fig 1. To quantify the relative degrees of confidence in a given inference, we rationalized that the most important factors are the E-value of the B-C alignment between a pair of families and the number of TMSs involved in the alignment, with the proviso that all alignments agree with the repeat units of the respective families, and that there is overlap of the relevant Pfam domains. The contributions of these two factors can be written as

$$Score=-N{log}_{10}(Evalue),$$

where *N* is the number of TMSs in the alignment. The underlying assumption is that the greater the number of TMSs in the alignment and the smaller the E-value, the more reliable the inferences are. The score was normalized to 1.0 based on the highest scoring inference, and three arbitrary levels of confidence were defined: high confidence (Score ≥ 0.6), medium confidence (0.6 > Score ≥ 0.4), and low (Score < 0.4). S3 Table provides the Score and the Confidence level assigned to each inferred relationship within TOG. All the relationships identified within TOG and their confidence levels were plotted in a network layout (Fig 12) using the program Gephi 0.9.2 [75] (https://gephi.org/).

### 3D structural analyses

When full protein 3D structures do not align properly, evidence of homology can still be detected by cutting structures of transporters into bundles of 3 or more transmembrane α-helices (α-TMSs) based upon to size of the repeat units in TOG families. We extracted α-TMSs from OPM [76] as well as PDBTM [77], and if they corresponded to less than one full α-helix, they were extended to full helices using secondary structure assignments from STRIDE [78]. The purpose is to identify significant structural superpositions of the helix bundles corresponding to the repeat units of two families of transporters. Structures are compared with the CCP4 [79] implementation of the SSM superpose algorithm [80] or the TM-align program [81]. Alignments are ranked based on RMSD values, coverage, and TM-scores. Our program Deuterocol automates all these steps. The researcher must make the final decision after inspection and interpretation of top-scoring alignments. Future versions of this program will implement the step of interpretation of the alignments.

## Supporting information

S1 Table General properties of families within the negative control set. (DOCX)

S2 Table Comparison of five new families in TOG with families in the negative control set. (DOCX)

S3 Table Relative confidence scores of homology inferences. (DOCX)

S1 Fig Basic 4-TMS repeat unit in family ArsP. A representative alignment between proteins PIU02666 and WP_094226599, illustrating the 4-TMS repeat unit in ArsP as identified by AncientRep [32] (see Methods). Thin black vertical lines with wedges delimit the regions within full proteins involved in the alignment. Hydrophobic peaks, corresponding to inferred TMSs, are highlighted with orange and cyan vertical bars. A. Hydropathy plot of protein PIU02666. TMSs 2–5 (shaded in dark gray) participate in the alignment shown in panel D. Notice that this protein has an extra N-terminal TMS (not highlighted in orange) that is evidenced by its exclusion from the alignment shown in panel D and by the TMSs covered by the Pfam domain (PF03773). B. Hydropathy plot of protein WP_094226599. Hydrophobic peaks 7–10 (shaded in dark gray) participate in the alignment in panel D. The fifth hydrophobicity peak (not highlighted in cyan) corresponds to 2 TMSs as can be easily determined using alignments with other family homologs that have two clear central hydrophobic peaks (e.g., PIN83468, WP_091710383, etc.). C. Hydropathy of the full protein alignment (E-value: 3.2×10^−42^). Notice how the 2 central TMSs of protein WP_094226599 are mostly aligned with gaps (interruptions in the red curve). D. Hydropathy of the 4-TMS alignment (E-value: 7.8×10^−15^) that provides evidence for the repeat. Interruptions in the hydropathy curves of panels C and D indicate gaps in the corresponding sequence alignments.
(TIF)

S2 Fig Possible o rigin of the 3+1+3 topology in ArsP. A. Hydropathy of 7-TMS ArsP homolog KIL52798. B. Hydropathy of 8-TMS ArsP member WP_069955515 (TC: 2.A.119.1.5). C. Hydropathy of the alignment (E-value: 6.4×10^−44^) between WP_069955515 (red) and KIL52798 (blue). Interruptions in the hydropathy curves of panel C indicate gaps in the sequence alignment. Thin black vertical lines with wedges in panels A-B delimit the region of these proteins involved in the alignment presented in panel C. The loss of the N-terminal TMS in homolog KIL52798, rather than the addition of a TMS in WP_069955515, is evident because 1) it is not part of the alignment; and 2) the Pfam domain PF03773 includes the first TMS.
(TIF)

S3 Fig Possible origin of the 3+1+3 topology in LCT. A. Hydropathy plot of 7-TMS LCT homolog OAD01438. B. Hydropathy plot of 8-TMS LCT homolog XP_011392522. C. Hydropathy plot of the alignment (E-value: 1.0×10^−49^) between OAD01438 (red) and XP_011392522 (blue). Interruptions in the hydropathy curves of panel C indicate gaps in the sequence alignment. Thin black vertical lines with wedges in panels A-B delimit the regions of these proteins involved in the alignment presented in panel C. There are two pieces of evidence supporting the loss of the N-terminal TMS in homolog KIL52798, and thus a 3+4 topology in LCT members with 7 TMSs: 1) the first TMS is not part of the alignment; and 2) The similarity of the hydropathy curve between the first and second 4-TMS halves is evident (Panel B).
(TIF)

S4 Fig Evidence of homology between families ArsP and TSUP. Hydropathy plots are presented across the homology transitivity path between families ArsP and TSUP. Refer to the legend of Fig 4 for a detailed description of the format. A. Hydropathy plot of ArsP member Q8EJL9 (TC: 2.A.119.1.6). B. Hydropathy plot of the alignment (E-value: 5.0×10^−47^) between ArsP member Q8EJL9 and its homologue WP_082464241. C. Hydropathy plot of ArsP homolog WP_082464241. Note that both proteins Q8EJL9 and WP_082464241 share two properties: 1) the third hydrophobic peak is composed of two TMSs; and 2) there is an extra (not colored) C-terminal hydrophobic peak. Both properties can be easily observed from alignments with other ArsP members, for example WP_069955515 (TC: 2.A.119.1.5), and by the regions covered by the Pfam domain PF03773. D. Hydropathy of TSUP member Q9UYH7 (TC: 2.A.102.4.1). E. Hydropathy of the alignment (E-value: 3.2×10^−28^) between TSUP member Q9UYH7 and its homologue AHF91483. F. Hydropathy of TSUP homolog AHF91483. G. Hydropathy of the 3-TMS alignment (E-value: 8.5×10^−11^) between ArsP homologue WP_082464241and TSUP homologue AHF91483. Only the regions where hydrophobic peaks overlap are highlighted in the alignments. The full alignment in panel G is covered by the Pfam domains of both proteins, and the domain (PF03773) in WP_082464241 can be projected to AHF91483 (E-value: 3×10^−6^), further supporting the relationship between the two families.
(TIF)

S5 Fig Evidence of homology between families ArsP and NiCoT. Hydropathy plots are presented across the homology transitivity path between families ArsP and NiCoT. Refer to the legend of Fig 4 for a detailed description of the format. A. Hydropathy plot of ArsP member WP_099137450 (TC: 2.A.119.1.4). B. Hydropathy plot of the alignment (E-value: 1.8×10^−13^) between ArsP member WP_099137450 and its homologue WP_066228546. C. Hydropathy plot of ArsP homolog WP_066228546. Note the extra N-terminal TMS in protein WP_066228546, which can be easily observed both from the region that aligns with member WP_099137450 and the region covered by the Pfam domain PF03773. D. Hydropathy plot of NiCoT member Q7S3L8 (TC: 2.A.52.1.8). E. Hydropathy plot of the alignment (E-value: 6.8×10^−99^) between NiCoT member Q7S3L8 and its homologue PHH64764. F. Hydropathy of NiCoT homolog PHH64764. G. Hydropathy plot of the 4-TMS alignment (E-value: 2.1×10^−8^) between ArsP homologue WP_066228546 and NiCoT homologue PHH64764. Only the regions where hydrophobic peaks overlap are highlighted in the alignments. The alignment in panel G is covered by the Pfam domains of both proteins, and the domain (PF03773) in WP_066228546 can be projected to PHH64764 (E-value: 1.4×10^−5^) further supporting the relationship between these two families.
(TIF)

S6 Fig Possble origin of the 3+1+3 topology in KDELR. A. Hydropathy plot of 7-TMS KDELR homolog XP_003307390. B. Hydropathy plot of 8-TMS KDELR homolog XP_020093034. C. Hydropathy plot of the alignment (E-value: 2.0×10^−22^) between XP_003307390 (red) and XP_020093034 (blue). Interruptions in the hydropathy curves of panel C indicate gaps in the sequence alignment. Thin black vertical lines with wedges in panels A-B delimit the regions of these proteins involved in the alignment presented in panel C. Notice that the alignment contains only hydrophobic peaks 2–8 in XP_020093034. The exclusion of the first hydrophobic peak in XP_020093034 from the alignment provides evidence supporting the loss of the N-terminal TMS in XP_003307390 and other 7-TMS family members.
(TIF)

S7 Fig Evidence of homology between families KDELR and LCT. Hydropathy plots are presented across the homology transitivity path between families KDELR and LCT. Refer to the legend of Fig 4 for a detailed description of the format. A. Hydropathy plot of 7-TMS KDELR member P24390 (TC: 9.B.191.1.5). B. Hydropathy plot of the 7-TMS alignment (E-value: 6.0×10^−20^) between KDELR member P24390 and its homologue PIA50795. C. Hydropathy plot of KDELR homolog PIA50795. Note that the alignment starts in the second hydrophobic peak of homolog PIA50795, which further supports the loss of the N-terminal TMS from KDELR homologs with 7 TMSs. D. Hydropathy plot of LCT member Q60441 (TC: 2.A.43.3.1). E. Hydropathy plot of the alignment (E-value: 7.9×10^−31^) between LCT member Q60441 and its homologue KXN87232. F. Hydropathy of LCT homolog KXN87232. G. Hydropathy plot of the 5-TMS alignment (E-value: 3.1×10^−9^) between KDELR homolog PIA50795 and LCT homolog KXN87232. Only the regions where hydrophobic peaks overlap are highlighted in the alignments. The alignment in panel G includes most of the Pfam domains of both proteins. In addition, KDELR domain (PF00810) is directly found in LCT homologue KXN87232 (hmmscan E-value: 5.3×10^−5^) without the need of projection, further supporting the relationship between both families.
(TIF)

S8 Fig Repeat unit in family PnuC. A representative alignment between 8-TMS proteins WP_027672312 and OWU65212 illustrates the 4-TMS repeat unit in PnuC as identified by AncientRep [32]. Thin vertical black lines with wedges delimit the regions involved in the alignment of the two full-length proteins. Orange and cyan bars highlight hydrophobicity peaks (i.e., inferred TMSs), respectively, for both proteins. A. Hydropathy plot of protein WP_027672312. TMSs 1–4 (shaded in dark gray) participate in the alignment shown in panel D. B. Hydropathy plot of protein OWU65212. Hydrophobic peaks 5–8 (shaded in dark gray) participate in the alignment shown in panel D. C. Hydropathy plot of the alignment (E-value: 4.3×10^−14^) between the full proteins. D. Hydropathy plot of the 4-TMS alignment (E-value: 3.7×10^−7^) that provides evidence for the repeat. The good overlap of the hydropathy curves increases the significance of the alignment. Interruptions in the hydropathy curves of panels C and D indicate gaps in the corresponding sequence alignments.
(TIF)

S9 Fig Posible origin of the 3+1+3 topology in PnuC. Representative alignment supporting the loss of the N-terminal TMS in PnuC members with 7 TMSs. A. Hydropathy of 7-TMS PnuC homolog OFX33391. B. Hydropathy of 8-TMS PnuC homolog WP_092670402. C. Hydropathy of the alignment (E-value: 3.0×10^−23^) between OFX33391 (red) and WP_092670402 (blue). Interruptions in the hydropathy curves of panel C indicate gaps in the sequence alignment. Thin black vertical lines with wedges in panels A and B delimit the regions of these proteins involved in the alignment presented in panel C. The loss of the N-terminal TMS in homolog OFX33391 is supported by the fact that the first TMS in WP_092670402 (panel B) is not part of the alignment shown in Panel C.
(TIF)

S10 Fig Repeat unit of family sweet. The repeat unit of Sweet members with 7 TMS is 3+1+3 where TMSs 1–3 are highly similary to TMSs 5–7. Here we present a representative example of a Sweet homolog with 8 TMSs showing a 4-TMS repeat as identified by our program tmsRepeat (see Methods). A. Hydropathy plot of the 8-TMS Sweet homolog PFH37642. Thin black vertical lines with wedges delimit the two 4-TMSs bundles that were initially aligned. The regions within the 4-TMS bundles that were aligned by SSEARCH [27] (see Methods) are shaded blue and green, respectively. B. Hydropathy plot showing the alignment (E-value: 1.7×10^−5^) between TMSs 1–4 (blue) and TMSs 5–8 (green). Interruptions in the hydropathy curves indicate gaps in the sequence alignment.
(TIF)

S11 Fig Loss of the N-terminal TMS in the sweet family. Representative alignments of Sweet homologs supporting the loss of the N-terminal TMS from original proteins with 8 (panels A-C) and 4 TMSs (panels D-F). The thin black bars with wedges in panels A, B, D, and E, delimit the regions of the proteins that participate in the alignments shown in panels C and F. Interruptions in the hydropathy curves of panels C and F indicate gaps in the corresponding sequence alignments. A. Hydropathy plot of 7-TMS Sweet member ANC68268 (TC: 2.A.123.1.27). B. Hydropathy plot of 8-TMS Sweet homolog PFH37642. This is the same protein used in S10 Fig to identify the 4-TMS repeat unit. C. Hydropathy plot of the alignment (E-value: 1.9×10^−19^) between ANC68268 and PFH37642. D. Hydropathy plot of 3-TMS Sweet member C3WG44 (TC: 2.A.123.2.3). E. Hydropathy plot of 4-TMS Sweet homolog WP_082029922. F. Hydropathy plot of the alignment (E-value: 7.1×10^−16^) between ANC68268 and PFH37642. The loss of the N-terminal TMS is supported by the fact that the first TMS in proteins PFH37642 and WP_082029922 is not part of their respective alignments, and that all TMSs in proteins ANC68268 and C3WG44 are aligned.
(TIF)

S12 Fig Insufficient evidence of homology between families PnuC and sweet. Hydropathy plots are presented across the homology transitivity path between families PnuC and Sweet. Refer to the legend of Fig 4 for a detailed description of the format. A. Hydropathy plot of 7-TMS PnuC member Q8EDN0 (TC: 4.B.1.1.4). B. Hydropathy plot of the 7-TMS alignment (E-value: 6.7×10^−22^) between Q8EDN0 and its homologue OFX33391. C. Hydropathy plot of 7-TMS PnuC homolog OFX33391. D. Hydropathy plot of Sweet member H3GD93 (TC: 2.A.123.1.22). This protein consists of the quadruplication of a 7-TMS precursor protein. E. Hydropathy plot of the 7-TMS alignment (E-value: 3.5×10^−52^) between H3GD93 and its homologue OWY93661. Note that OWY93661 has significant alignments (E-value < 10^−30^) with all 4 repeats in H3GD93; however, we present only the top scoring alignment with the fourth repeat as indicated in panel D. F. Hydropathy of the Sweet homolog OWY93661. G. Hydropathy plot of the 4-TMS alignment (E-value: 1.8×10^−8^) between PnuC homolog OFX33391 and Sweet homolog OWY93661. Only the regions where hydrophobic peaks overlap are highlighted in the alignments. The alignment of hydrophobic peaks between OFX33391 (peaks 2–5) and OWY93661 (peaks 3–7) is not consistent with their common topologies (3+1+3 or 3+4), suggesting that the evolution of the TMS architecture in family PnuC followed a different path as compared to families in the TOG superfamily, possibly involving an internal rearrangement of TMSs [35].
(TIF)

S13 Fig Repeat unit of family PNaS. A representative alignment between proteins WP_015713122 and OPL07780 with 10 hydrophobic peaks each, suggests the 4-TMS repeat unit in PNaS as identified by AncientRep [32]. Note that HMMTOP [29] predicts 9 TMSs in both proteins, but comparisons with other family members support the presence of 10 TMSs. Thin black vertical lines and wedges delimit the regions involved in the alignment of the two full-length proteins. Orange and cyan bars highlight hydrophobicity peaks (i.e., inferred TMSs), respectively, for both proteins. A. Hydropathy plot of protein WP_015713122. TMSs 1–4 (shaded in dark gray) participate in the alignment shown in panel D. B. Hydropathy plot of protein OPL07780. TMSs 5–8 (shaded in dark gray) participate in the alignment shown in panel D. C. Hydropathy plot of the alignment (E-value: 1.2×10^−40^) between the full proteins. D. Hydropathy plot of the 4-TMS alignment (E-value: 4.1×10^−12^) that provides evidence for the repeat. Interruptions in the hydropathy curves of panels C and D indicate gaps in the corresponding sequence alignments.
(TIF)

S14 Fig Insufficient evidence of homology between families PNaS and MR. Hydropathy plots are presented across the homology transitivity path between families PNaS and MR. Refer to the legend of Fig 4 for a detailed description of the format. A. Hydropathy plot of PNaS member M7AKZ4 (TC: 2.A.58.2.2). B. Hydropathy plot of the alignment (E-value: 3.6×10^−46^) between M7AKZ4 and its homolog WP_026901115. C. Hydropathy plot of PNaS homolog WP_026901115. D. Hydropathy plot of MR member Q12117 (TC: 3.E.1.4.6). E. Hydropathy plot of the 7-TMS alignment (E-value: 1.2×10^−88^) between Q12117 and its homologue AJP85873. F. Hydropathy of the MR homolog AJP85873. G. Hydropathy plot of the 5-TMS alignment (E-value: 1.7×10^−8^) between PNaS homolog WP_026901115 and MR homolog AJP85873. Only the regions where hydrophobic peaks overlap are highlighted in the alignments. Note that 1) relative to the full protein WP_026901115 (panel C), the alignment in panel G starts in TMS 6, or the second TMS of the second 4-TMS repeat unit, and relative to AJP85873 (panel F) the alignment starts on the third TMS, or TMS 4 of the first 4-TMS repeat unit considering the loss of the N-terminal TMS in family MR [8]; 2) in panel G the second TMS of both proteins show little overlap; and 3) TMSs 9–10 of WP_026901115, which are not part of the 4-TMS repeat unit in PNaS (S13 Fig), are aligning with TMSs 7–8 of AJP85873. Thus, this alignment is not supportive of a common origin.
(TIF)

S15 Fig Repeat unit in family CaTA. A representative alignment between proteins EFQ46215 and WP_005742819 supporting the TMS topology 3+3+1 in agreement with available 3D structural data. Thin black bars with wedges delimit the regions involved in the alignment of the two full-length proteins. Orange and cyan bars highlight hydrophobicity peaks (i.e., inferred TMSs) for each full protein, respectively. A. Hydropathy plot of protein EFQ46215. TMSs 1–3 (shaded in dark gray) participate in the alignment shown in panel D. B. Hydropathy plot of protein WP_005742819. TMSs 4–6 (shaded in dark gray) participate in the alignment shown in panel D. C. Hydropathy plot of the alignment (E-value: 1.8×10^−4^) between the full proteins. D. Hydropathy plot of the 3-TMS alignment (E-value: 5.1×1^−6^) that provides evidence for the repeat. Interruptions in the hydropathy curves of panels C and D indicate gaps in the corresponding sequence alignments.
(TIF)

S16 Fig MAST match mapped to the structure of the eubacterial MR member xanthorhodopsin. Cartoon representation of the 7-TMS xanthorhodopsin structure in *Salinibacter ruber* (PDB: 3DDL). The segment of TMSs 1–2 matched by MAST is shown in red, the retinal cofactor is shown in cyan, and residues making contact with retinal (< 6 Å) within the MAST region are shown in yellow. All other residues are grayed.
(TIF)

S17 Fig MAST match mapped to the structure of the eukaryotic SWEET13 transporter. Cartoon representation of the 7-TMS SWEET13 structure in *Arabidopsis thaliana* (PDB: 5XPD). The segment of TMSs 1–2 matched by MAST is shown in red, the substrate analog, 2’ deoxycytidine 5’ monophosphate, is shown in cyan, and residues making contact with the substrate within the MAST region are shown in yellow. All other residues are grayed.
(TIF)

S18 Fig MAST match mapped to the structure of the eukaryotic KDEL receptor. Cartoon representation of the 7-TMS KDEL receptor structure in *Gallus gallus* (PDB: 6I6H). The segment of TMSs 1–2 matched by MAST is shown in red, the TAEKDEL signal peptide bound to the pocket is shown in cyan, and residues making contact with the peptide within the MAST region are shown in yellow. All other residues are grayed.
(TIF)

S1 File MEME/MAST output files with motifs identified for the TOG superfamily. Folders and ouput files are compressed in a zip file. Folder MEME contains the motif models; folder MAST contains the motifs matching the training sets, and folder DATA contains both the training and test sets. Check the included README file for a detailed description.
(ZIP)

S2 File Sequences used to generate the radial tree of the TOG superfamily. Sequences were renamed to facilitate visualization of the tree. The family name is followed by the 2 last digits of the TC numbers, separated by underscores, to identify the sytems: Family names followed the following convention: SW (Sweet), TS (TSUP), LC (LCT), NI (NiCoT), OS (OST), MR (MR), HE (HelioR), GP (GPCR), AR (ArsP), KD (KDELR), MP (MPC), and LS (LST).
(FAA)

S3 File Tree of the TOG superfamily in nexus format. Individual sequences are named following the same format as S2 File.
(TREE)

## Abbreviations

AlaE: L-Alanine Exporter Family (TC: 2.A.104). New TOG member
ArsP: Organo-Arsenical Exporter Family (TC: 2.A.119). New TOG memer
CatA: Calcium Transporter A Family (TC: 1.A.14). Non-TOG member
GPCR: G-Protein Coupled Receptor Family (TC: 9.A.14). Established TOG member
HelioR: HelioRhodopsin Family (TC: 3.E.3). Established TOG member
KDELR: Endoplasmic Reticulum Retention Receptor Family (TC: 9.B.191). New TOG member
LCT: Lysosomal Cystine Transporter Family (TC: 2.A.43). Established TOG member
LST: Lipid-linked Sugar Translocase Family (TC: 2.A.129). New TOG member
MPC: The Mitochondrial Pyruvate Carrier (TC: 2.A.105). New TOG member
MR: Ion-translocating Microbial Rhodopsin Family (TC: 3.E.1). Established TOG member
NicO: The Nickel/Cobalt Transporter Family (TC: 2.A.113). Non-TOG member
NiCoT: Ni^2+^-Co^2+^ Transporter Family (TC: 2.A.52). Established TOG member
OST: Organic Solute Transporter Family (TC: 2.A.82). Established TOG member
PNaS: Phosphate:Na^+^ Symporter Family (TC: 2.A.58). Non-TOG member
PnuC: Nicotinamide Ribonucleoside Uptake Permease Family (TC: 4.B.1). Non-TOG member
Sweet: Sweet; PQ-loop; Saliva; MtN3 Family (TC: 2.A.123). Established TOG member
TOG: Transporter-Opsin-G protein-coupled receptor Superfamily
TSUP: 4-Toluene Sulfonate Uptake Permease Family (TC: 2.A.102). Established TOG member
