# Correction: Expansion of the Transporter-Opsin-G protein-coupled receptor superfamily with five new protein families

**DOI:** 10.1371/journal.pone.0235297

**Published:** 2020-06-19

**Authors:** The PLOS ONE Staff

The first two authors, Arturo Medrano-Soto and Faezeh Ghazi, should be noted as joint first authors who contributed equally to this work. The publisher apologizes for the error.
