# Supplementary material for: Expansion of the Transporter-Opsin-G protein-coupled receptor superfamily with five new protein families
Source: PLoS One. 2020 Apr 22;15(4):e0231085. doi: 10.1371/journal.pone.0231085 (PMC7176098; doi:10.1371/journal.pone.0231085)
Supplement: S1 Table — (DOCX) [file pone.0231085.s001.docx]

| **Family**  **Name** | **Family abbrv.** | **TCDB accession** | **Average Protein size (**± SD) | **Typical No. of TMSs^†^** | **Organismal distribution** |
| --- | --- | --- | --- | --- | --- |
| Mg^2+^ Transporter-E Family | MgtE | 1.A.26 | 444 ±61**^‡^** | 5, 11 | Bacteria,  Archaea,  Eukaryota |
| Magnesium Transporter1 Family | MagT1 | 1.A.76 | 305 ±67 | 3-5 | Eukaryota |
| Inorganic Phosphate Transporter Family | PiT | 2.A.20 | 437 ±97 | 10-13 | Bacteria  Archaea  Eukaryota |
| Acetate Uptake Transporter Family | AceTr | 2.A.96 | 246 ±43 | 6 | Bacteria  Archaea  Eukaryota |
| Heme Transporter, Heme-Responsive Gene Protein Family | HRG | 2.A.110 | 159 ±19 | 3,4 | Eukaryota |
| LrgB/CidB Holin-like Glycolate/Glycerate Transporter Family | LrgB | 2.A.122 | 234 ±14 | 7-8 | Bacteria  Archaea  Eukaryota |
| The Lysine Exporter Family | LysO | 2.A.124 | 259 ±53 | 5-10 | Bacteria  Archaea |
| Eukaryotic Riboflavin Transporter Family | E-RFT | 2.A.125 | 461 ±36 | 11 | Eukaryota |
| Enterobacterial Cardiolipin Transporter Family | CLT | 2.A.127 | 638 ±40 | 5 | Bacteria |
| Multidrug Resistance Exporter VanZ Family | VanZ | 2.A.128 | 288 ±96**^§^** | 4-5,9 | Bacteria |
| **^†^** The acronym TMS is used synonymously with Hydrophobic Peak.  **^‡^** In family MgtE a substantial number of proteins with 5 TMSs have a long N-terminal hydrophilic domain.  **^§^** Family VanZ has a bimodal distribution of protein lengths; one group has ~200 aas and the other ~375 aas. The difference correlates with the numbers of TMSs, where the latter proteins have about twice as many TMSs. | | | | | |

**S1 Table. General properties of families within the negative control set.**
