# Supplementary material for: Expansion of the Transporter-Opsin-G protein-coupled receptor superfamily with five new protein families
Source: PLoS One. 2020 Apr 22;15(4):e0231085. doi: 10.1371/journal.pone.0231085 (PMC7176098; doi:10.1371/journal.pone.0231085)
Supplement: S2 Table — (DOCX) [file pone.0231085.s002.docx]

**S2 Table. Comparison of five new families in TOG with the families in the negative control set.** For each new family (i.e., ArsP, KDELR, MPC, AlaE, and LST), the table presents the summary data of the alignments across the homology transitivity path. Only E-values are shown for the alignments A-B and C-D because these alignments satisfied all criteria in our methodology (Fig 1), except for structural evidence due to the unavailability of 3D structures in these families. For B-C alignments, only successfully met criteria are shown (shaded green), and the first criterion that fails (shaded red) is explained in the table footnotes, where we refer to hydrophobic peaks as TMSs. For clarity, our second criterion (compatibility of topology and repeat units) is split into similarity of hydropathy profiles and compatibility of repeat units. The abbreviations used to present the results are: E (E-value), H (quality of Hydropathy alignment), R (Repeat unit agreement). Notice that all B-C alignments failed in step 2; they either showed poor hydropathy alignments or the alignment of TMS did not make sense. Interestingly, the top matches versus the negative control set for new families ArsP, KDELR, AlaE, and LST, are observed with the same family, PiT, but none of these alignments passed the second criterion (Fig 1).

| **Homology transitivity path** | | | | **Quality of alignments** | | |
| --- | --- | --- | --- | --- | --- | --- |
| **Family A** | **Homolog B** | **Homolog C** | **Family D** | **A vs B** | **B vs C** | **C vs D** |
| 2.A.119.1.1  (ArsP) | WP_066445430 | PIC34018 | 2.A.20.2.6  (PiT) | 4.1×10^-10^ | E: 2.0×10^-7^ | 4.2×10^-68^ |
|  |  |  |  |  | H: Bad **^1^** |  |
| 9.B.191.1.7  (KDELR) | XP_016626867 | ORZ01047 | 2.A.20.2.10  (PiT) | 1.4×10^-73^ | E: 8.0×10^-8^ | 4.8×10^-79^ |
|  |  |  |  |  | H: Bad **^2^** |  |
| 2.A.105.1.3 (MPC) | BAM20003 | WP_025917480 | 2.A.122.1.4  (LrgB) | 2.3×10^-30^ | E: 2.7×10^-8^ | 1.6×10^-19^ |
|  |  |  |  |  | H: ok |  |
|  |  |  |  |  | R: Bad **^3^** |  |
| 2.A.104.1.1  (AlaE) | WP_062779523 | KFZ12467 | 2.A.20.2.1  (PiT) | 6.3×10^-51^ | E: 2.3×10^-8^ | 1.9×10^-64^ |
|  |  |  |  |  | H: Bad **^4^** |  |
| 2.A.129.1.11  (LST) | WP_003865663 | WP_077463789 | 2.A.20.2.4  (PiT) | 4.1×10^-15^ | E: 4.3×10^-10^ | 2.2×10^-41^ |
|  |  |  |  |  | H: ok |  |
|  |  |  |  |  | R: Bad **^5^** |  |
| **^1^** Alignment covers 5 TMSs in both proteins, but 2 TMS are aligned with hydrophilic regions. In addition, the topology of family PiT is based on repeats of 5 TMSs (unpublished data), which is incompatible with TOG.  **^2^** Alignment covers 5 TMS in XP_016626867 and 4 TMSs in ORZ01047. One TMS is aligned with a hydrophilic region.  **^3^** Alignment covers 3 TMSs in both proteins. BAM20003 is aligning TMSs 1-3 with TMSs 5-7 of WP_025917480. Family MPC derives from a 4-TMS ancestor with loss of the N-terminal TMS (see text). Members of family LrgB commonly have 8 TMSs with topology 4+4 (unpublished data). In this context, BAM20003 is aligning TMS 2-4 of its repeat unit (first TMS was lost), with TMSs 1-3 of the second 4-TMS repeat unit of WP_025917480, which is incompatible with TOG.  **^4^** Short alignment. 2 TMSs are aligned with 1 TMS.  **^5^** Alignment covers 4 TMSs in both proteins. WP_003865663 is aligning TMSs 1-4 with TMSs 4-7 of WP_077463789. This alignment does not make sense because TMSs 6-7 of WP_077463789 are not part of the repeat unit and because the PiT family has a 5-TMS repeat unit. | | | | | | |
