# Supplementary material for: Expansion of the Transporter-Opsin-G protein-coupled receptor superfamily with five new protein families
Source: PLoS One. 2020 Apr 22;15(4):e0231085. doi: 10.1371/journal.pone.0231085 (PMC7176098; doi:10.1371/journal.pone.0231085)
Supplement: S3 Table — (DOCX) [file pone.0231085.s003.docx]

**S3 Table. Relative confidence scores of homology inferences.**

| Family pairs | |  |  |  |  |
| --- | --- | --- | --- | --- | --- |
| Family 1 | Family 2 | E-value | Aligned TMSs | Score | Confidence |
| AlaE | ArsP | 1.8×10^-8^ | 4 | 0.46 | Medium |
| AlaE | TSUP | 6.8×10^-7^ | 4 | 0.37 | Low |
| ArsP | LCT | 1.9×10^-9^ | 3 | 0.39 | Low |
| ArsP | NiCoT | 2.1×10^-8^ | 4 | 0.46 | Medium |
| ArsP | TSUP | 8.5×10^-11^ | 3 | 0.45 | Medium |
| GPCR | LCT | 4.5×10^-7^ | 6 | 0.57 | Medium |
| GPCR | MR | 9.0×10^-7^ | 5 | 0.45 | Medium |
| GPCR | OST | 2.4×10^-8^ | 4 | 0.46 | Medium |
| HelioR | MR | 9.4×10^-10^ | 5 | 0.67 | High |
| KDELR | LCT | 3.1×10^-9^ | 5 | 0.64 | High |
| KDELR | Sweet | 5.3×10^-10^ | 7 | 0.97 | High |
| LCT | MR | 3.6×10^-8^ | 4 | 0.45 | Medium |
| LCT | OST | 6.3×10^-7^ | 4 | 0.37 | Low |
| LCT | Sweet | 2.8×10^-10^ | 7 | 1.00 | High |
| LCT | TSUP | 3.4×10^-10^ | 4 | 0.57 | Medium |
| LST | Sweet | 1.5×10^-8^ | 4 | 0.47 | Medium |
| MPC | Sweet | 4.7×10^-10^ | 3 | 0.42 | Medium |
| MR | OST | 6.0×10^-7^ | 7 | 0.65 | High |
| MR | Sweet | 2.9×10^-10^ | 5 | 0.71 | High |
| NiCoT | TSUP | 1.8×10^-8^ | 6 | 0.69 | High |
| Sweet | TSUP | 4.6×10^-8^ | 3 | 0.33 | Low |
