# Supplementary figures and images for: Expansion of the Transporter-Opsin-G protein-coupled receptor superfamily with five new protein families
Source: PLoS One. 2020 Apr 22;15(4):e0231085. doi: 10.1371/journal.pone.0231085 (PMC7176098; doi:10.1371/journal.pone.0231085)

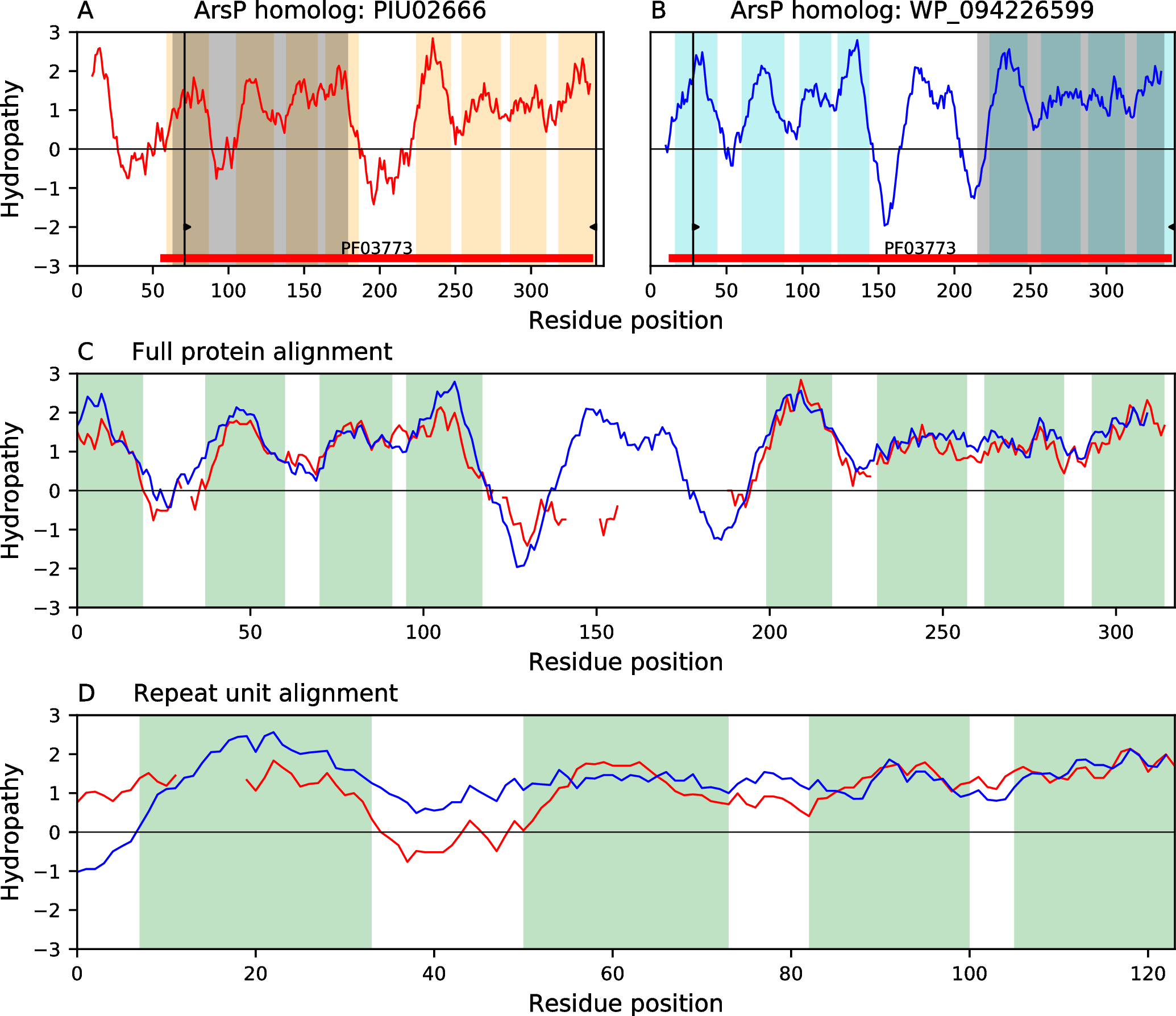

Supplement: S1 Fig — A representative alignment between proteins PIU02666 and WP_094226599, illustrating the 4-TMS repeat unit in ArsP as identified by AncientRep [32] (see Methods). Thin black vertical lines with wedges delimit the regions within full proteins involved in the alignment. Hydrophobic peaks, corresponding to inferred TMSs, are highlighted with orange and cyan vertical bars. A. Hydropathy plot of protein PIU02666. TMSs 2–5 (shaded in dark gray) participate in the alignment shown in panel D. Notice that this protein has an extra N-terminal TMS (not highlighted in orange) that is evidenced by its exclusion from the alignment shown in panel D and by the TMSs covered by the Pfam domain (PF03773). B. Hydropathy plot of protein WP_094226599. Hydrophobic peaks 7–10 (shaded in dark gray) participate in the alignment in panel D. The fifth hydrophobicity peak (not highlighted in cyan) corresponds to 2 TMSs as can be easily determined using alignments with other family homologs that have two clear central hydrophobic peaks (e.g., PIN83468, WP_091710383, etc.). C. Hydropathy of the full protein alignment (E-value: 3.2×10^−42^). Notice how the 2 central TMSs of protein WP_094226599 are mostly aligned with gaps (interruptions in the red curve). D. Hydropathy of the 4-TMS alignment (E-value: 7.8×10^−15^) that provides evidence for the repeat. Interruptions in the hydropathy curves of panels C and D indicate gaps in the corresponding sequence alignments. (TIF) [file pone.0231085.s004.tif]

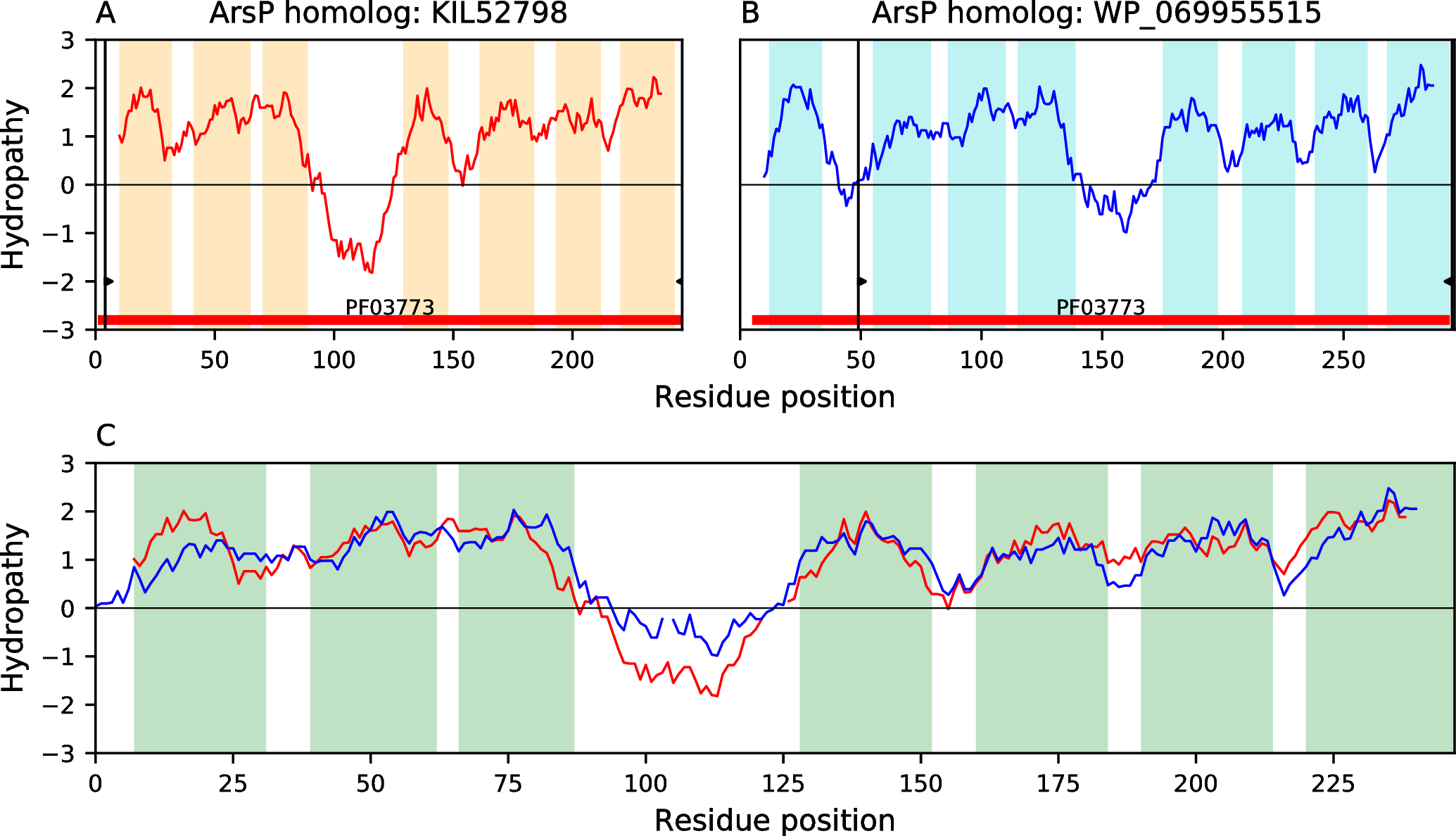

Supplement: S2 Fig — A. Hydropathy of 7-TMS ArsP homolog KIL52798. B. Hydropathy of 8-TMS ArsP member WP_069955515 (TC: 2.A.119.1.5). C. Hydropathy of the alignment (E-value: 6.4×10^−44^) between WP_069955515 (red) and KIL52798 (blue). Interruptions in the hydropathy curves of panel C indicate gaps in the sequence alignment. Thin black vertical lines with wedges in panels A-B delimit the region of these proteins involved in the alignment presented in panel C. The loss of the N-terminal TMS in homolog KIL52798, rather than the addition of a TMS in WP_069955515, is evident because 1) it is not part of the alignment; and 2) the Pfam domain PF03773 includes the first TMS. (TIF) [file pone.0231085.s005.tif]

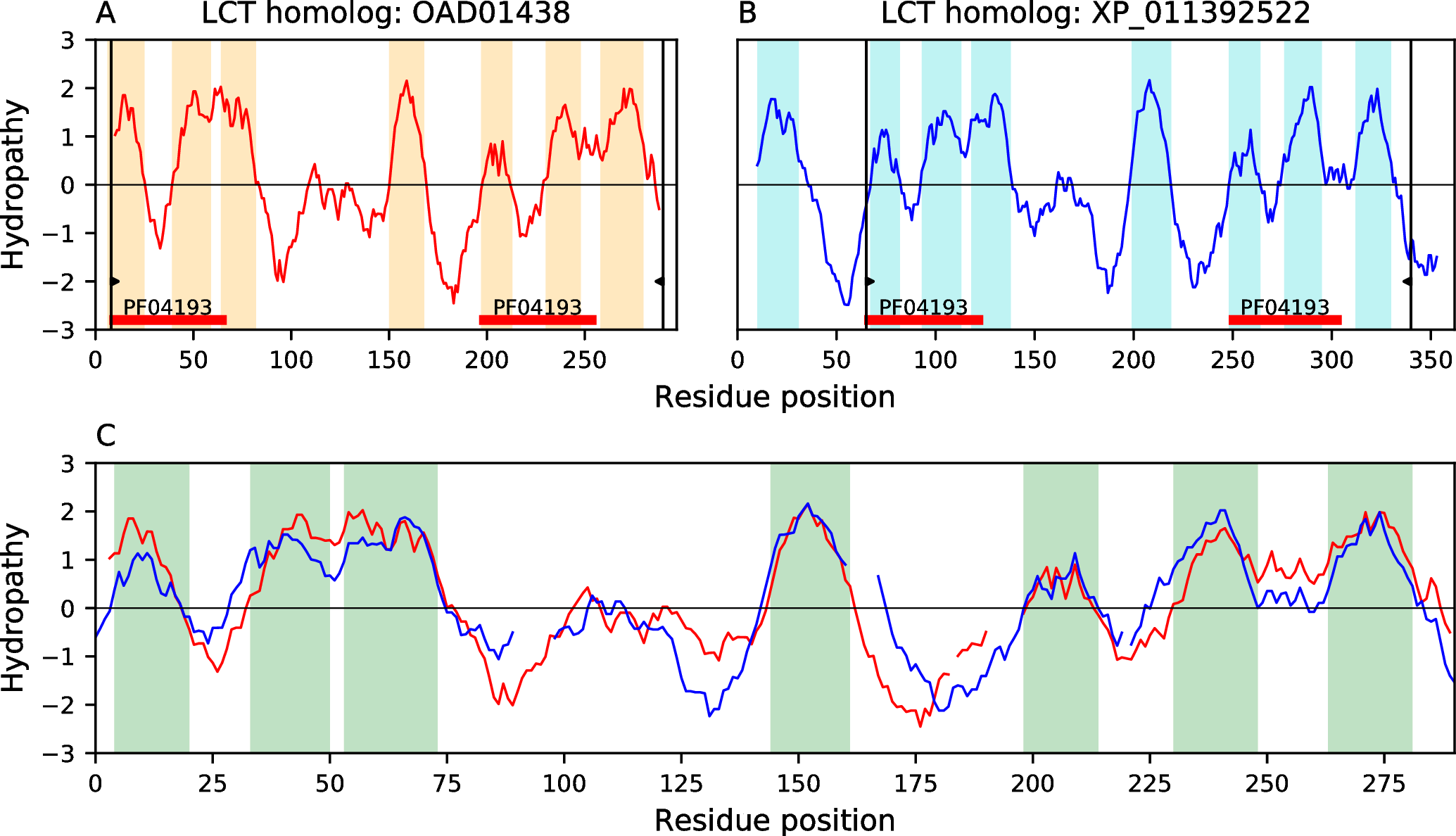

Supplement: S3 Fig — A. Hydropathy plot of 7-TMS LCT homolog OAD01438. B. Hydropathy plot of 8-TMS LCT homolog XP_011392522. C. Hydropathy plot of the alignment (E-value: 1.0×10^−49^) between OAD01438 (red) and XP_011392522 (blue). Interruptions in the hydropathy curves of panel C indicate gaps in the sequence alignment. Thin black vertical lines with wedges in panels A-B delimit the regions of these proteins involved in the alignment presented in panel C. There are two pieces of evidence supporting the loss of the N-terminal TMS in homolog KIL52798, and thus a 3+4 topology in LCT members with 7 TMSs: 1) the first TMS is not part of the alignment; and 2) The similarity of the hydropathy curve between the first and second 4-TMS halves is evident (Panel B). (TIF) [file pone.0231085.s006.tif]

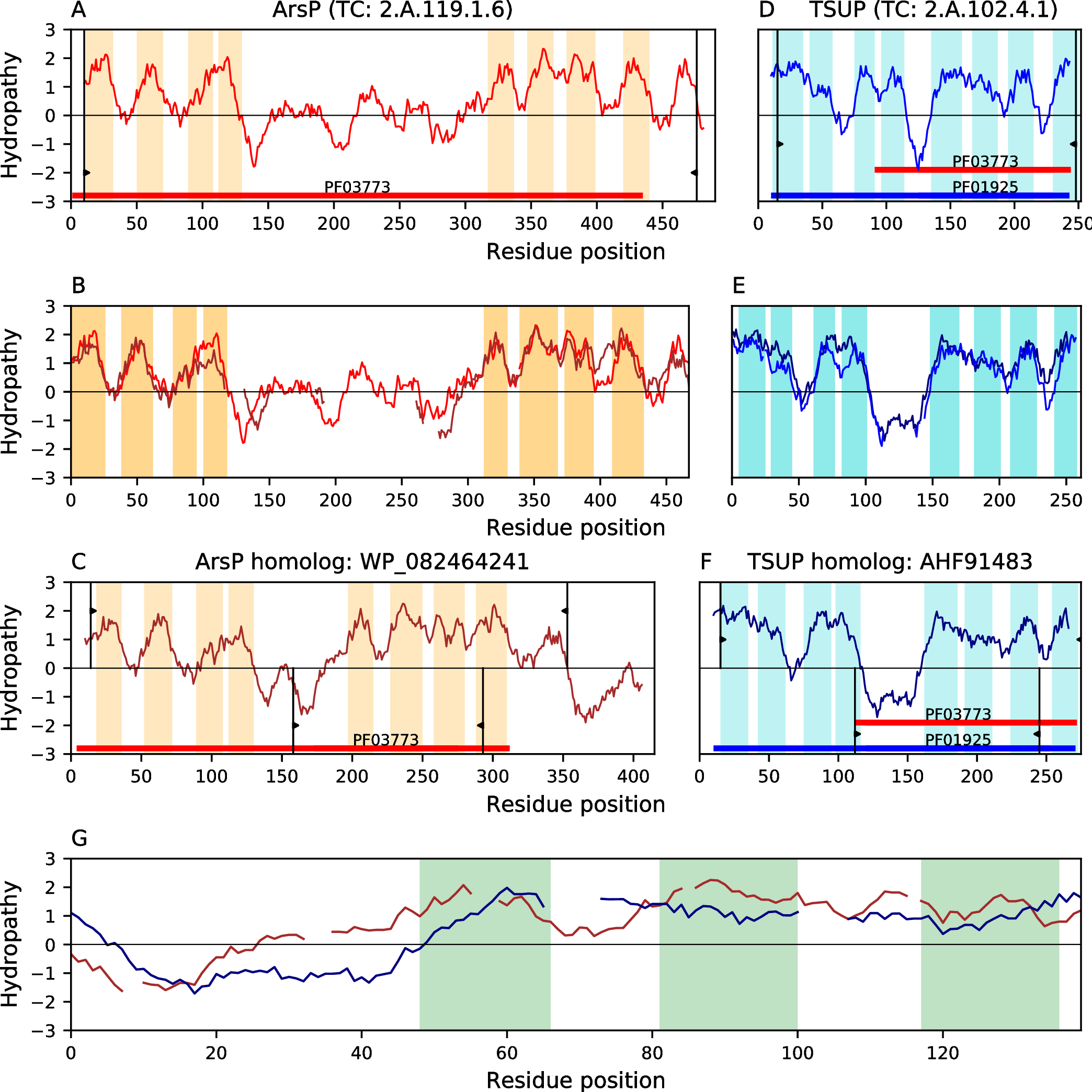

Supplement: S4 Fig — Hydropathy plots are presented across the homology transitivity path between families ArsP and TSUP. Refer to the legend of Fig 4 for a detailed description of the format. A. Hydropathy plot of ArsP member Q8EJL9 (TC: 2.A.119.1.6). B. Hydropathy plot of the alignment (E-value: 5.0×10^−47^) between ArsP member Q8EJL9 and its homologue WP_082464241. C. Hydropathy plot of ArsP homolog WP_082464241. Note that both proteins Q8EJL9 and WP_082464241 share two properties: 1) the third hydrophobic peak is composed of two TMSs; and 2) there is an extra (not colored) C-terminal hydrophobic peak. Both properties can be easily observed from alignments with other ArsP members, for example WP_069955515 (TC: 2.A.119.1.5), and by the regions covered by the Pfam domain PF03773. D. Hydropathy of TSUP member Q9UYH7 (TC: 2.A.102.4.1). E. Hydropathy of the alignment (E-value: 3.2×10^−28^) between TSUP member Q9UYH7 and its homologue AHF91483. F. Hydropathy of TSUP homolog AHF91483. G. Hydropathy of the 3-TMS alignment (E-value: 8.5×10^−11^) between ArsP homologue WP_082464241and TSUP homologue AHF91483. Only the regions where hydrophobic peaks overlap are highlighted in the alignments. The full alignment in panel G is covered by the Pfam domains of both proteins, and the domain (PF03773) in WP_082464241 can be projected to AHF91483 (E-value: 3×10^−6^), further supporting the relationship between the two families. (TIF) [file pone.0231085.s007.tif]

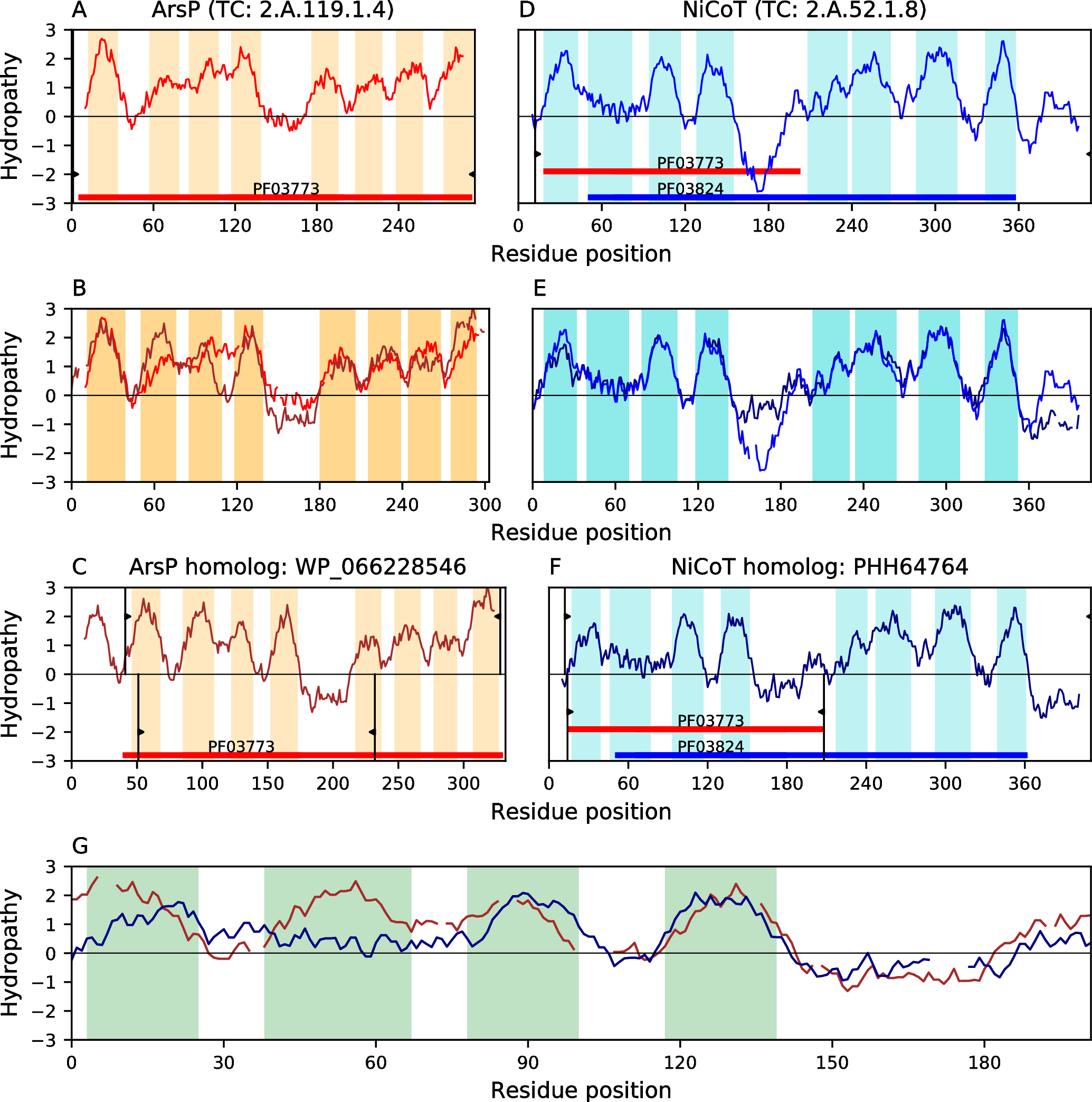

Supplement: S5 Fig — Hydropathy plots are presented across the homology transitivity path between families ArsP and NiCoT. Refer to the legend of Fig 4 for a detailed description of the format. A. Hydropathy plot of ArsP member WP_099137450 (TC: 2.A.119.1.4). B. Hydropathy plot of the alignment (E-value: 1.8×10^−13^) between ArsP member WP_099137450 and its homologue WP_066228546. C. Hydropathy plot of ArsP homolog WP_066228546. Note the extra N-terminal TMS in protein WP_066228546, which can be easily observed both from the region that aligns with member WP_099137450 and the region covered by the Pfam domain PF03773. D. Hydropathy plot of NiCoT member Q7S3L8 (TC: 2.A.52.1.8). E. Hydropathy plot of the alignment (E-value: 6.8×10^−99^) between NiCoT member Q7S3L8 and its homologue PHH64764. F. Hydropathy of NiCoT homolog PHH64764. G. Hydropathy plot of the 4-TMS alignment (E-value: 2.1×10^−8^) between ArsP homologue WP_066228546 and NiCoT homologue PHH64764. Only the regions where hydrophobic peaks overlap are highlighted in the alignments. The alignment in panel G is covered by the Pfam domains of both proteins, and the domain (PF03773) in WP_066228546 can be projected to PHH64764 (E-value: 1.4×10^−5^) further supporting the relationship between these two families. (TIF) [file pone.0231085.s008.tif]

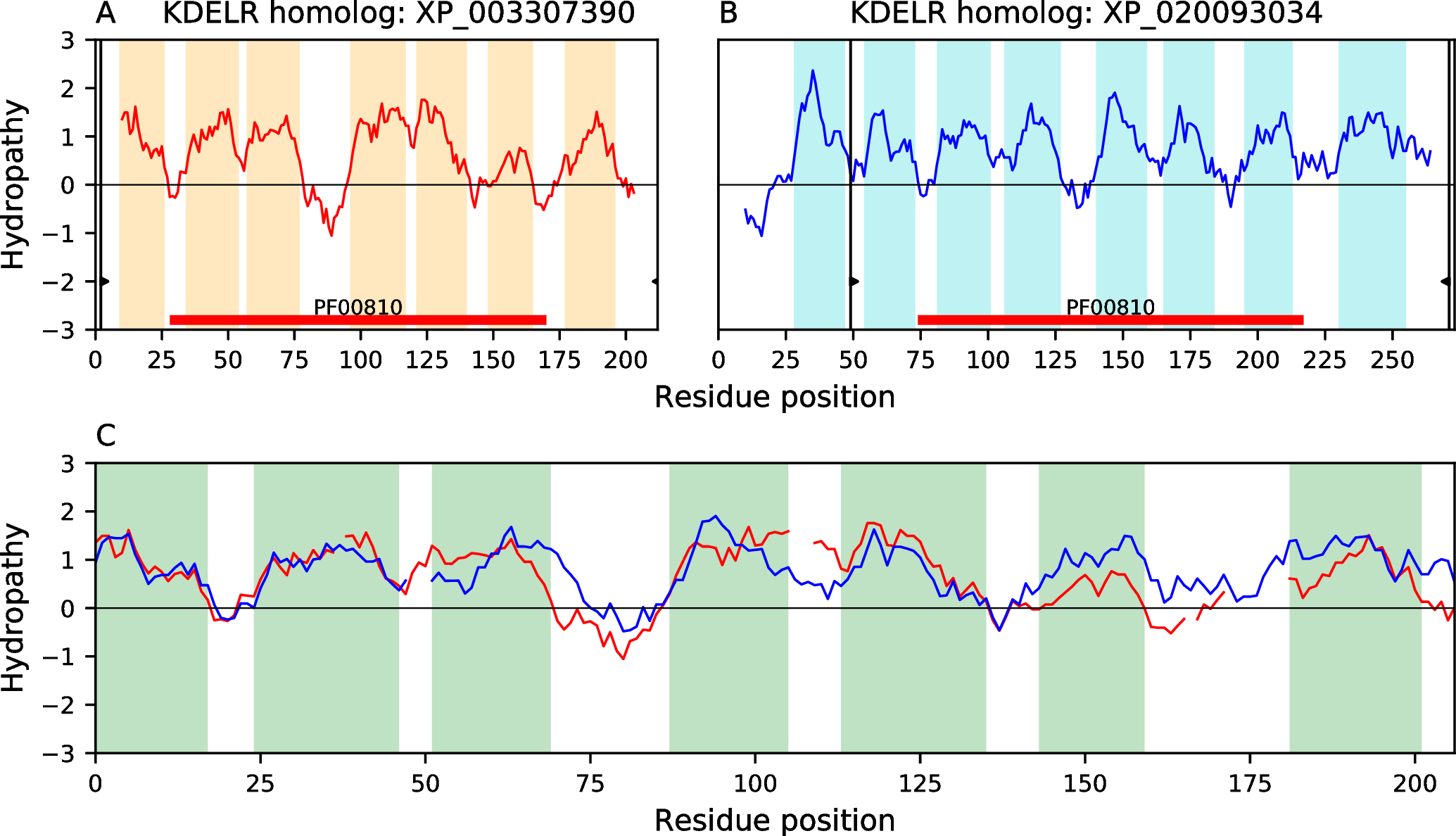

Supplement: S6 Fig — A. Hydropathy plot of 7-TMS KDELR homolog XP_003307390. B. Hydropathy plot of 8-TMS KDELR homolog XP_020093034. C. Hydropathy plot of the alignment (E-value: 2.0×10^−22^) between XP_003307390 (red) and XP_020093034 (blue). Interruptions in the hydropathy curves of panel C indicate gaps in the sequence alignment. Thin black vertical lines with wedges in panels A-B delimit the regions of these proteins involved in the alignment presented in panel C. Notice that the alignment contains only hydrophobic peaks 2–8 in XP_020093034. The exclusion of the first hydrophobic peak in XP_020093034 from the alignment provides evidence supporting the loss of the N-terminal TMS in XP_003307390 and other 7-TMS family members. (TIF) [file pone.0231085.s009.tif]

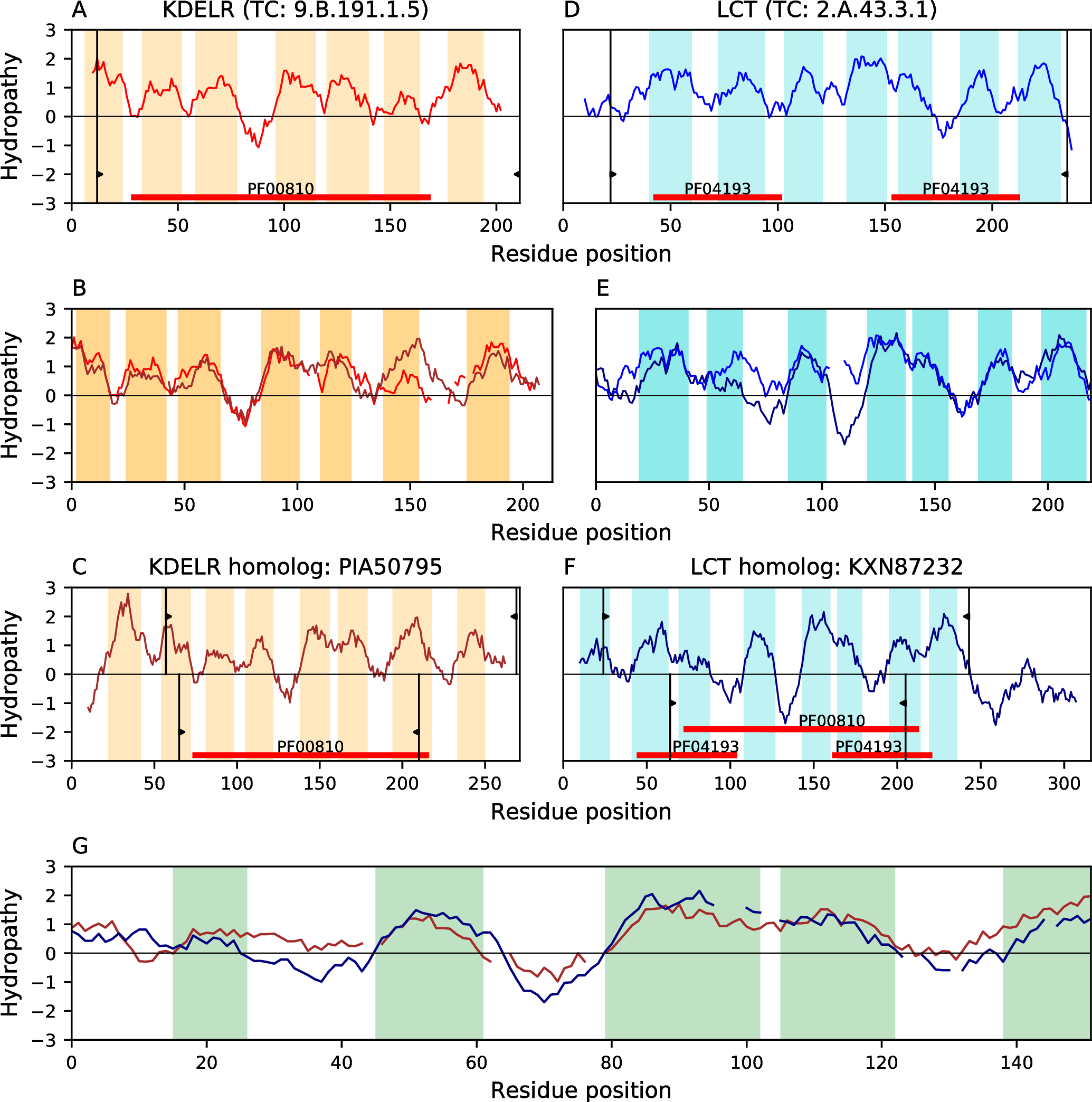

Supplement: S7 Fig — Hydropathy plots are presented across the homology transitivity path between families KDELR and LCT. Refer to the legend of Fig 4 for a detailed description of the format. A. Hydropathy plot of 7-TMS KDELR member P24390 (TC: 9.B.191.1.5). B. Hydropathy plot of the 7-TMS alignment (E-value: 6.0×10^−20^) between KDELR member P24390 and its homologue PIA50795. C. Hydropathy plot of KDELR homolog PIA50795. Note that the alignment starts in the second hydrophobic peak of homolog PIA50795, which further supports the loss of the N-terminal TMS from KDELR homologs with 7 TMSs. D. Hydropathy plot of LCT member Q60441 (TC: 2.A.43.3.1). E. Hydropathy plot of the alignment (E-value: 7.9×10^−31^) between LCT member Q60441 and its homologue KXN87232. F. Hydropathy of LCT homolog KXN87232. G. Hydropathy plot of the 5-TMS alignment (E-value: 3.1×10^−9^) between KDELR homolog PIA50795 and LCT homolog KXN87232. Only the regions where hydrophobic peaks overlap are highlighted in the alignments. The alignment in panel G includes most of the Pfam domains of both proteins. In addition, KDELR domain (PF00810) is directly found in LCT homologue KXN87232 (hmmscan E-value: 5.3×10^−5^) without the need of projection, further supporting the relationship between both families. (TIF) [file pone.0231085.s010.tif]

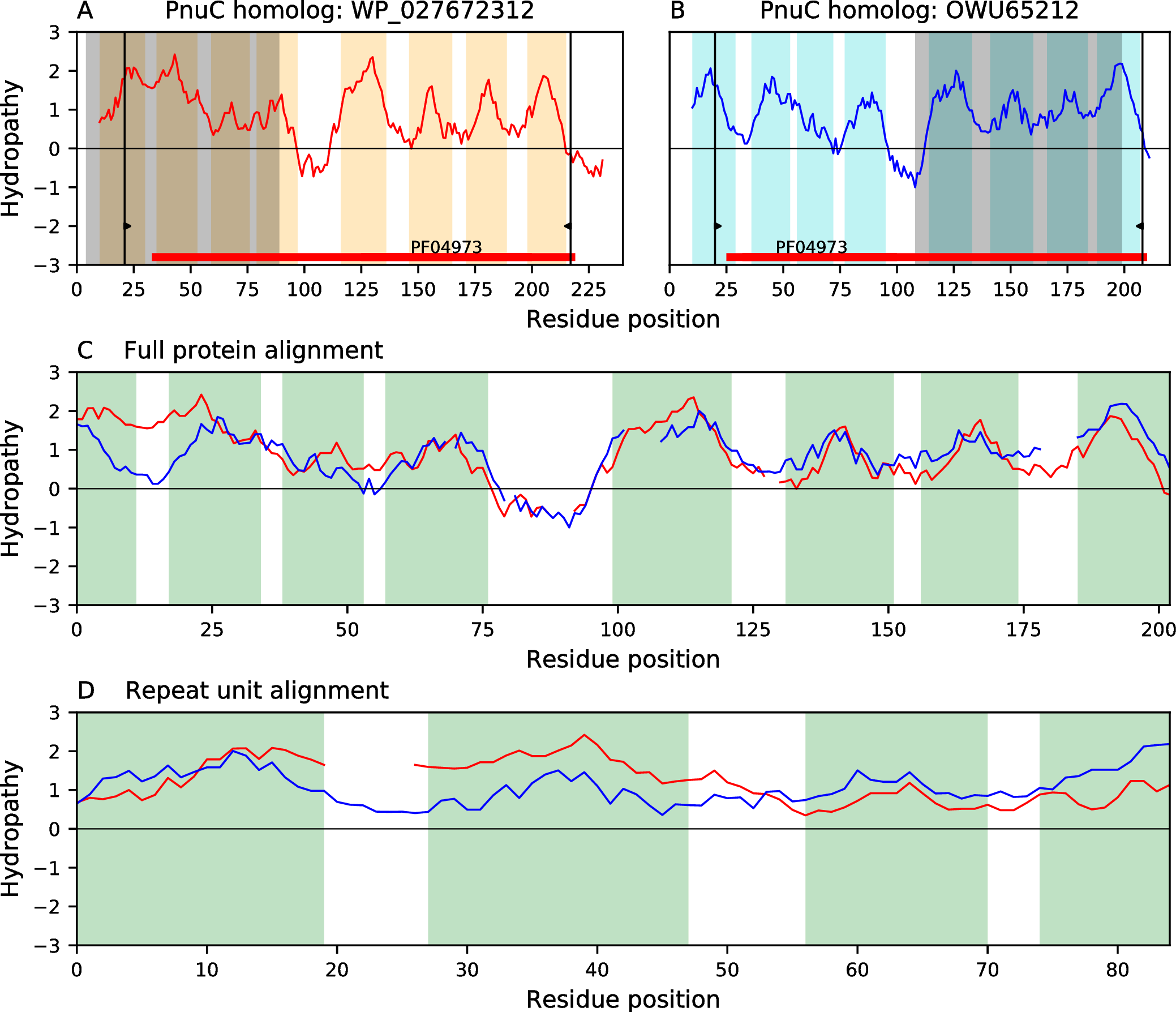

Supplement: S8 Fig — A representative alignment between 8-TMS proteins WP_027672312 and OWU65212 illustrates the 4-TMS repeat unit in PnuC as identified by AncientRep [32]. Thin vertical black lines with wedges delimit the regions involved in the alignment of the two full-length proteins. Orange and cyan bars highlight hydrophobicity peaks (i.e., inferred TMSs), respectively, for both proteins. A. Hydropathy plot of protein WP_027672312. TMSs 1–4 (shaded in dark gray) participate in the alignment shown in panel D. B. Hydropathy plot of protein OWU65212. Hydrophobic peaks 5–8 (shaded in dark gray) participate in the alignment shown in panel D. C. Hydropathy plot of the alignment (E-value: 4.3×10^−14^) between the full proteins. D. Hydropathy plot of the 4-TMS alignment (E-value: 3.7×10^−7^) that provides evidence for the repeat. The good overlap of the hydropathy curves increases the significance of the alignment. Interruptions in the hydropathy curves of panels C and D indicate gaps in the corresponding sequence alignments. (TIF) [file pone.0231085.s011.tif]

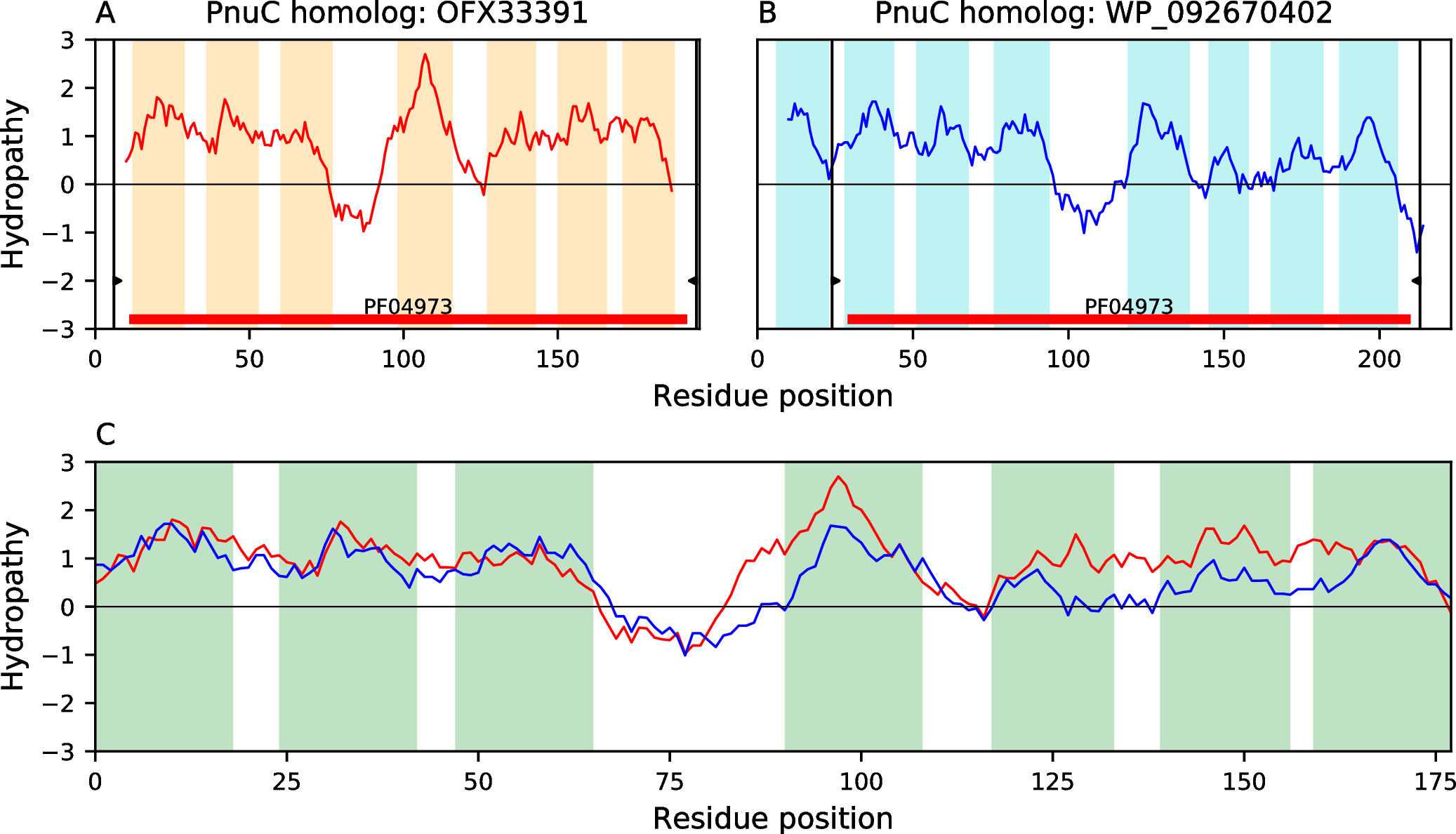

Supplement: S9 Fig — Representative alignment supporting the loss of the N-terminal TMS in PnuC members with 7 TMSs. A. Hydropathy of 7-TMS PnuC homolog OFX33391. B. Hydropathy of 8-TMS PnuC homolog WP_092670402. C. Hydropathy of the alignment (E-value: 3.0×10^−23^) between OFX33391 (red) and WP_092670402 (blue). Interruptions in the hydropathy curves of panel C indicate gaps in the sequence alignment. Thin black vertical lines with wedges in panels A and B delimit the regions of these proteins involved in the alignment presented in panel C. The loss of the N-terminal TMS in homolog OFX33391 is supported by the fact that the first TMS in WP_092670402 (panel B) is not part of the alignment shown in Panel C. (TIF) [file pone.0231085.s012.tif]

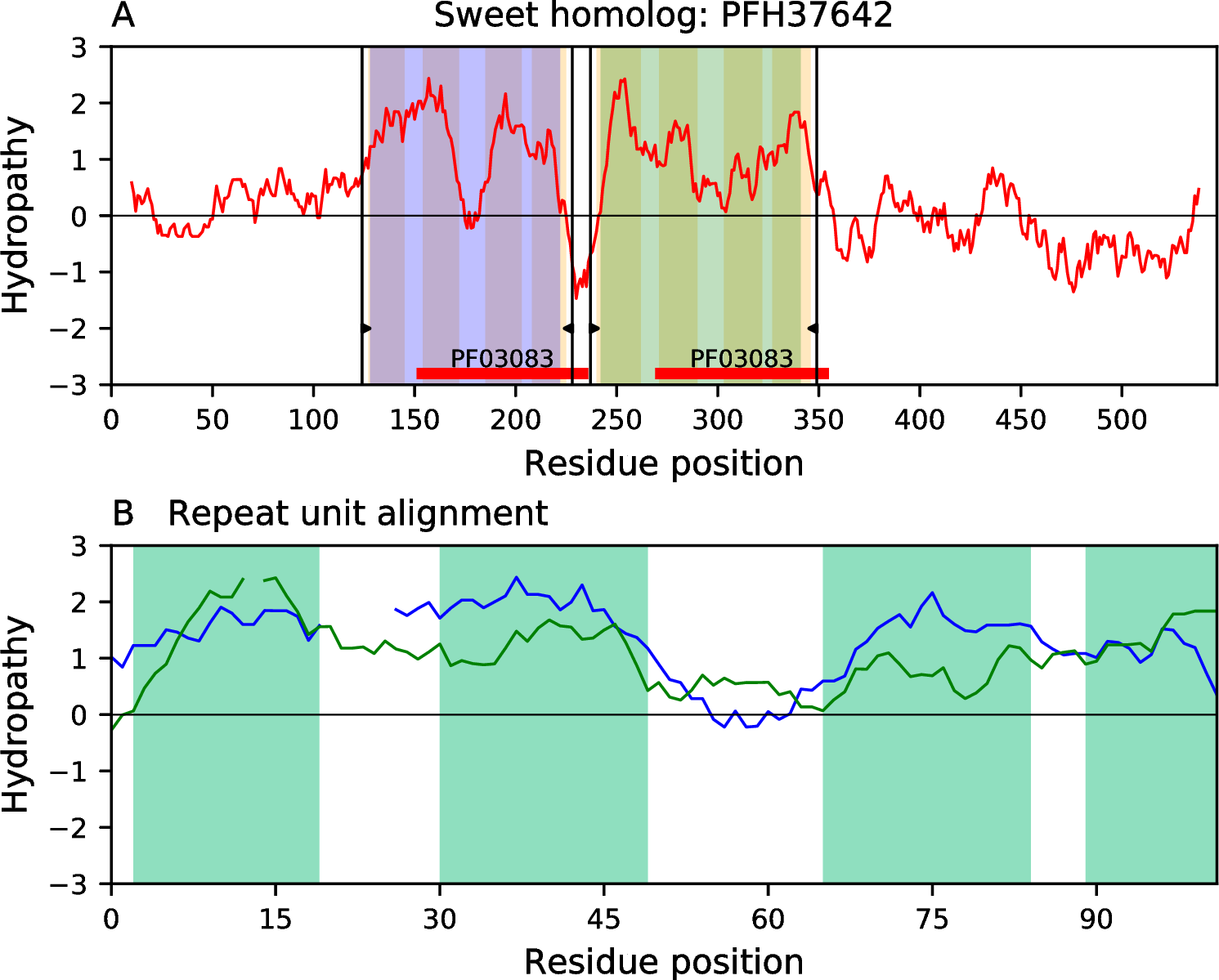

Supplement: S10 Fig — The repeat unit of Sweet members with 7 TMS is 3+1+3 where TMSs 1–3 are highly similary to TMSs 5–7. Here we present a representative example of a Sweet homolog with 8 TMSs showing a 4-TMS repeat as identified by our program tmsRepeat (see Methods). A. Hydropathy plot of the 8-TMS Sweet homolog PFH37642. Thin black vertical lines with wedges delimit the two 4-TMSs bundles that were initially aligned. The regions within the 4-TMS bundles that were aligned by SSEARCH [27] (see Methods) are shaded blue and green, respectively. B. Hydropathy plot showing the alignment (E-value: 1.7×10^−5^) between TMSs 1–4 (blue) and TMSs 5–8 (green). Interruptions in the hydropathy curves indicate gaps in the sequence alignment. (TIF) [file pone.0231085.s013.tif]

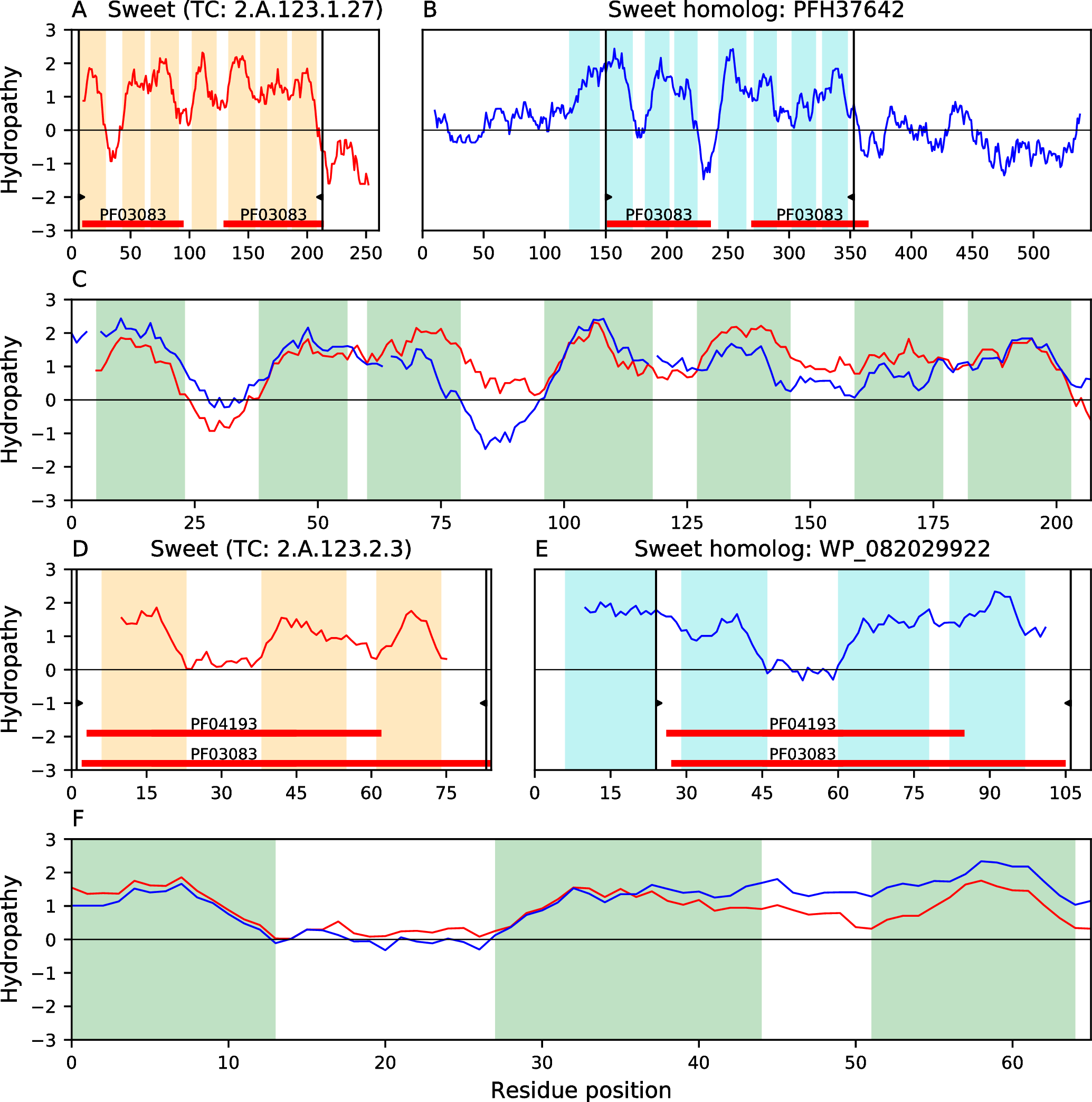

Supplement: S11 Fig — Representative alignments of Sweet homologs supporting the loss of the N-terminal TMS from original proteins with 8 (panels A-C) and 4 TMSs (panels D-F). The thin black bars with wedges in panels A, B, D, and E, delimit the regions of the proteins that participate in the alignments shown in panels C and F. Interruptions in the hydropathy curves of panels C and F indicate gaps in the corresponding sequence alignments. A. Hydropathy plot of 7-TMS Sweet member ANC68268 (TC: 2.A.123.1.27). B. Hydropathy plot of 8-TMS Sweet homolog PFH37642. This is the same protein used in S10 Fig to identify the 4-TMS repeat unit. C. Hydropathy plot of the alignment (E-value: 1.9×10^−19^) between ANC68268 and PFH37642. D. Hydropathy plot of 3-TMS Sweet member C3WG44 (TC: 2.A.123.2.3). E. Hydropathy plot of 4-TMS Sweet homolog WP_082029922. F. Hydropathy plot of the alignment (E-value: 7.1×10^−16^) between ANC68268 and PFH37642. The loss of the N-terminal TMS is supported by the fact that the first TMS in proteins PFH37642 and WP_082029922 is not part of their respective alignments, and that all TMSs in proteins ANC68268 and C3WG44 are aligned. (TIF) [file pone.0231085.s014.tif]

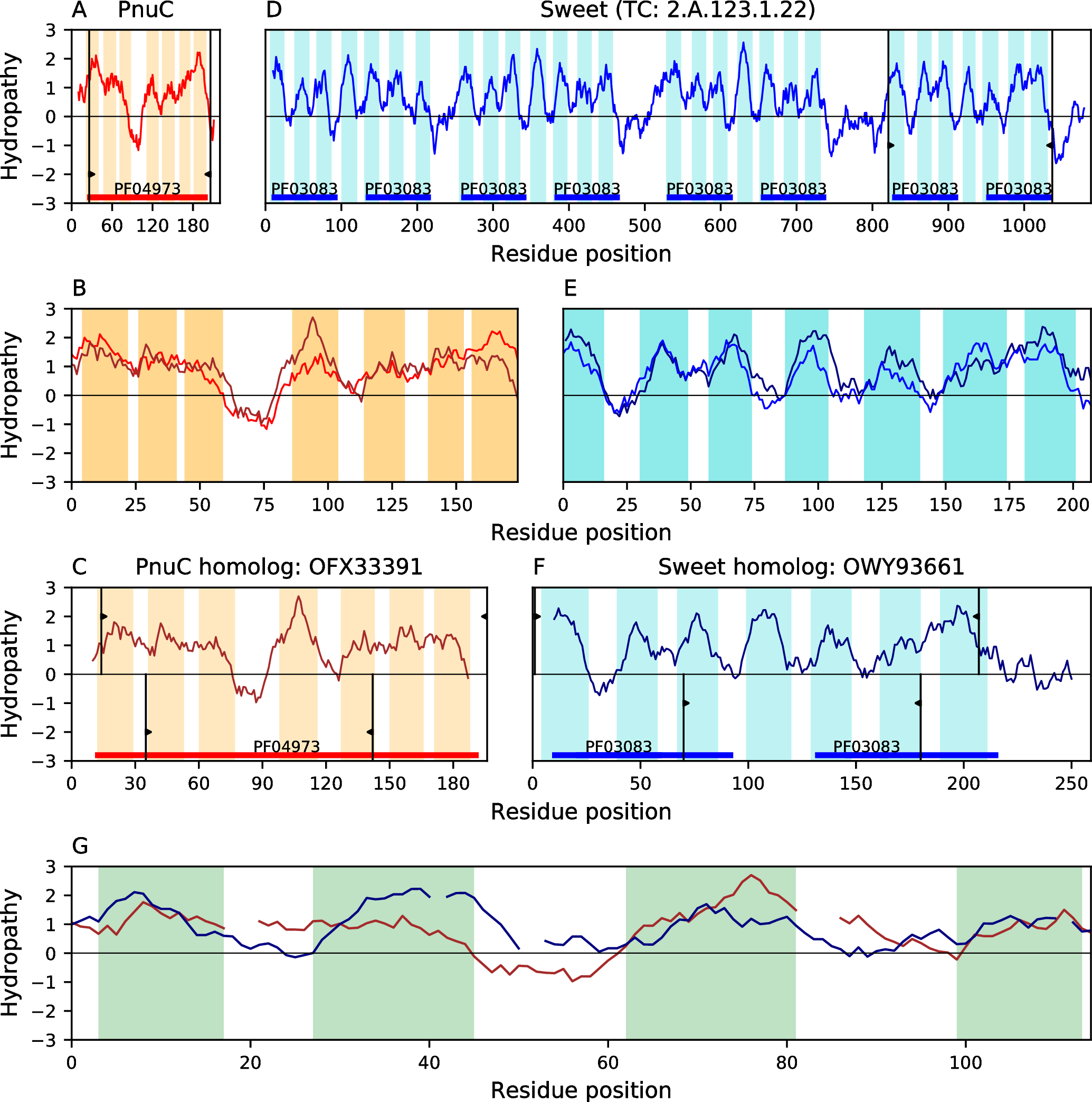

Supplement: S12 Fig — Hydropathy plots are presented across the homology transitivity path between families PnuC and Sweet. Refer to the legend of Fig 4 for a detailed description of the format. A. Hydropathy plot of 7-TMS PnuC member Q8EDN0 (TC: 4.B.1.1.4). B. Hydropathy plot of the 7-TMS alignment (E-value: 6.7×10^−22^) between Q8EDN0 and its homologue OFX33391. C. Hydropathy plot of 7-TMS PnuC homolog OFX33391. D. Hydropathy plot of Sweet member H3GD93 (TC: 2.A.123.1.22). This protein consists of the quadruplication of a 7-TMS precursor protein. E. Hydropathy plot of the 7-TMS alignment (E-value: 3.5×10^−52^) between H3GD93 and its homologue OWY93661. Note that OWY93661 has significant alignments (E-value < 10−30) with all 4 repeats in H3GD93; however, we present only the top scoring alignment with the fourth repeat as indicated in panel D. F. Hydropathy of the Sweet homolog OWY93661. G. Hydropathy plot of the 4-TMS alignment (E-value: 1.8×10^−8^) between PnuC homolog OFX33391 and Sweet homolog OWY93661. Only the regions where hydrophobic peaks overlap are highlighted in the alignments. The alignment of hydrophobic peaks between OFX33391 (peaks 2–5) and OWY93661 (peaks 3–7) is not consistent with their common topologies (3+1+3 or 3+4), suggesting that the evolution of the TMS architecture in family PnuC followed a different path as compared to families in the TOG superfamily, possibly involving an internal rearrangement of TMSs [35]. (TIF) [file pone.0231085.s015.tif]

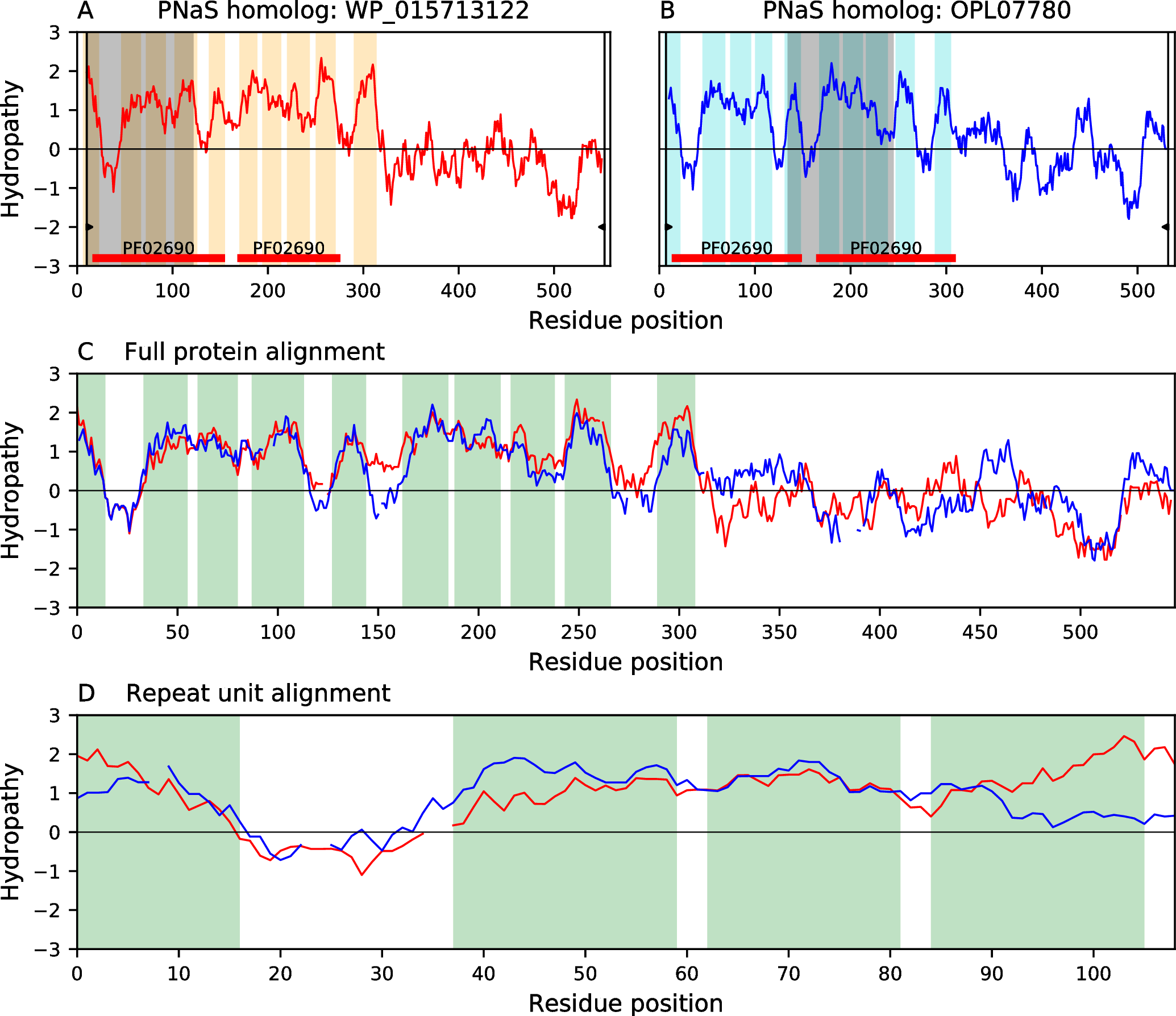

Supplement: S13 Fig — A representative alignment between proteins WP_015713122 and OPL07780 with 10 hydrophobic peaks each, suggests the 4-TMS repeat unit in PNaS as identified by AncientRep [32]. Note that HMMTOP [29] predicts 9 TMSs in both proteins, but comparisons with other family members support the presence of 10 TMSs. Thin black vertical lines and wedges delimit the regions involved in the alignment of the two full-length proteins. Orange and cyan bars highlight hydrophobicity peaks (i.e., inferred TMSs), respectively, for both proteins. A. Hydropathy plot of protein WP_015713122. TMSs 1–4 (shaded in dark gray) participate in the alignment shown in panel D. B. Hydropathy plot of protein OPL07780. TMSs 5–8 (shaded in dark gray) participate in the alignment shown in panel D. C. Hydropathy plot of the alignment (E-value: 1.2×10^−40^) between the full proteins. D. Hydropathy plot of the 4-TMS alignment (E-value: 4.1×10^−12^) that provides evidence for the repeat. Interruptions in the hydropathy curves of panels C and D indicate gaps in the corresponding sequence alignments. (TIF) [file pone.0231085.s016.tif]

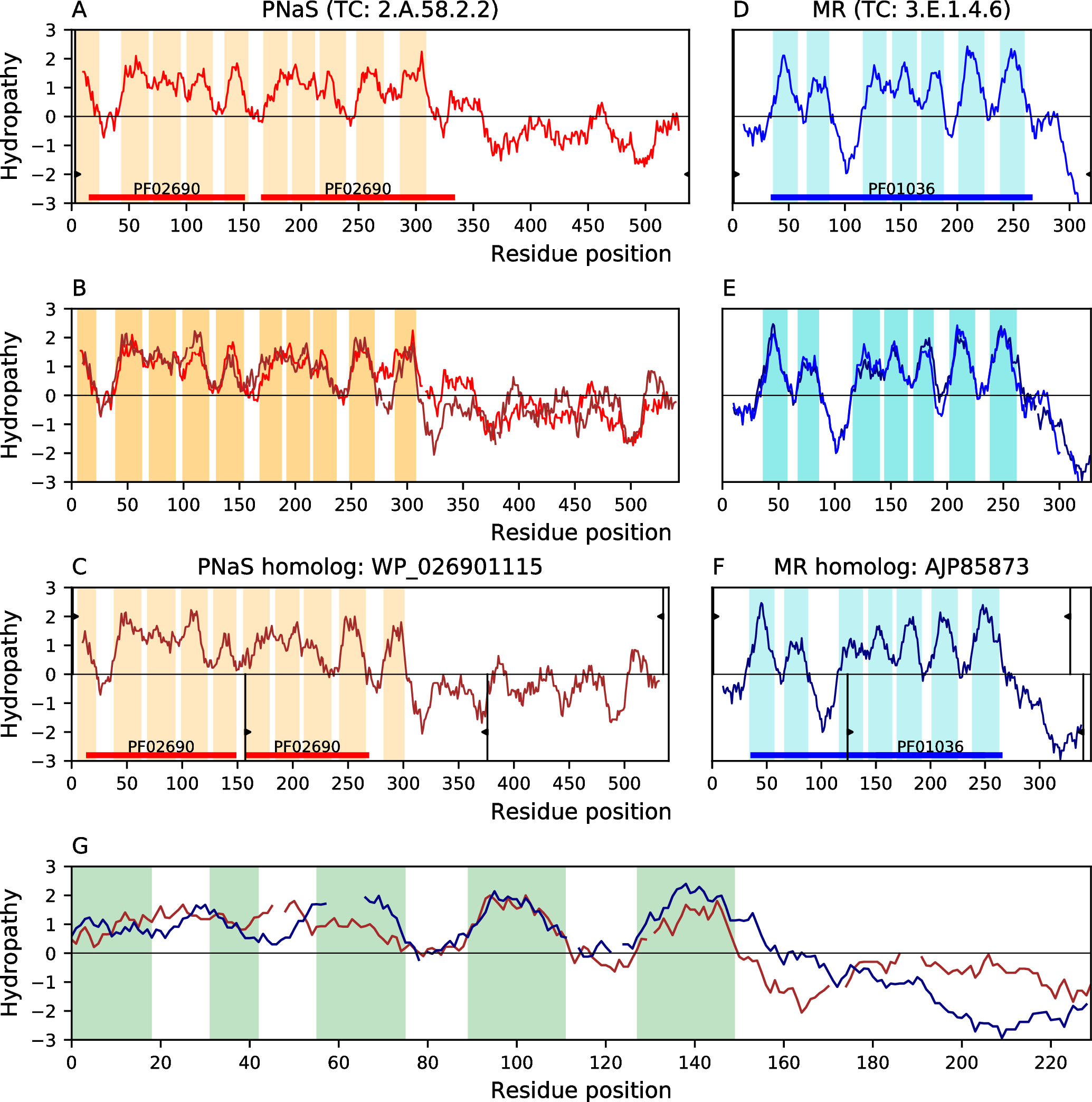

Supplement: S14 Fig — Hydropathy plots are presented across the homology transitivity path between families PNaS and MR. Refer to the legend of Fig 4 for a detailed description of the format. A. Hydropathy plot of PNaS member M7AKZ4 (TC: 2.A.58.2.2). B. Hydropathy plot of the alignment (E-value: 3.6×10^−46^) between M7AKZ4 and its homolog WP_026901115. C. Hydropathy plot of PNaS homolog WP_026901115. D. Hydropathy plot of MR member Q12117 (TC: 3.E.1.4.6). E. Hydropathy plot of the 7-TMS alignment (E-value: 1.2×10^−88^) between Q12117 and its homologue AJP85873. F. Hydropathy of the MR homolog AJP85873. G. Hydropathy plot of the 5-TMS alignment (E-value: 1.7×10^−8^) between PNaS homolog WP_026901115 and MR homolog AJP85873. Only the regions where hydrophobic peaks overlap are highlighted in the alignments. Note that 1) relative to the full protein WP_026901115 (panel C), the alignment in panel G starts in TMS 6, or the second TMS of the second 4-TMS repeat unit, and relative to AJP85873 (panel F) the alignment starts on the third TMS, or TMS 4 of the first 4-TMS repeat unit considering the loss of the N-terminal TMS in family MR [8]; 2) in panel G the second TMS of both proteins show little overlap; and 3) TMSs 9–10 of WP_026901115, which are not part of the 4-TMS repeat unit in PNaS (S13 Fig), are aligning with TMSs 7–8 of AJP85873. Thus, this alignment is not supportive of a common origin. (TIF) [file pone.0231085.s017.tif]

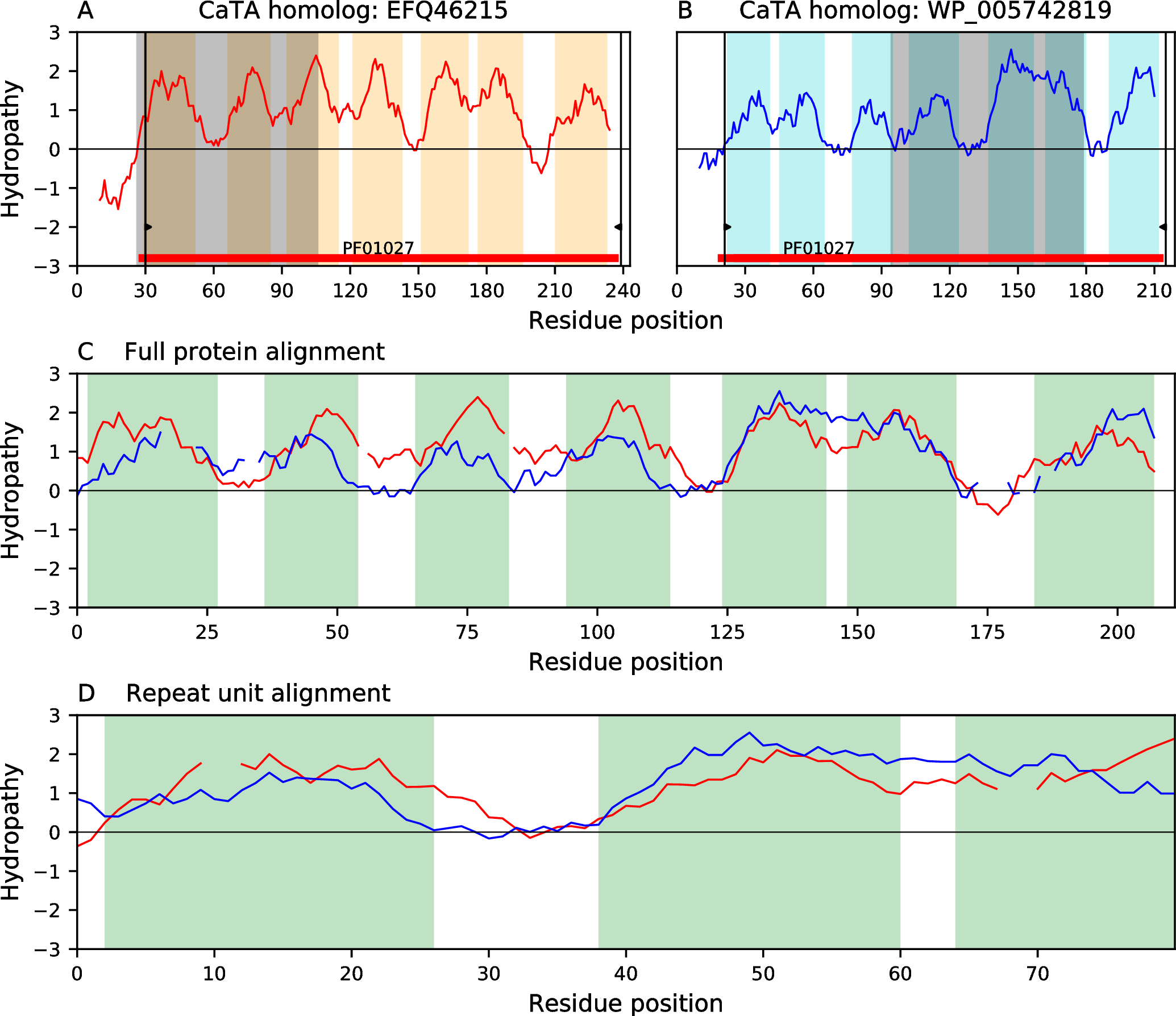

Supplement: S15 Fig — A representative alignment between proteins EFQ46215 and WP_005742819 supporting the TMS topology 3+3+1 in agreement with available 3D structural data. Thin black bars with wedges delimit the regions involved in the alignment of the two full-length proteins. Orange and cyan bars highlight hydrophobicity peaks (i.e., inferred TMSs) for each full protein, respectively. A. Hydropathy plot of protein EFQ46215. TMSs 1–3 (shaded in dark gray) participate in the alignment shown in panel D. B. Hydropathy plot of protein WP_005742819. TMSs 4–6 (shaded in dark gray) participate in the alignment shown in panel D. C. Hydropathy plot of the alignment (E-value: 1.8×10^−4^) between the full proteins. D. Hydropathy plot of the 3-TMS alignment (E-value: 5.1×1−6) that provides evidence for the repeat. Interruptions in the hydropathy curves of panels C and D indicate gaps in the corresponding sequence alignments. (TIF) [file pone.0231085.s018.tif]

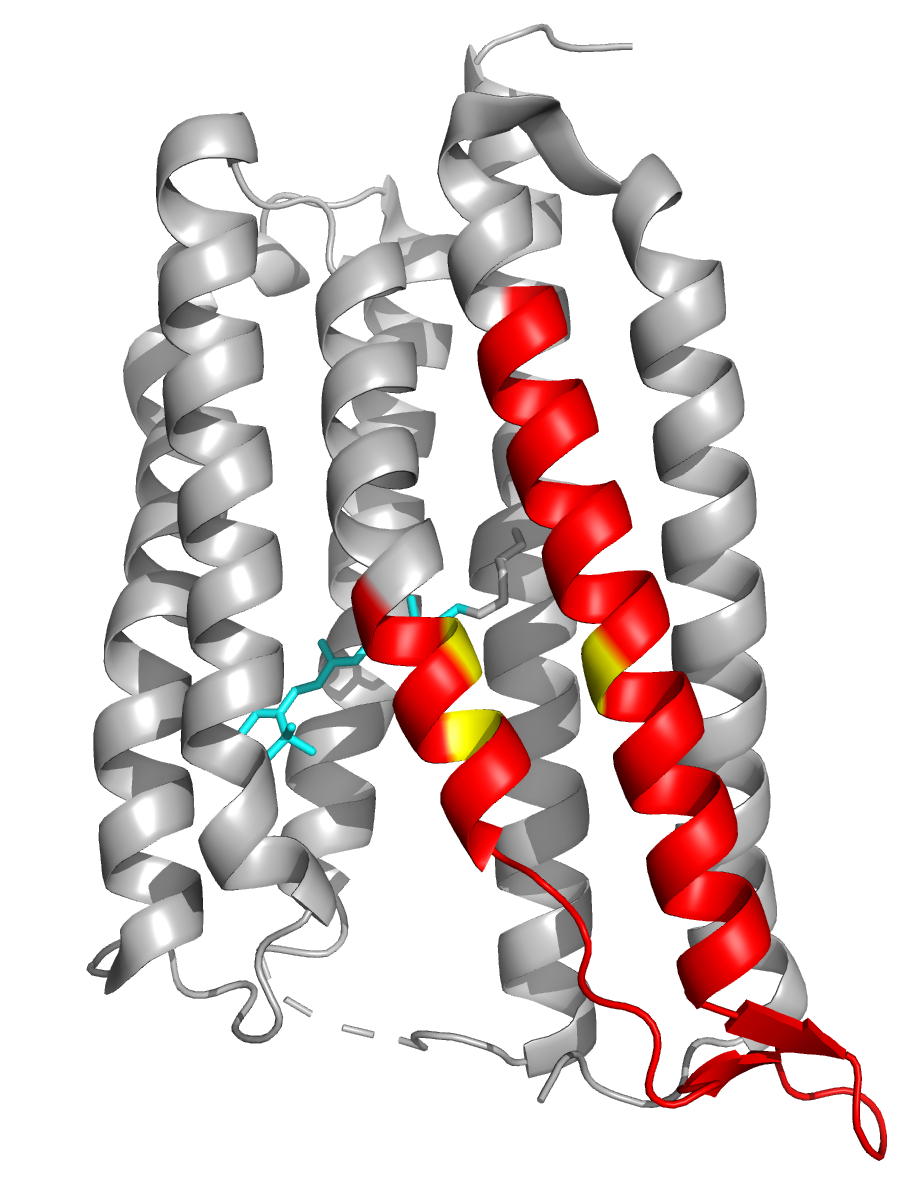

Supplement: S16 Fig — Cartoon representation of the 7-TMS xanthorhodopsin structure in Salinibacter ruber (PDB: 3DDL). The segment of TMSs 1–2 matched by MAST is shown in red, the retinal cofactor is shown in cyan, and residues making contact with retinal (< 6 Å) within the MAST region are shown in yellow. All other residues are grayed. (TIF) [file pone.0231085.s019.tif]

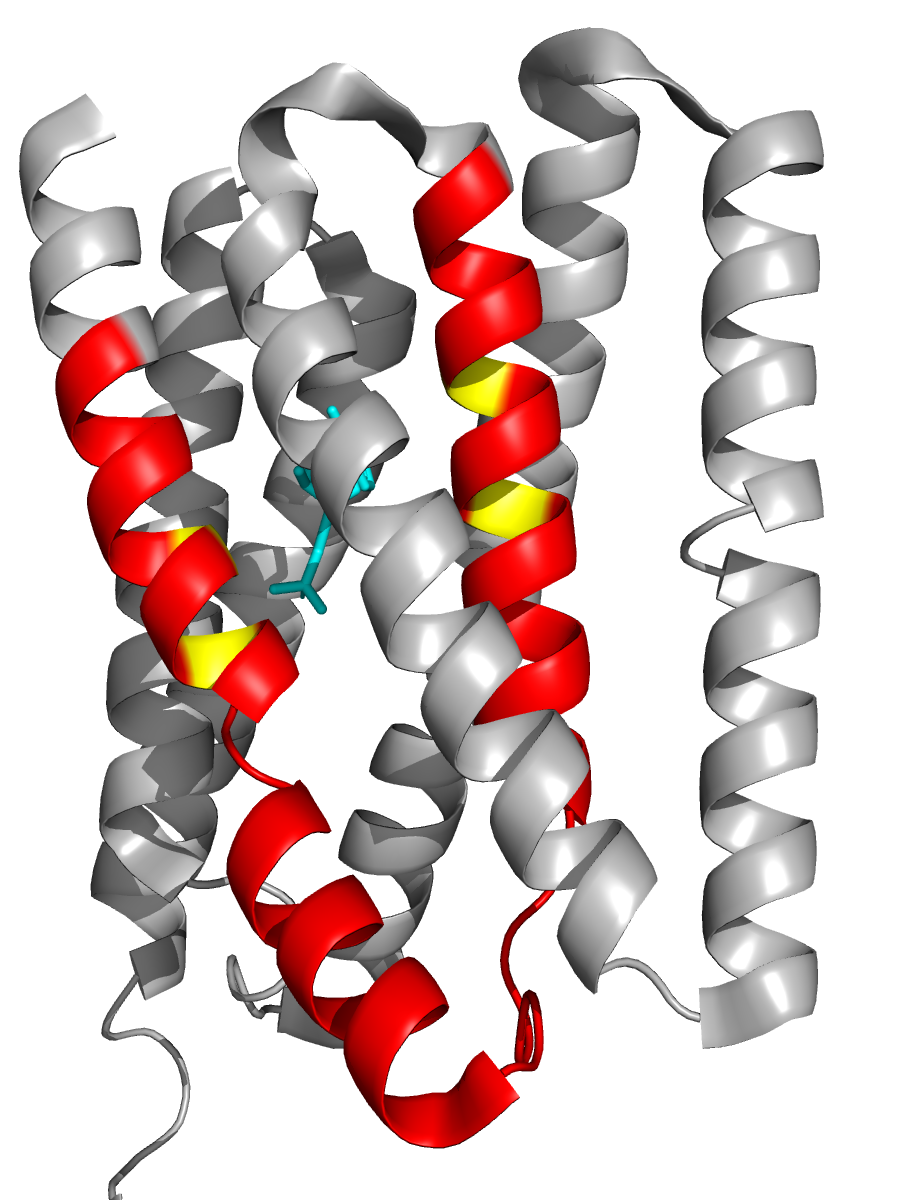

Supplement: S17 Fig — Cartoon representation of the 7-TMS SWEET13 structure in Arabidopsis thaliana (PDB: 5XPD). The segment of TMSs 1–2 matched by MAST is shown in red, the substrate analog, 2’ deoxycytidine 5’ monophosphate, is shown in cyan, and residues making contact with the substrate within the MAST region are shown in yellow. All other residues are grayed. (TIF) [file pone.0231085.s020.tif]

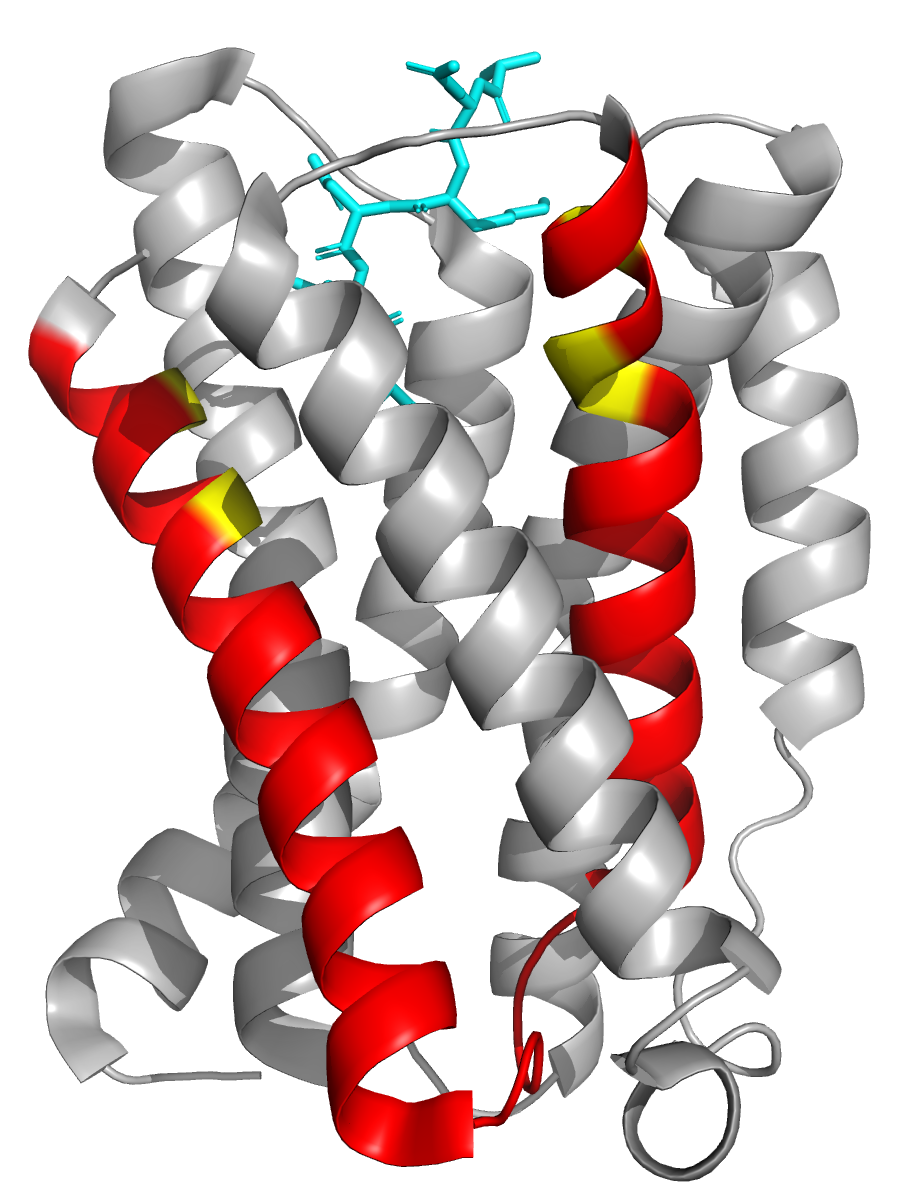

Supplement: S18 Fig — Cartoon representation of the 7-TMS KDEL receptor structure in Gallus gallus (PDB: 6I6H). The segment of TMSs 1–2 matched by MAST is shown in red, the TAEKDEL signal peptide bound to the pocket is shown in cyan, and residues making contact with the peptide within the MAST region are shown in yellow. All other residues are grayed. (TIF) [file pone.0231085.s021.tif]

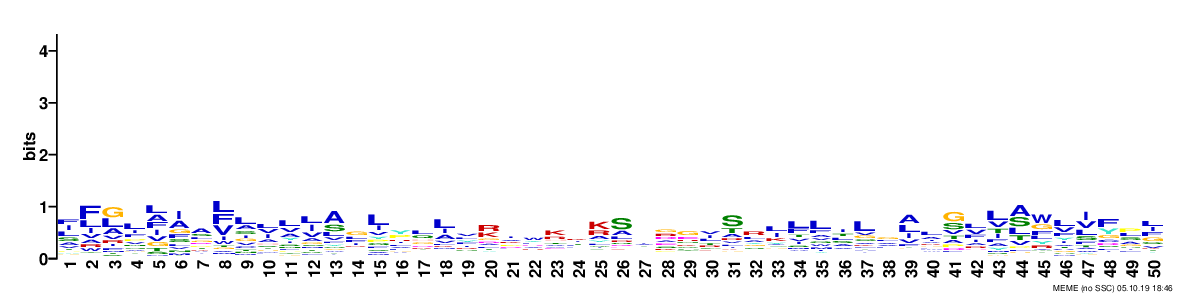

Supplement: S1 File — Folders and ouput files are compressed in a zip file. Folder MEME contains the motif models; folder MAST contains the motifs matching the training sets, and folder DATA contains both the training and test sets. Check the included README file for a detailed description. (ZIP) [file pone.0231085.s022.zip › Motifs/MEME/TOG_oops/logo1.png]

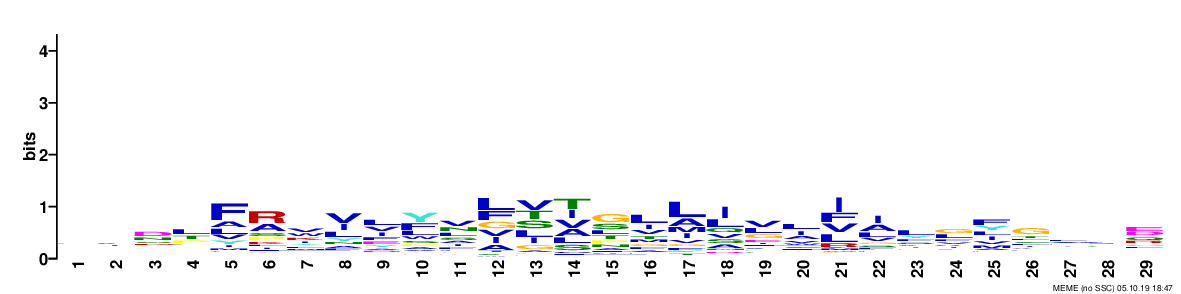

Supplement: S1 File — Folders and ouput files are compressed in a zip file. Folder MEME contains the motif models; folder MAST contains the motifs matching the training sets, and folder DATA contains both the training and test sets. Check the included README file for a detailed description. (ZIP) [file pone.0231085.s022.zip › Motifs/MEME/TOG_oops/logo2.png]

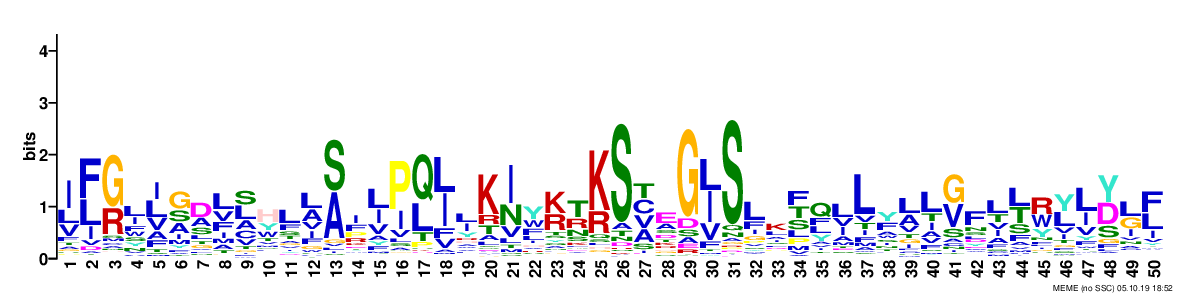

Supplement: S1 File — Folders and ouput files are compressed in a zip file. Folder MEME contains the motif models; folder MAST contains the motifs matching the training sets, and folder DATA contains both the training and test sets. Check the included README file for a detailed description. (ZIP) [file pone.0231085.s022.zip › Motifs/MEME/TOG_zoops/logo1.png]

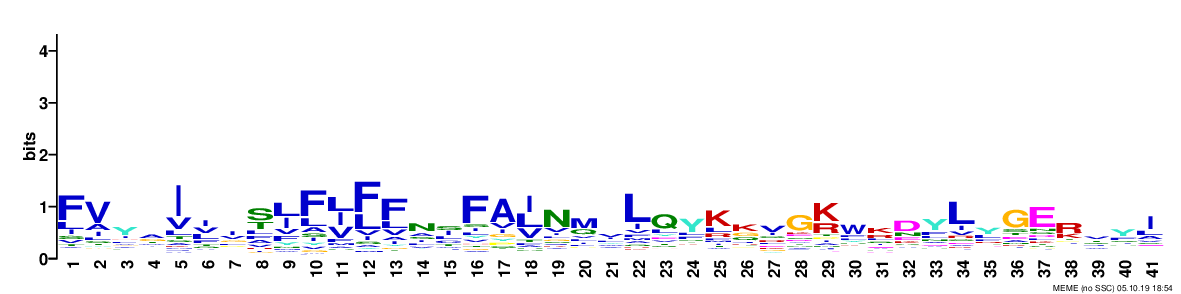

Supplement: S1 File — Folders and ouput files are compressed in a zip file. Folder MEME contains the motif models; folder MAST contains the motifs matching the training sets, and folder DATA contains both the training and test sets. Check the included README file for a detailed description. (ZIP) [file pone.0231085.s022.zip › Motifs/MEME/TOG_zoops/logo2.png]

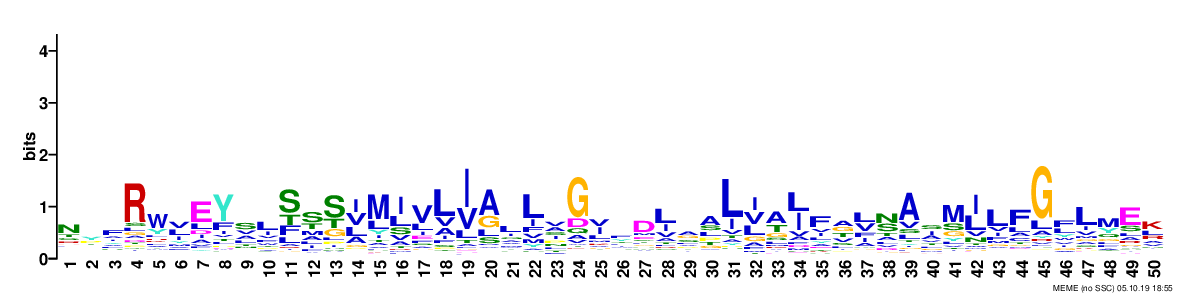

Supplement: S1 File — Folders and ouput files are compressed in a zip file. Folder MEME contains the motif models; folder MAST contains the motifs matching the training sets, and folder DATA contains both the training and test sets. Check the included README file for a detailed description. (ZIP) [file pone.0231085.s022.zip › Motifs/MEME/TOG_zoops/logo3.png]

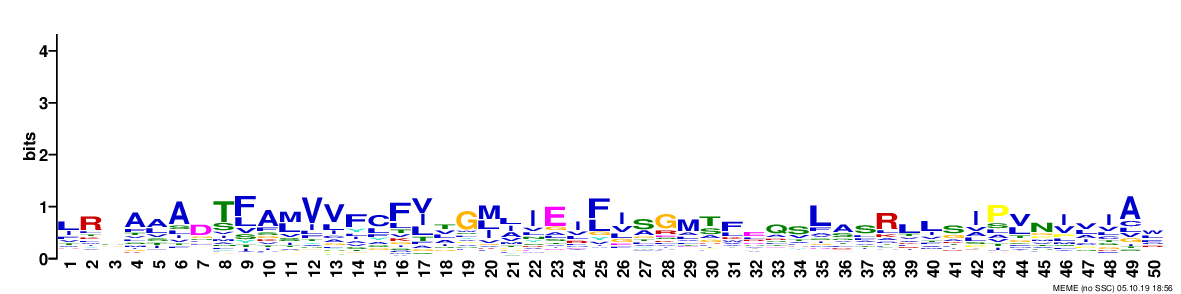

Supplement: S1 File — Folders and ouput files are compressed in a zip file. Folder MEME contains the motif models; folder MAST contains the motifs matching the training sets, and folder DATA contains both the training and test sets. Check the included README file for a detailed description. (ZIP) [file pone.0231085.s022.zip › Motifs/MEME/TOG_zoops/logo4.png]

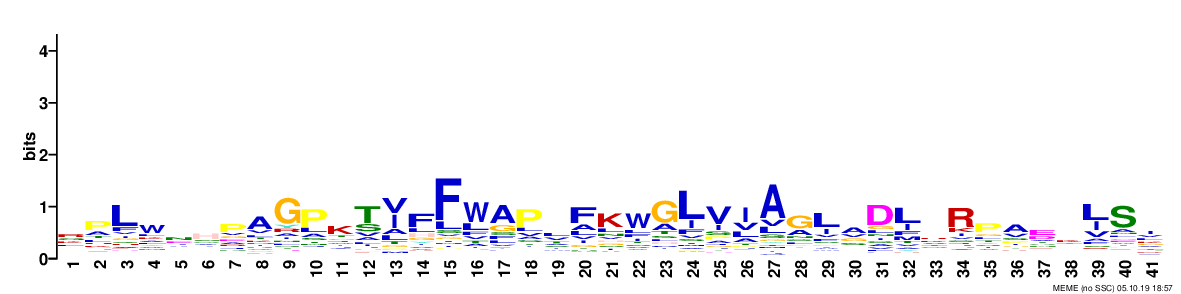

Supplement: S1 File — Folders and ouput files are compressed in a zip file. Folder MEME contains the motif models; folder MAST contains the motifs matching the training sets, and folder DATA contains both the training and test sets. Check the included README file for a detailed description. (ZIP) [file pone.0231085.s022.zip › Motifs/MEME/TOG_zoops/logo5.png]
